# Supplementary material for: RECREATE: a study protocol for a multicentre pilot cluster randomised controlled trial (cRCT) in UK stroke services evaluating an intervention to reduce sedentary behaviour in stroke survivors (Get Set Go) with embedded process and economic evaluations
Source: BMJ Open. 2023 Jul 30;13(7):e074607. doi: 10.1136/bmjopen-2023-074607 (PMC10387637; doi:10.1136/bmjopen-2023-074607)
Supplement: Supplementary data [file bmjopen-2023-074607supp001.pdf]

Appendix 1 – Participant outcome measures

Stroke survivors

- Nottingham Extended Activities of Daily Living scale (NEADL) (1): The NEADL assesses aspects of physical and social independence performance across 22 items (score range 0–66) grouped in four categories (mobility, kitchen, domestic and leisure activities), with higher scores indicating greater independence.
- World Health Organisation Disability Assessment Schedule 2.0 (12-item; WHODAS 2.0-short) (2-6). The WHODAS 2.0-short (2) was designed as a self-administered questionnaire which asks about difficulties due to health conditions. Items are scored on a 5-point Likert scale (from 0 (none) to 4 (extreme) and summed, then converted into a percentage, with higher scores indicating greater disability. A unit increment in WHODAS 2.0 z-score increased mortality hazard (6).
- Warwick-Edinburgh Mental Well-being Scale (WEMWBS) (7). The WEMWBS is designed for self-completion and comprises 14 items relating to an individuals' state of mental well-being in the previous two weeks. The scale is scored by summing responses to each item answered on a five-point Likert scale; total scores range from 14-70, with higher scores indicating higher levels of mental well-being.
- European Quality of Life 5-Dimension Health Questionnaire (5 levels; EQ-5D-5L) (8). The non-disease-specific EQ-5D-5L instrument will be used to measure health related quality of life across five dimensions: mobility, self-care, usual activities, pain/discomfort, and anxiety/depression. Each dimension is scored on a five-point scale, from 1 (no problems) to 5 (unable/ extreme problems); scores from the five dimensions are combined into a five-digit number to describe current health state. A visual analogue scale is also provided for participants to self-rate their health. The scale was developed to yield utility values which can be used to calculate QALY gains or losses, and thus will facilitate the health economic evaluation. Participants

Airlie J, *et al. BMJ Open* 2023; 13:e074607. doi: 10.1136/bmjopen-2023-074607

are also asked to self-rate their health on the accompanying vertical Visual Analogue Scale, with the best and worst health they can imagine as endpoints.

- Fatigue Assessment Scale (FAS) (9, 10). The FAS is a 10-item questionnaire consisting of five questions relating to mental fatigue and five questions for physical fatigue. Participants are asked to rate how they usually feel on a five-point scale from 1 (never) to 5 (always), with total scores ranging from 10 to 50. Higher scores indicate increased fatigue.
- Measure of Older Adults' Sedentary Time (MOST) (11) and Sedentary Behaviour Visual Analogue Scale (adapted from (12)). The MOST asks participants to estimate the amount of time spent in seven sedentary activities during the previous week.
- Whether they have fallen and whether or not this led to injury (13). At baseline this will be related to in hospital falls and falls during the three months prior to admission
- Whether they use a walking aid
- Health and social care service use and informal care inputs

Carers

Modified Caregiver Strain Index (MCSI (14)). The MCSI is a 13-item measure of strain related to care provision covering five domains: financial; physical; psychological; social; and personal. Each item is scored 0 (no), 1 (sometimes) or 2 (yes), with total scores ranging from 0-26. Higher scores indicate higher levels of caregiver strain.

References

1. Nouri F, Lincoln N. An extended activities of daily living scale for stroke patients. Clinical rehabilitation. 1987;1(4):301-5.
2. Üstün TB, Chatterji S, Kostanjsek N, et al. Developing the World Health Organization disability assessment schedule 2.0. Bulletin of the World Health Organization. 2010;88:815-23.
3. Schlote A, Richter M, Wunderlich M, et al. WHODAS II with people after stroke and their relatives. Disability and rehabilitation. 2009;31(11):855-64.
4. Garin O, Ayuso-Mateos JL, Almansa J, et al. Validation of the" World Health Organization Disability Assessment Schedule, WHODAS-2" in patients with chronic diseases. Health and quality of life outcomes. 2010;8:1-15.
5. Küçükdeveci AA, Kutlay Ş, Yıldızlar D, et al. The reliability and validity of the World Health Organization Disability Assessment Schedule (WHODAS-II) in stroke. Disability and rehabilitation. 2013;35(3):214-20.
6. Hirve S, Juvekar S, Sambhudas S, et al. Does self-rated health predict death in adults aged 50 years and above in India? Evidence from a rural population under health and demographic surveillance. International journal of epidemiology. 2012;41(6):1719-27.
7. Tennant R, Hiller L, Fishwick R, et al. The Warwick-Edinburgh mental well-being scale (WEMWBS): development and UK validation. Health and Quality of life Outcomes. 2007;5(1):1-13.
8. Herdman M, Gudex C, Lloyd A, et al. Development and preliminary testing of the new five-level version of EQ-5D (EQ-5D-5L). Quality of life research. 2011;20:1727-36.
9. Mead G, Lynch J, Greig C, et al. Evaluation of fatigue scales in stroke patients. Stroke. 2007;38(7):2090-5.
10. Michielsen HJ, De Vries J, Van Heck GL. Psychometric qualities of a brief self-rated fatigue measure: The Fatigue Assessment Scale. Journal of psychosomatic research. 2003;54(4):345-52.

11. Gardiner PA, Eakin EG, Healy GN, et al. Feasibility of reducing older adults' sedentary time. American journal of preventive medicine. 2011;41(2):174-7.

12. Chastin SF, Dontje ML, Skelton DA, et al. Systematic comparative validation of self-report measures of sedentary time against an objective measure of postural sitting (activPAL). International Journal of Behavioral Nutrition and Physical Activity. 2018;15(1):1-12.

13. Perry L, Kendrick D, Morris R, et al. Completion and return of fall diaries varies with participants' level of education, first language, and baseline fall risk. Journals of Gerontology Series A: Biomedical Sciences and Medical Sciences. 2012;67(2):210-4.

14. Thornton M, Travis SS. Analysis of the reliability of the modified caregiver strain index. The Journals of Gerontology Series B: Psychological Sciences and Social Sciences. 2003;58(2):S127-S32.
