# Supplementary material for: RECREATE: a study protocol for a multicentre pilot cluster randomised controlled trial (cRCT) in UK stroke services evaluating an intervention to reduce sedentary behaviour in stroke survivors (Get Set Go) with embedded process and economic evaluations
Source: BMJ Open. 2023 Jul 30;13(7):e074607. doi: 10.1136/bmjopen-2023-074607 (PMC10387637; doi:10.1136/bmjopen-2023-074607)
Supplement: Supplementary data [file bmjopen-2023-074607supp002.pdf]

Appendix 2 – Information sheet for stroke survivors

[INSERT LOGOS HERE]

RECREATE
Reducing sedentary behaviour after stroke

INFORMATION SHEET FOR STROKE SURVIVORS

Helping people sit less after stroke –

COMPLETE QUESTIONNAIRES & WEAR AN ACTIVITY MONITOR

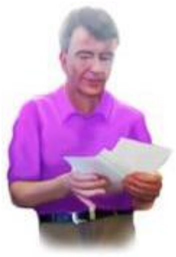
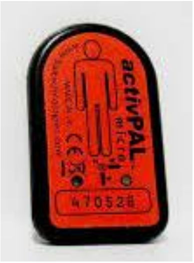

Invitation to take part in a research study

- We would like to invite you to take part in a research study.
- This leaflet tells you about the study and what your participation would involve. Please read this carefully and ask us if anything is not clear or if you would like more information.
- Please feel free to talk to other people about the study if you wish.

RECREATE PIS – SS Questionnaires Version 4.0 (05/08/2022)
ISRCTN No: 82280581 / REC reference: 19/YH/0403 / IRAS ID: 271111

What is the purpose of the study?

Research has shown that too much time spent sitting (sedentary behaviour) is bad for our health. Changes in the amount of time spent sitting may improve recovery after a stroke. The RECREATE study is exploring a new approach to help people sit less, and performing an initial test to see whether the new approach is better than “usual care”.

In this study, stroke services will be randomly allocated to either provide the new approach or continue with their “usual care”. This will enable us to compare the new approach with standard practices.

Stroke survivors have been involved throughout the design of this study, to ensure it is well designed and with people such as yourself in mind.

Why have I been invited to take part?

You are being invited to take part because you have had a stroke and are being cared for by a stroke service taking part in our study.

Do I have to take part?

No, it is up to you. You do not have to give a reason if you decide not to take part. If you decide not to take part in this study, the standard of care that you receive will not be affected in any way.

What will be involved if I agree to take part?

- A researcher will ask you some questions about yourself (e.g., your age, height and weight, whether you live alone) and will measure your blood pressure.

- You will be asked to complete some questionnaires on up to 4 occasions over the next 24 months. The first questionnaires will be provided to you when you first agree to take part in the study. We will send you the second and third questionnaires around 6 and 12 months later. We may invite you to complete a fourth questionnaire around 24 months after you agreed to take part. On each occasion these may take approximately 60 minutes to complete.

The questions will be about how you feel, what you are able to do, and use of care services, such as GP or hospital visits. It will help us understand more about your experience and the impact of your stroke over 2 years.

Someone, a friend or relative, can help you fill in the questionnaires but you must think of your own responses. This way your answers will definitely be your own. You will be provided with a pre-paid envelope to return your questionnaire.

- As well as completing the questionnaire booklet, you will be asked to wear an activity monitor and complete a sleep diary for nine days on each of the occasions.

  The activity monitor is extremely small, slim, and very light. The monitor attaches to your thigh and uses information about the position of the thigh to determine body posture (i.e., sitting/lying, standing and stepping).

  A researcher will show you how to attach the monitor and help you to do this. You will be provided with a pre-paid envelope to return the monitor and sleep diary at the end of each monitoring period. (Further information is provided in the activPAL Information Leaflet.)

- You will be sent a letter around 12 weeks after you leave hospital and may be asked to answer some questions about your recent experiences.

- You will be sent newsletters to keep you updated with the study.

What else will happen if I decide to take part?

- A researcher will access relevant parts of your medical and care records so that we can gain information about the treatment and services being provided to you. We may also collect further health and treatment information from electronic databases (e.g. NHS Digital and the Sentinel Stroke National Audit Programme).

- A researcher may telephone you, send you text messages or email you about this study (for example if we do not receive the questionnaires or activity monitor back from you).

- A researcher may ask you if they can observe some of the care you are being provided with (for example, therapy treatment) and / or contact you in a few months'

time to ask if you would like to take part in an interview so we can gain your views on the treatment and services you have received. The interview would take place at a time and a location that is convenient for you (probably your own home or via telephone) and take about an hour.

The researcher would contact you separately about this. Not everyone will be observed or contacted for an interview. If you are, you can agree or refuse to take part in the observation or interview at that point.

What are the possible benefits of taking part?

Being involved is unlikely to benefit you directly. However, it may help improve future services and support for people who have had a stroke.

What are the possible disadvantages and risks of taking part?

You may find wearing the activity monitor inconvenient. There is a small chance that it might irritate your skin. You may find some of the topics covered in the questionnaires upsetting. You do not have to answer any questions you do not wish to.

Will my taking part in the study be kept confidential?

If you decide to participate, the information collected about you will be handled strictly in accordance with the consent that you have given and also the 2018 Data Protection Act.

Members of the RECREATE research team and other authorised personnel will use your identifiable information (name, date of birth, address, telephone number and email address) to contact you about the study; make sure that relevant information about the study is recorded for your care and to oversee the quality of the study.

Your GP will be informed about your involvement in this study. Further information about how the data we collect is used is provided in the supplementary information sheet. Please read this information carefully and discuss it with your relatives and friends if you wish. Our privacy notice is available at:
https://www.bradfordhospitals.nhs.uk/privacy-statement/.

Airlie J, *et al. BMJ Open* 2023; 13:e074607. doi: 10.1136/bmjopen-2023-074607

Ask a member of the research team if anything is unclear, or if you would like more information.

What will happen if I do not want to carry on with the study?

You are free to withdraw from the study at any time, and do not have to give a reason. If you decide to stop taking part it will not affect the standard of care you receive.

If you withdraw from the study, the data collected from you up to that point will be used in analysing the results of the study. Unless you explicitly ask us not to, we will continue to collect information from your electronic health records.

What will happen to the results of the research study?

The results of this study will be published in a report and in academic journals. If you wish to obtain a copy of the report, or a lay summary of our findings, you can request one from the Trial Manager (contact details below) or visit our website using the following address: https://www.bradfordresearch.nhs.uk/our-research-teams/academic-unit-for-ageing-and-stroke-research/our-research/stroke-research/

It is also possible that the results may be presented at conferences and included in updates to clinical guidelines. You will not be identified in any report/publication.

What if I need more information or there is a problem?

If you need further information or have any concerns about any aspects of the study, please contact Seline Ozer, RECREATE Trial Manager Tel: 01274 383908 / 07870 543073, Email: recreate@bthft.nhs.uk in the first instance.

If you remain concerned and / or would like to discuss the study with someone independent of the research team you can contact the Patient Advice and Liaison Service (PALS) on: local office, Tel: xxxx).

In the unlikely event that something goes wrong and you are harmed during the research and / or you wish to raise a complaint on how we have handled your personal data, the normal NHS complaints procedures are available to you. Details can be obtained from your hospital or the PALS Service. There are no special compensation arrangements in place for this study.

Airlie J, *et al. BMJ Open* 2023; 13:e074607. doi: 10.1136/bmjopen-2023-074607

In addition, you can contact the Stroke Association [0303 3033 100] for confidential advice.

Who is organising, funding and reviewing the research?

The Chief Investigator of this research is Professor Anne Forster, who is employed by the University of Leeds and is carrying out the study from the Academic Unit for Ageing and Stroke Research at Bradford Teaching Hospitals NHS Foundation Trust.

The University of Leeds Clinical Trials Research Unit (CTRU) and King's College, London are supporting the co-ordination of the study.

The research is funded by the Department of Health (National Institute for Health Research). The sponsor for the research is Bradford Teaching Hospitals NHS Foundation Trust. This study has been reviewed by Yorkshire and The Humber – Bradford Leeds Research Ethics Committee.

FUNDED BY
NIHR | National Institute for Health and Care Research

Airlie J, *et al. BMJ Open* 2023; 13:e074607. doi: 10.1136/bmjopen-2023-074607
