# Supplementary material for: RECREATE: a study protocol for a multicentre pilot cluster randomised controlled trial (cRCT) in UK stroke services evaluating an intervention to reduce sedentary behaviour in stroke survivors (Get Set Go) with embedded process and economic evaluations
Source: BMJ Open. 2023 Jul 30;13(7):e074607. doi: 10.1136/bmjopen-2023-074607 (PMC10387637; doi:10.1136/bmjopen-2023-074607)
Supplement: Supplementary data [file bmjopen-2023-074607supp003.pdf]

Appendix 3 - Supplementary information sheet for stroke survivors

[Insert hospital logo]

RECREATE
Reducing sedentary behaviour after stroke

SUPPLEMENTARY INFORMATION SHEET FOR STROKE SURVIVORS

COMPLETE QUESTIONNAIRES and WEAR AN ACTIVITY MONITOR / OBSERVATION

Who is responsible for my data?

Bradford Teaching Hospitals NHS Foundation Trust (BTHFT) is the sponsor for this study based in the United Kingdom. We will be using information from you and your medical records in order to undertake this study and will act as "data controller". This means that we are responsible for looking after your information and using it properly. The University of Leeds Clinical Trials Research Unit (CTRU) will be supporting us in the co-ordination of this study from its premises at the University of Leeds.

How will my data be used?

This research is in the public interest, which means our results will be used to improve the healthcare of patients in the future. Because of this, if you agree to take part in the study, we will use information about you, including sensitive information about your health and ethnicity.

Members of the research team will use your personal information (name, date of birth, address, telephone number and email address) to contact you about the research study and make sure that relevant information about the study is recorded for your care. Other authorised personnel may contact you to oversee the quality of the study.

Your data may be used in future by the organisations involved in this research for evaluation, teaching and training purposes relating to the provision of NHS care and treatment, and for academic and non-commercial research purposes. If this happens, your personal information will be removed so that no-one will be able to identify you.

Airlie J, *et al. BMJ Open* 2023; 13:e074607. doi: 10.1136/bmjopen-2023-074607

Individuals from BTHFT and regulatory organisations may look at the information we have gathered from you and look at your medical records to check the accuracy of the research study.

Your rights to access, change, or move your information are limited as we need to manage your information in specific ways in order for the research to be reliable and accurate. If you withdraw from the study, we will keep the information about you that we have already obtained. If you take part in study observations and decide to withdraw, we will destroy any observation data collected up to that point, should you request it. To safeguard your rights, we will use the minimum amount of personally identifiable information possible.

You can find out more about how we use your information by contacting the Trial Manager (details below). The research team would be happy to forward any further queries you may have on to the Data Protection Officer.

Will my information be kept confidential?

Yes. If you decide to take part, the information collected about you will be handled strictly in accordance with the consent that you have given and also the Data Protection Act 2018.

You will be allocated a unique study number, which will be used along with your date of birth and initials to identify you instead of your name on all paper and electronic records we create for you.

If you consent to completing study questionnaires, the CTRU will hold a copy of the consent form, which will have your name on it. Your name, address, phone number(s) and email address (if you have one) will also be given to the study team at the CTRU and to authorised members of the research team. This is so that they can contact you about the study when they need to (for example, to send the questionnaires). Information may also be shared with another NHS Provider, for the purposes of data collection and follow-up, including administration of the activPAL.

Your data will be entered onto a secure database held at the CTRU. All access to data and databases will be restricted just to the staff who require access to process and analyse the data.

If you consent to the study observations, a copy of your signed consent form will be held at BTHFT. Data from the observations will be entered onto secure databases held at BTHFT and only accessed by authorised members of the research team.

Will you share any of my information?

We will share identifiable information such as initials, date of birth, gender, NHS Number and post code when requesting information from electronic health records registries (e.g. NHS Digital and the Sentinel Stroke National Audit Programme) to ensure we collect the correct information. This information will include health information, which is regarded as a special category of information. We will use this information to check if you have had any major health events during the course of the study. Information will be securely shared, with these system providers also working in accordance with the Data Protection Act 2018.

It is also possible that the information collected about you may be shared with other research teams to answer new research questions in the future. If this happened, the information would be anonymised so that no-one would be able to identify you from it.

Although the information we collect about you is confidential, should we identify something of concern about your health, or you disclose anything to us, which we feel puts you, or someone else at risk of harm, we may feel it necessary to report this to the appropriate persons. In these circumstances, the researcher would report their concerns to the appropriate clinician in the stroke service or your GP and consider contacting the person responsible for Adult Safeguarding in the NHS Trust, where appropriate. In the event that you were no longer in contact with an NHS stroke service, the researcher would report their concern to the Adult Safeguarding Team in the Social Services for your local area.

How will my information be stored?

BTHFT and the CTRU will be responsible for ensuring that information collected about you is kept safe and secure. Your information will only be accessed by individuals who need to do so in order to do their job. All the data you give us, including identifiable information such as name and date of birth will be securely stored for at least 10 years after the end of the study before being confidentially destroyed.

Airlie J, *et al. BMJ Open* 2023; 13:e074607. doi: 10.1136/bmjopen-2023-074607

Where can I find out more about how my information is used?

You can find out more about how we use your information

- at www.hra.nhs.uk/information-about-patients/
- by contacting the Trial Manager by using the contact details below
- Bradford Teaching Hospitals NHS Foundation Trust Privacy Notice is available to read at https://www.bradfordhospitals.nhs.uk/privacy-statement/

What should I do if I need to update my details?

If you have a change in circumstances (i.e. move house), please contact us using the details below and we will update our records.

For more information please contact:

Dr Seline Ozer – RECREATE Trial Manager

Academic Unit for Ageing and Stroke Research

Bradford Institute for Health Research

Bradford Royal Infirmary

Duckworth Lane

Bradford

BD9 6RJ

Tel: 01274 383908 / 07870 543073

E-mail: recreate@bthft.nhs.uk

Thank you for taking the time to read this information.

FUNDED BY NIHR | National Institute for Health and Care Research
