# Supplementary material for: RECREATE: a study protocol for a multicentre pilot cluster randomised controlled trial (cRCT) in UK stroke services evaluating an intervention to reduce sedentary behaviour in stroke survivors (Get Set Go) with embedded process and economic evaluations
Source: BMJ Open. 2023 Jul 30;13(7):e074607. doi: 10.1136/bmjopen-2023-074607 (PMC10387637; doi:10.1136/bmjopen-2023-074607)
Supplement: Supplementary data [file bmjopen-2023-074607supp004.pdf]

Appendix 4 – Consent form for stroke survivors

Delete this line and add Hospital header

A large-print version of this sheet is available on request

CONSENT FORM FOR STROKE SURVIVORS

COMPLETING QUESTIONNAIRES & WEARING ACTIVITY MONITOR

		Please initial the boxes
1.	I confirm that I have read and understood the Information Sheet Version 4.0 dated 05/08/2022 and the Supplementary Information Sheet, Version 6.0 dated 05/08/2022 for the above study. I have had the opportunity to ask questions and have had these answered satisfactorily.	
2.	I understand that my participation is voluntary and that I am free to withdraw at any time without giving a reason and without my medical care or legal rights being affected.	
3.	I agree for my medical and care records (primary care and hospital records), including my electronic health records to be reviewed by authorised individuals from the study team to obtain data on me.	
4.	I understand that relevant sections of my medical and care records, and data collected during the study, may be looked at by authorised members of the study team, regulatory bodies or sponsor, in order to check that the study is being carried out correctly.	
5.	I agree for my personal details (which may include my initials, date of birth, NHS number, postcode and sex) to be shared with central databases (such as, NHS Digital, Sentinel Stroke National Audit Programme) and the provider of my GP's clinical systems to obtain the electronic data held by my hospital and my GP.	
6.	I understand that my GP will be notified of my participation in this study. I give permission for a copy of this consent form to be sent to my GP.	
7.	I understand that the information collected about me will be used to support other research in the future, and may be shared anonymously with other researchers. I understand that Data Protection regulations will be observed.	
8.	I understand that confidentiality will be maintained unless there are concerns that I, or someone else, is at risk of harm.	

RECREATE ICF – SS Questionnaires Version 4.0 (05/08/2022)
ISRCTN No: 82280581 / REC reference: 19/YH/0403 / IRAS ID: 271111

9.	I agree to take part in the above study and to wear activity monitor(s) as discussed with members of the research team.	
10.	I agree to answer questions about my health and wellbeing.	
11.	I understand that even if I withdraw from the study, the data collected from me up to that point will be used in analysing the results of the study.	
12.	I agree for my details and a copy of this consent form (which will include my name and date of birth) to be stored by the Academic Unit for Ageing & Stroke Research / Clinical Trials Research Unit, University of Leeds for the purposes of this study.	

The following point is OPTIONAL

Even if you agree to take part in this study, you do not have to agree to this statement.

Please tick (✓)

		Yes	No
13.	I am happy to be contacted over the study period by a researcher from Bradford Teaching Hospitals NHS Foundation Trust to discuss taking part in an interview to discuss my experiences of the treatment I have received	☐	☐

PARTICIPANT:

NAME (CAPITALS)	DATE	SIGNATURE

WITNESS (if required):

NAME (CAPITALS)	DATE	SIGNATURE

RESEARCHER:

NAME (CAPITALS)	DATE	SIGNATURE

(1 copy for the participant; 1 copy for the AUASR / CTRU; 1 copy held in patient notes, original stored in Investigator Site File)

For researcher use only

Participant ID (Site no / Trial no)	
Participant Date of Birth	
Participant Initials	

FUNDED BY NIHR | National Institute for Health and Care Research

Airlie J, *et al. BMJ Open* 2023; 13:e074607. doi: 10.1136/bmjopen-2023-074607
