# Supplementary material for: RECREATE: a study protocol for a multicentre pilot cluster randomised controlled trial (cRCT) in UK stroke services evaluating an intervention to reduce sedentary behaviour in stroke survivors (Get Set Go) with embedded process and economic evaluations
Source: BMJ Open. 2023 Jul 30;13(7):e074607. doi: 10.1136/bmjopen-2023-074607 (PMC10387637; doi:10.1136/bmjopen-2023-074607)
Supplement: Author's manuscript [file bmjopen-2023-074607.draft_revisions.pdf]

BMJ Open

BMJ Open is committed to open peer review. As part of this commitment we make the peer review history of every article we publish publicly available.

When an article is published we post the peer reviewers' comments and the authors' responses online. We also post the versions of the paper that were used during peer review. These are the versions that the peer review comments apply to.

The versions of the paper that follow are the versions that were submitted during the peer review process. They are not the versions of record or the final published versions. They should not be cited or distributed as the published version of this manuscript.

BMJ Open is an open access journal and the full, final, typeset and author-corrected version of record of the manuscript is available on our site with no access controls, subscription charges or pay-per-view fees (http://bmjopen.bmj.com).

If you have any questions on BMJ Open's open peer review process please email info.bmjopen@bmj.com

BMJ Open

RECREATE: A study protocol for a multi-centre cluster Randomised Controlled Trial evaluating an intervention to reduce sedentary behaviour in stroke survivors (Get Set Go) with embedded process and economic evaluations

Journal:	*BMJ Open*
Manuscript ID	bmjopen-2023-074607
Article Type:	Protocol
Date Submitted by the Author:	11-Apr-2023

Complete List of Authors:	Airlie, Jennifer; Bradford Institute for Health Research, Academic Unit for Ageing and Stroke Research<br>Burton, Louisa-Jane; Bradford Institute for Health Research, Academic Unit for Ageing and Stroke Research<br>Copsey, Bethan; University of Leeds, Clinical Trials Research Unit (CTRU)<br>English, Coralie; The University of Newcastle, School of Health Sciences; The University of Newcastle Hunter Medical Research Institute, Heart and Stroke Research Program<br>Farrin, Amanda; University of Leeds, Clinical Trials Research Unit (CTRU)<br>Fitzsimons, Claire F.; University of Edinburgh Institute for Sport Physical Education and Health Sciences, Physical Activity for Health and Research Centre<br>Holloway, Ivana; University Medical Center Göttingen, Institute of Medical Psychology and Medical Sociology<br>Horrocks, Judith; University of Leeds, Clinical Trials Research Unit (CTRU)<br>Johansson, Jessica; University of Leeds, Academic Unit for Ageing and Stroke Research<br>Mead, Gillian; University of Edinburgh, Centre for Clinical Brain Sciences<br>Moreau, Lauren; University of Leeds, Clinical Trials Research Unit (CTRU)<br>Ozer, Seline; Bradford Institute for Health Research, Academic Unit for Ageing and Stroke Research<br>Patel, Anita; Anita Patel Health Economics Consulting Ltd<br>Yaziji, Nahel; King's College London, Institute of Psychiatry, Psychology and Neuroscience<br>Forster, Anne; Bradford Institute for Health Research, Academic Unit for Ageing and Stroke Research; University of Leeds, Academic Unit for Ageing and Stroke Research

Keywords:	Clinical Trial, REHABILITATION MEDICINE, Stroke < NEUROLOGY

SCHOLARONE™
Manuscripts

Title: RECREATE: A study protocol for a multi-centre cluster Randomised Controlled Trial evaluating an intervention to reduce sedentary behaviour in stroke survivors (Get Set Go) with embedded process and economic evaluations

Jennifer Airlie[1], Louisa-Jane Burton[1], Bethan Copsey[2], Coralie English[3,4], Amanda Farrin[2], Claire Fitzsimons[5], Ivana Holloway[6], Judith Horrocks[2] , Jessica Faye Johansson[7], Gillian Mead[8], Lauren Moreau[2], Seline Ozer[1], Anita Patel[9], Nahel Yaziji[10], Anne Forster[1,7] on behalf of the RECREATE Programme Management Group

[1.] Academic Unit for Ageing and Stroke Research, Bradford Institute for Health Research, Bradford Teaching Hospitals NHS Foundation Trust, Bradford, UK

[2.] Clinical Trials Research Unit (CTRU), Leeds Institute of Clinical Trials Research, University of Leeds, Leeds, UK

[3.] School of Health Sciences, University of Newcastle, Newcastle, Australia.

[4.] Heart and Stroke Research Program, Hunter Medical Research Institute, Newcastle, Australia

[5.] Physical Activity for Health Research Centre, University of Edinburgh, Edinburgh, UK

[6.] Institute of Medical Psychology and Medical Sociology, University Medical Center Göttingen, Waldweg 37A, 37073, Göttingen, Germany

[7.] Academic Unit for Ageing and Stroke Research, Leeds Institute of Health Sciences, University of Leeds, Leeds, UK

[8.] Centre for Clinical Brain Sciences, University of Edinburgh, Edinburgh, UK

[9.] Anita Patel Health Economics Consulting Ltd, London, UK.

[10.] King's College London, Institute of Psychiatry Psychology and Neuroscience, London, UK.

For peer review only

For peer review only - http://bmjopen.bmj.com/site/about/guidelines.xhtml

Abstract

Introduction: Sedentary behaviour (sitting or lying during waking hours without being otherwise active) is strongly associated with adverse health outcomes, including all-cause, cancer, and cardiovascular mortality in adults. Stroke survivors are consistently reported as being more sedentary than healthy age-matched controls, spending more hours sedentary each day and sustaining longer unbroken bouts of sedentary time. An evidence-based and clinically feasible intervention ('Get Set Go') was developed. A pragmatic definitive trial to evaluate Get Set Go was planned; however, due to the unprecedented effects of the COVID-19 pandemic on National Health Service (NHS) services this study was reduced in size and scope to become an external pilot trial. We report the protocol for the this external pilot trial, which aims to undertake a preliminary exploration of whether Get Set Go is likely to improve the ability to complete extended activities of daily living in the first year after stroke and inform future trial designs in stroke rehabilitation.

Methods and analysis: This study is a pragmatic multi-centre, two-arm, external pilot cluster Randomised Controlled Trial (cRCT) with embedded process and economic evaluations. UK-based stroke services will be randomized 1:1 to the intervention (usual care plus Get Set Go) or control (usual care) arm. Fifteen stroke services will recruit a total of 300-400 stroke in-patient and carer participants, with follow-up at six, 12 and 24 months. The proposed primary endpoint is stroke survivor self-reported Nottingham Extended Activities of Daily Living (NEADL) scale. Endpoint analyses will be exploratory and provide preliminary estimates of intervention effect. The process evaluation will provide valuable information on the intervention fidelity, acceptability, and how it can be optimised.

Ethics and dissemination: Ethical approval has been obtained (Ref: 19/YH/0403). Results will be disseminated through journal publications and conference presentations.

Trial registration: This trial was registered prospectively on 01/04/2020 (ISRCTN ref: 82280581).

For peer review only - http://bmjopen.bmj.com/site/about/guidelines.xhtml
For peer review only

Strengths and limitations of this study

- As well as exploring whether intervention delivery is clinically feasible, this study will provide preliminary evidence as to the impact of the intervention on meaningful outcomes, encompassing short- and long-term changes to activities of daily living, quality of life and objective measures of sedentary behaviour. This will inform data collection processes for future stroke rehabilitation trials.
- The study will generate a novel dataset of objective measures of sedentary behaviour (collected using activity monitors) which to date have been infrequently assessed in post-stroke rehabilitation trials with longer term outcomes up to 24 months.
- The embedded process evaluation (reported separately) will explore intervention fidelity, whether the intervention has its intended effects, and how this aligns with its theoretical underpinnings (resulting in a refined programme theory); the acceptability of the intervention to those delivering and receiving it; and the refinements required to optimise both the intervention implementation and its acceptability.
- The nested Study Within A Trial (SWAT) (reported separately) will systematically evaluate whether a video animation could enhance participant understanding and subsequently uptake, engagement and compliance with the intervention. Information gathered will inform how best to implement the intervention in practice.
- Due to the unprecedented effects of the COVID-19 pandemic on the National Health Service (NHS), the proposed number of participating stroke services and stroke survivor participants will be reduced. As a result, it will not be possible to definitively evaluate the clinical and cost-effectiveness of the Get Set Go intervention

For peer review only

For peer review only - http://bmjopen.bmj.com/site/about/guidelines.xhtml

Introduction

Background and rationale

There are clear associations between sedentary behaviour in the general population and increased risk of adverse health outcomes, including all-cause, cancer, and cardiovascular mortality, and incidence of cardiovascular disease, cancer and Type II diabetes (1, 2). Sedentary behaviour is defined as any waking behaviour characterised by low energy expenditure ≤1.5 Metabolic Equivalent of Task (METs) while in a sitting, lying or reclining posture (3), and importantly differs from physical inactivity, which refers to achieving insufficient levels of moderate to vigorous physical activity (MVPA). An individual may therefore not reach the recommended levels of physical activity yet spend little time sitting, whereas others may be physically active for short bursts (e.g., running for an hour), but spend prolonged periods sitting. Increasing MVPA can offset some of the detrimental effects of sedentary behaviour, although high levels are required (i.e., >300 minutes/ week) (1).

Stroke survivors are more sedentary than other population groups (4-6). Longitudinal studies (7); systematic reviews (8, 9) and observational studies (6) reported that stroke survivors are considerably more sedentary (in hours per day) and have longer unbroken bouts of sedentary time than healthy age-matched controls. Sedentary behaviour does not improve over the first year after stroke (7) and appears independent of the level of functional recovery (7, 8, 10), with physical ability only having a small influence on time spent sitting in people living at home six months after stroke (10). The high amount of reported sitting time by stroke survivors is likely to generate a range of negative outcomes. Epidemiological studies (11) place them in the highest quartile for cardiovascular risk. For a stroke survivor, increased sedentary behaviour may thus result in a "perfect storm" of deteriorating physical function and health-related quality of life, coincident with rising risks for cardiovascular disease.

There has been increasing focus in international public health guidance on reducing sedentary behaviour, particularly for those with disabilities, such as those resulting from stroke (12). Short

For peer review only - http://bmjopen.bmj.com/site/about/guidelines.xhtml
For peer review only

brief activity breaks throughout the day may be as effective as a continuous 30- or 60-minute bout of exercise (summarised in (13)). Experimental studies (primarily short-term, laboratory-based work) provide supporting evidence of the positive effect on metabolic outcomes of breaking up sitting time with short burst of activity in standing (14, 15) and reducing stroke risk (16).

To address the challenge of reducing sedentary behaviour after stroke, we embarked upon a programme of work (RECREATE) to develop an evidence-based and clinically feasible intervention to reduce/ break up sedentary behaviour in stroke survivors. Intervention development was informed by systematic reviews and qualitative work, and underpinned by behaviour change theory (the Behaviour Change Wheel (17)). The final intervention ('Get Set Go') was developed using co-production methods (18). A logic model of intended intervention mechanisms and outcomes was also constructed, and implementation strategies were refined using a case study approach. The intervention is intended to begin early after stroke (in the in-patient setting) and continue into the community, and designed to fit into existing rehabilitation and recovery pathways.

This protocol represents the final stage in the RECREATE programme, which aims to evaluate the developed intervention in a sample of stroke survivors and carers. A large definitive multi-centre cluster Randomised Controlled Trial (cRCT) with 34 stroke services each recruiting 34 participants was initially planned, with the aim of evaluating the effectiveness and cost-effectiveness of the developed intervention. However, due to issues related to the worldwide COVID-19 pandemic, site recruitment was slower than expected. As a result, in Spring 2022, following meetings with the Programme Steering Committee (PSC) and a stakeholder meeting convened by the funder (National Institute for Health and care Research (NIHR)), a decision was taken with the funder to amend the protocol to reduce the trial in size and scope to become an external pilot trial.

Objectives

The primary objective is to undertake a preliminary exploration of whether the intervention (Get Set Go) is likely to improve the ability to complete Extended Activities of Daily Living (EADLs) in the

For peer review only - http://bmjopen.bmj.com/site/about/guidelines.xhtml

first year after stroke. A key secondary objective is to explore whether the intervention is likely to reduce sedentary behaviour. Other secondary objectives are to:

a) Explore core resource use and costs associated with delivering the intervention including impacts on wider care;
b) Explore whether the intervention reduces cardiovascular risk factors;
c) Explore whether the intervention reduces disability;
d) Explore whether the intervention improves health and wellbeing outcomes, such as health related quality of life and mental wellbeing;
e) Explore whether the intervention reduces carer strain;
f) Explore and understand implementation of the intervention, including intervention adherence, compliance, the process, benefits and challenges;
g) Explore and understand how the intervention is experienced and understood by recipients and providers;
h) Explore potential moderators and mediators of the intervention effect.

Objectives a-e will be addressed in the cRCT. Additional objectives addressed in the cRCT relate to gathering feasibility and acceptability data to inform future trial designs in stroke rehabilitation. Objectives f -h will be addressed in an embedded mixed-methods process evaluation (protocol reported separately), which will develop an understanding of how the intervention is implemented, understood and experienced by both providers and recipients.

For peer review only

Methods

Design

This trial is a pragmatic, multicentre, two-arm external pilot cRCT. The Get Set Go intervention will be delivered alongside usual care at the level of the stroke service (i.e., cluster). The cluster design will reduce between-group contamination. Eligible stroke services will be randomised on a 1:1 basis to the two arms of the trial: either intervention (usual care plus Get Set Go) or control (i.e., usual care). All stroke survivors in services allocated to the intervention arm will receive Get Set

For peer review only - http://bmjopen.bmj.com/site/about/guidelines.xhtml

Go; however outcomes will only be measured for those who consent to trial participation.

Settings

The trial will be conducted in up to 15 National Health Service (NHS) stroke services across the United Kingdom (UK). A stroke service is defined as an acute and/ or rehabilitation stroke unit with a linked community service over a defined geographical area. Service eligibility criteria are detailed in Table 1.

Table 1: Stroke service eligibility criteria

**Inclusion criteria**	**Exclusion criteria**
Both the inpatient and community service agree to participate	Previous participation in research leading to the development of the intervention
Availability of research staff to undertake participant recruitment	Currently implementing/ intending to implement similar interventions within trial duration
Agreement that recruitment targets are feasible/ acceptable (considering patient throughput)	Early Supported Discharge (ESD) (or Community if no ESD) waiting list >four weeks post-discharge
The intervention can be feasibly implemented (if so randomised)	

Recruitment

Three to four hundred stroke survivors and carers will be recruited to participate. In line with the trial aims, effectiveness will not be evaluated, therefore no formal power calculation has been conducted, and analyses will be exploratory in nature. Eligibility criteria are outlined in Table 2. Carer involvement is not a requirement for stroke survivor inclusion.

For peer review only - http://bmjopen.bmj.com/site/about/guidelines.xhtml

Table 2: Stroke survivor and carer eligibility criteria

	Inclusion criteria	Exclusion criteria
Stroke survivor	Aged ≥16 years at time of stroke	Receiving palliative care
	Clinical diagnosis of new or recurrent ischaemic or haemorrhagic (excluding subarachnoid haemorrhage) stroke	Due to be discharged outside the defined geographical area of the associated community service(s) participating in the trial.
	Requires manual contact of no more than one person to stand to prevent falling (manual contact consists of continuous or intermittent light touch to assist balance or co-ordination, i.e., not to support body weight)	
	Plan to live in the community post-discharge	
	Informed consent/ consultee declaration is provided	
Carer	Aged ≥16 years	Stroke survivor does not consent to participate
	Family member or friend regularly engaging with a stroke survivor participant (≥once per fortnight)	
	Provide informed consent	

Recruitment of stroke services began in October 2019, and recruitment of stroke survivors and carers started in January 2021. The study is projected to complete recruitment by the end of April 2023.

For peer review only - http://bmjopen.bmj.com/site/about/guidelines.xhtml

Interventions

All stroke survivor participants (irrespective of randomisation allocation) will receive usual care within an organised stroke service. In line with the pragmatic nature of the trial, the comparator is 'usual care' as determined by local policy and practices, to draw comparison with current practice in NHS stroke services, especially given the variation in pathways.

In stroke services allocated to the intervention arm, participants will also receive the Get Set Go intervention. Get Set Go is a whole-service intervention designed to be implemented and embedded within routine practice. Delivery commences in the in-patient stroke unit setting and continues into the community for at least 12 weeks post-discharge. The intervention focuses on:

a) Educating staff and stroke survivors (and their family/ friends/ carers where appropriate) about the importance of standing and moving after stroke;
b) Preparing and enabling staff to support and encourage stroke survivors to stand and move more in everyday stroke care (as part of routine practice);
c) Encouraging stroke survivors to monitor their own standing and moving, with assistance from family/ friends/ carers where appropriate.

As Get Set Go will be delivered at service level, all clinical staff members working within the stroke services randomised to deliver the intervention (i.e., inpatient and community settings) will be invited to attend a training session (~one hour) to prepare for delivering Get Set Go. This will include the intervention rationale and an overview of key components. Staff will participate in practical tasks to ensure they feel confident in supporting and encouraging stroke survivors, who are capable of standing independently/ with the assistance of one person, to stand and move more in everyday stroke care (i.e., as part of routine clinical practice). A Template for Intervention Description and Replication (TIDieR) checklist (19) will be published with trial findings. There are no special criteria for discontinuing or modifying allocated interventions. Recommendations for standing and moving are made by staff as part of delivering the intervention alongside usual care in the stroke unit or community setting, based on their usual assessment techniques and clinical

For peer review only - http://bmjopen.bmj.com/site/about/guidelines.xhtml

judgement. These recommendations will be regularly reviewed and modified in line with stroke survivors' individual capabilities and circumstances.

There are no provisions in place for post-trial care beyond the standard care provided within the participating NHS stroke services.

Study Within a Trial (SWAT)

This cRCT will also incorporate a SWAT (protocol reported separately (20), to evaluate whether a video animation could increase participant understanding of the intervention, and result in improved engagement and compliance, potentially resulting in improved outcomes. Intervention sites will be randomised 1:1 to the SWAT intervention (video animation) or control (usual delivery with no video animation). Results will be reported separately.

Strategies to improve intervention adherence

The implementation team will maintain regular contact with clinical leads in sites allocated to the intervention arm to monitor delivery of the intervention and provide support as required. Clinical staff who are responsible for delivering the intervention will also complete monitoring records for all stroke survivors (whether or not they are trial participants) to document whether key tasks relating to the intervention have been completed (no personal data will be recorded for non-trial participants). It will be advised that a nominated member of staff checks these records have been completed on a regular basis (e.g., towards the end of each shift). Regular review of these records will enable the study team to monitor intervention adherence and provide insight into intervention delivery. A short postal questionnaire completed by stroke survivor trial participants at 12-weeks post-hospital discharge will also assess intervention compliance, with those in the intervention arm responding to questions about the intervention; all participants will be asked about falls and use of a walking aid. Data on compliance will be analysed using descriptive summary statistics by allocation. Finally, intervention fidelity will be explored separately within the process evaluation, through site-based observations of intervention delivery, interviews with those delivering and receiving the intervention, and documentary analysis (including completed intervention materials).

For peer review only

For peer review only - http://bmjopen.bmj.com/site/about/guidelines.xhtml

Endpoints

The primary endpoint is stroke survivor self-reported Extended Activities of Daily Living (EADLs) at 12 months post-registration. The key secondary endpoint is mean daily sedentary time (minutes) at 12 months post-registration using activity monitor (ActivPAL) data, which provides a valid measure of posture and transitions in people with impaired mobility (21). Other secondary endpoints at six, 12- and 24- months post-registration (unless stated otherwise) for stroke survivor participants are listed below:

- Sedentary behaviour measured by activity monitor;
- EADLs (at six and 24 months only) measured by Nottingham Extended Activities of Daily Living scale (NEADL) (22);
- Health and disability measured by World Health Organisation Disability Assessment Schedule 2.0 (12-item; WHODAS 2.0-short) (23-27);
- Mental well-being measured by Warwick-Edinburgh Mental Well-being Scale (WEMWBS) (28);
- Health status measured by European Quality of Life 5-Dimension Health Questionnaire (5 levels; EQ-5D-5L) (29);
- Quality-Adjusted Life Year (QALY) gains (as above);
- Fatigue as measured by Fatigue Assessment Scale (FAS) (30, 31);
- Death;
- Falls and use of a walking aid (12-weeks post-discharge, and at six, 12 and 24 months);
- Self-reported sedentary behaviour measured by the Measure of Older Adults' Sedentary Time (MOST) (32) and a Sedentary Behaviour Visual Analogue Scale (adapted from (33)).
- Institutionalisation, hospital re-admission, and emergency department attendance rates;
- Cardiovascular risk markers: Body Mass Index (BMI), waist circumference, blood pressure (BP)
- Total major vascular events: Composite measure of non-fatal stroke, non-fatal myocardial infarction or death due to any vascular cause (including unexplained sudden death) (34);

For peer review only

For peer review only - http://bmjopen.bmj.com/site/about/guidelines.xhtml

- Self-reported health and social care service use and informal care inputs measured by a Client Services Receipt Inventory (CSRI) specifically adapted for this trial from versions used in previous stroke rehabilitation trials (35, 36).
- Costs from a health and social care perspective, and a societal perspective that further includes informal care.

The carer end-point is caregiver burden measured by Modified Caregiver Strain Index (MCSI (37)) at six, 12 and 24 months.

An overview of which assessments are undertaken at each time-point and the method of completion is provided in the Table 3. Information about the validity and reliability of the measures used is available in Appendix 1. Stroke survivors will also complete an additional brief postal questionnaire at 12-weeks post-discharge.

For peer review only

For peer review only - http://bmjopen.bmj.com/site/about/guidelines.xhtml

Table 3: Overview of assessments and method of completion at each study time-point

**Outcome / Assessment**	**Method of completion**	**Time-point**			
		**Baseline**	**6 months post-registration**	**12 months post-registration**	**24 months post-registration**
**Stroke Survivor**					
Demographic Details	Self-report	X			
Nottingham Extended Activities of Daily Living Scale (NEADL) (22)	Self-report	X	X	X	X
World Health Organisation Disability Assessment Scale (WHODAS) 2.0 12 Item (23-27)	Self-report	X	X	X	X
Warwick-Edinburgh Mental Well-being Scale (WEMWBS) (28)	Self-report	X	X	X	X
European Quality of Life 5-Dimension Health Questionnaire (EQ-5D-5L) (29)	Self-report	X	X	X	X
Fatigue Assessment Scale (FAS) (30, 31)	Self-report	X	X	X	X
Measure of Older Adults' Sedentary Time (MOST) (32)	Self-report	X	X	X	X
Sedentary Behaviour Visual Analogue Scale (adapted from	Self-report	X	X	X	X

For peer review only

For peer review only - http://bmjopen.bmj.com/site/about/guidelines.xhtml

(33))					
Use of a walking aid	Self-report	X	X	X	X
Falls question	Self-report	X	X	X	X
Hospital Admissions	Self-report / electronic health records		X	X	X
Client service receipt inventories (CSRI) to collect data on health and social care use and informal care inputs (adapted from (35, 36)	Self-report	X	X	X	X
Total major vascular events (34)	Electronic health records via routine data providers	X	X	X	X
Sedentary behaviour – activPAL measurements	Researcher administration / post	X	X	X	X
Risk markers for cardiovascular disease (Height* and weight for Body Mass Index (BMI); waist circumference; blood pressure (BP)) *collected at baseline only	Researcher collection from notes/ Researcher visit/ self-completion	X	X	X	X
Adverse Events (AE)	Self-report / Electronic		X	X	X

For peer review only - http://bmjopen.bmj.com/site/about/guidelines.xhtml
For peer review only

	health records				
Survival status			X	X	X
Admission to institution	Self-report		X	X	X
**Carer**					
Demographic Details	Self-report	X	(X)	(X)	(X)
Modified Caregiver Strain Index (MCSI) (37)	Self-report		X	X	X
Employment/ occupation status	Self-report	X	X	X	X

(X) only collected if 'new carer'

Randomisation and blinding

Site randomisation will be performed centrally by the Clinical Trials Research Unit (CTRU) and the allocation sequence will be computer-generated using a minimisation programme incorporating a random element to ensure the treatment arms are well-balanced. Eligible stroke services will be randomised on a 1:1 basis either to intervention or control, stratified by:

1. Sentinel Stroke National Audit Programme (SSNAP) grading level (38): A vs B/C/D/E (based on the latest available audit data at the time of randomisation: A=First class service, B=Good/ excellent in many aspects, C=Reasonable overall, some areas require improvement, D=Several areas require improvement, E=Substantial improvement required).

2. Single or combination of trusts: Single or combination.

3. Stroke unit size (acute): Median cut-off of ≤24 and >24 beds based on SSNAP acute organisational audit data (type 2 (solely for patients >72 hours post-stroke) and type 3 beds (used for both pre- and post-72-hour care) combined – based on the SSNAP audit data and confirmation by site).

For peer review only - http://bmjopen.bmj.com/site/about/guidelines.xhtml

Concealment of sequencing is ensured by the physically separate locations of the CTRU (where the randomisation outcome is stored) and the blinded study researchers. Following the randomisation, unblinded members of the study team, the Principal Investigator at the site, and clinical staff who will be involved in intervention delivery, will be informed of the randomisation outcome via secure email to facilitate organisation of intervention training in services allocated to this arm. Local researchers responsible for participant recruitment will be notified that randomisation has taken place but will remain blinded to randomisation outcome and will operate independently of the clinical staff involved in intervention delivery.

Local researchers involved in participant recruitment and in-person data collection (including at baseline, administering activity monitors and assessment of cardiovascular risk markers) will be blinded to the treatment allocation for their site. At the follow-up time-points, participant-completed outcome measures will be administered by CTRU staff via postal questionnaire, eliminating the potential for interviewer bias. The activity monitor (upon which data is recorded and stored until download) is sealed with no external indication of recording therefore it is not open to bias. Finally, data on mortality, re-admissions, and use of institutional care will be collected from independent sources and are unlikely to be subject to bias. Instances of unblinding will be recorded via staff self-report.

Participants, clinical staff involved in intervention delivery, and the statistical team analysing the outcome data, will not be blinded to treatment allocation. However, to minimise treatment bias, clinical staff involved in intervention delivery will not be overtly informed of which patients are participating in the trial. Additionally, to monitor for potential selection bias, key characteristics of included trial participants will be compared with publicly available datasets (e.g., SSNAP (38)) to monitor for evidence of selection bias.

Procedure

Eligible stroke survivors will be initially approached whilst they are an in-patient by a local researcher, who will provide information about the trial before seeking informed consent. Should

For peer review only - http://bmjopen.bmj.com/site/about/guidelines.xhtml

stroke survivors be deemed to lack capacity to provide informed consent, the researcher will seek to identify ‘a supporting family member, friend or carer’ (defined as someone who is in regular (at least weekly) contact with the stroke survivor), to support intervention delivery and outcome assessment. This individual will not necessarily be enrolled in the study themselves, unless they choose to enrol as a carer participant. If such a supporter is identified, the local researcher will either: act in accordance with an advance directive relative to research participation (if available) or seek advice from a personal consultee (through Consultee declaration).

Carer participants will be approached by the local researcher once stroke survivors with capacity have given their verbal agreement or following provision of a consultee declaration for a stroke survivor participant who lacks capacity. Those wishing to participate will be asked to provide informed consent using the same procedure described above. Carers lacking the capacity to provide written informed consent will be excluded.

The local researcher will administer baseline questionnaires to stroke survivor and carer participants face-to-face and offer support where required. Proxy completion of questionnaires by a carer, family member or friend will be permitted (for those with and without capacity), with separate proxy questionnaires provided if required. Stroke survivors will also be asked to continuously wear an activPAL activity monitor on the thigh of their strongest/ unaffected leg for nine consecutive days, The monitor will be attached by the local researcher. They will also be asked to complete a purposely developed daily diary. This will capture if/ when the monitor has been removed; the time they went to bed and got up each day and whether they had episodes of sleep (i.e., naps) during the day. This information will assist with analysis and facilitate monitoring of adverse reactions to the materials used to attach the monitor. Local researchers will also collect data on risk markers for cardiovascular disease through face-to-face assessment or retrieval from medical records. These include height and weight (for calculation of BMI), waist circumference and blood pressure.

At the follow-up time points (six, 12- and 24-months post-registration), participant-completed outcome assessments will be administered by post in an assessment pack, including a covering

For peer review only - http://bmjopen.bmj.com/site/about/guidelines.xhtml

letter and a pre-paid envelope for postal return. Activity monitors may be administered face-to-face by a local researcher or alternatively, be sent by post to the participant and returned from stroke survivor participants via pre-paid envelope. Cardiovascular risk markers (with the exception of height) will be collected alongside the activity monitor assessment. If it is not possible to organise a face-to-face visit, with the participants' agreement, options for self-assessment will be explored. Guidance will be provided to local researchers as to the action required if a blood pressure reading is abnormal, including reporting this to a named clinician or notifying the participant's General Practitioner (GP). The participant will also be encouraged to discuss this with their GP.

At each participant follow-up, changes in circumstances, including change of address, institutionalisation, and death will be obtained from either recruiting teams, healthcare providers, participants, or their relatives. Additionally, for stroke survivors, the following data will be collected from electronic health records via routine data providers or directly from the recruiting hospital at each time-point: total major vascular events (composite of non-fatal stroke; non-fatal myocardial infarction; or death due to any vascular cause (including unexplained sudden death) (34); death; hospital admissions; emergency department attendance and institutionalisation. Stroke survivors will be made aware of the use of routine data providers (including NHS England, SystmOne, EMISWeb, and other relevant providers) at the time of study entry.

The study timeline for participants is presented in Figure 1.

[Figure 1 about here]

Initial letters, reminder letters and a participant pathway diagram will be distributed to participants to maximise data return at all time-points. The questionnaire sent to assess intervention compliance at 12-weeks post-hospital discharge will also serve as a reminder of study participation and act as a retention tool. At the follow-up time-points, reminders will be administered via telephone, text message, email, or post (according to participant preference) should the questionnaire not be returned within two weeks. If outcome assessments (with particular emphasis on the NEADL as the primary outcome), cannot be obtained by post, then (where appropriate) the

For peer review only - http://bmjopen.bmj.com/site/about/guidelines.xhtml

For peer review only

assessments may be administered over the telephone or face-to-face to maximise return of data. Missing data, except individual data items collected via the postal assessment packs, will be sought until: it is received; confirmed as not available; or the trial is at analysis.

In instances where a participant withdraws from the trial clarification will be sought as to whether this is from all trial activities or individual components, e.g., wearing the activity monitor or consenting to be contacted about participation in process evaluation activities. Unless participants explicitly instruct otherwise, electronic health record data will continue to be collected.

Data management

Data will be monitored for quality and completeness using established verification, validation and checking processes, e.g., double data entry, and securely stored. Received activPAL data will be subject to quality checks by researchers and where needed, additional details to aid interpretation will be sought from the recruiting site and/ or participant diaries. All data collected during the course of the trial will be kept strictly confidential using established processes. Data will be securely archived for ten years after the completion of the study and then destroyed.

Statistical analysis

All analyses and data summaries will be conducted on the intention-to-treat population (ITT) (39), defined as all participants registered to the trial, regardless of non-compliance with the protocol or withdrawal from the study or losses to follow-up. All participants recruited within a stroke service will be analysed according to the randomised allocation for that stroke service. No formal interim or sub-group analyses are planned, and final analysis will take place when all available data have been received.

Primary endpoint analysis

In line with study objectives, all endpoint analyses will be exploratory and provide preliminary estimates of intervention effect. The primary outcome will be compared between randomised groups using multi-level mixed effects model (40) adjusting for design effects and other relevant

For peer review only - http://bmjopen.bmj.com/site/about/guidelines.xhtml

known predictors of outcome, with participants nested within services, with services treated as random effects. If appropriate, missing data will be multiply imputed at the individual participant level. Parameter estimates will be reported with 95% confidence intervals. Sensitivity analyses of the primary endpoint will be conducted to assess the impact of missing data, the choice of imputation model and of assuming data are missing not at random, as appropriate. For the primary analysis, missing data will be assumed missing at random.

Secondary endpoints analyses

Secondary outcomes will be analysed in a similar manner to the primary outcome. For secondary outcomes, summary statistics will be presented for each time point by treatment group (means, standard deviations, medians, minimum, maximum, and quartiles for continuous variables, and counts and percentages for categorical variables). Secondary outcomes will be analysed using the same approach as the primary outcome with the relevant model for the type of outcome variable using multi-level linear or logistic regression, with multiple imputation for missing data where appropriate. Continuous distributions will be transformed where residuals are non-normal.

Self-reported falls, institutionalisation, re-admission, cardiovascular risk markers, total major vascular events and death rates between treatment arms will be summarised descriptively.

Quantitative summaries of intervention delivery will evaluate uptake of the intervention, adherence to the processes, staff and participant engagement and quality of intervention delivery, overall and by each site. Data on usual care will be summarised descriptively.

Activity monitor (activPAL) data

Sedentary time, as measured by activPAL activity monitors, will be analysed as total sedentary minutes on an average day as well as the percentage of sedentary time during wake time. Data from the monitors will be automatically recorded and processed using the activPAL software (PAL Technologies Ltd.). We will process data, remove sleep time and calculate, for each day, the total sedentary time; total sitting time accumulated in bouts ≥30 minutes, and bouts ≥ 60 minutes, number of sedentary bouts ≥30 minutes and bouts ≥ 60 minutes, total standing time, total stepping

For peer review only

For peer review only - http://bmjopen.bmj.com/site/about/guidelines.xhtml

time and number of sit-to-stand transitions.

We will then calculate the key secondary outcome of mean daily sedentary time for the days when the monitor was worn. All other sedentary behaviour secondary outcomes will be calculated in the same way. The sedentary behaviour outcomes would ideally be based on seven days of continuous wear of the monitor. The calculation of sleep time, definition of a valid wear day and number of valid wear days required for inclusion in the analysis will be informed by the results of earlier feasibility work.

Further secondary analyses

Potential moderator variables: Potential predictors of response to the intervention will be explored via inclusion of key baseline stroke survivor characteristics (identified in our earlier work) as interaction effects in the primary analysis model, to understand who might benefit from the intervention.

Potential mediator variables will be explored by modelling the relationship between process variables (e.g., intervention adherence, number/ frequency of prompts and visits from staff, participant self-reported use of the guide, mediators such as fatigue or mood, etc.) and the primary outcome.

Methods in analysis to handle protocol non-adherence and any statistical methods to handle missing data

All analyses and data summaries will be conducted on the ITT population. Missing data is anticipated at the item, scale and assessment levels. Missing data on key variables/ questionnaires will be explored. Missing data will be multiply imputed, where appropriate. Reasons for missing participant assessments will be explored and considered in the imputation process (e.g., died, moved away, etc.). If someone dies, they will be classed as lost to follow-up. When individual scales within the assessment questionnaire have missing items, scoring instructions specific to the scale will be followed, or if no such instructions exist, the summary scores will be

For peer review only

For peer review only - http://bmjopen.bmj.com/site/about/guidelines.xhtml

calculated from the non-missing items using multiple imputation under the missing at random (MAR) mechanism, as long as at least 75% items are non-missing. We will test missing data assumptions in a sensitivity analysis.

Health economic analysis

The embedded health economic evaluation will assess resource use, costs and outcomes to inform data collection approaches for a future definitive trial, specifically:

- Alternative approaches to measuring hospital resource use (routine records versus self-report);
- A short (6-month) versus medium (12-month) versus longer (24-month) analytical time horizon; and
- A reduced list (narrow perspective) evaluation based on routine data versus long list (comprehensive perspective) evaluation incorporating self-reported data.

Two perspectives will be considered, as relevant to the analyses: health/social care (including institutionalisation), and a wider perspective covering health/social care (including institutionalisation) plus informal care given the significance of such inputs for this patient group.

Resource use data will be collected using a combination of NHS England secondary care data and self-report approaches via the CSRI. Resource use will be combined with relevant unit costs, using within-programme estimates related to the intervention, national estimates for other health and social care resources, and both an opportunity cost and replacement cost approach in turn for informal care. Total costs will be computed for each individual according to each of the estimation alternatives set out above. Differences between the two trial arms will be compared using bootstrap regression methods due to the expected skewness in data distributions.

Exploratory cost-effectiveness analyses will combine total costs with the primary outcome and with QALY gains estimated by attaching relevant general population utility weights to EQ-5D-5L health

For peer review only

For peer review only - http://bmjopen.bmj.com/site/about/guidelines.xhtml

states at each time point. Uncertainty will be analysed using cost-effectiveness planes and cost-effectiveness acceptability curves (CEACs). Sensitivity analyses will explore the potential impact on cost-effectiveness from different implementation/costing scenarios. This would better inform future trial design and implementation discussions. Other relevant sensitivity analyses will be determined during the study and will be specified in the analysis plan prior to analyses.

The economic analyses will follow the same broad principles as the outcomes analyses, e.g. use of a signed off detailed analysis plan prior to analysis, adherence to ITT principles, imputation to account for missing values as appropriate, inclusion of baseline and other covariates for the comparisons, accounting for the clustering of patients within stroke services.

Oversight and monitoring

This trial is the final study in a seven-year Programme Grant for Applied Research funded by the NIHR. As such, a Programme Management Group (PMG), chaired by the Chief Investigator (CI) and attended by the programme co-applicants meets at least every four months, for strategic oversight and management of the programme, and to monitor progress against key objectives. An independent Programme Steering Committee (PSC) chaired and attended by independent experts meets as a minimum every 12 months, and will provide external guidance and monitor progress. Finally, a Trial Management Group (TMG) comprised of the CI, key co-applicants, study researchers and key members of the project delivery team at the CTRU will be convened to meet every month to oversee day-to-day management of the trial. A Data Monitoring Committee is not required due to the lack of serious adverse events anticipated to be due to the intervention. The PSC will take on the role of monitoring safety concerns.

Adverse event reporting and harms

Adverse Events (AEs) such as falls and musculoskeletal injuries represent an inherent consequence of an active rehabilitation process and therefore cannot be entirely avoided. Similarly, in this patient population, acute illness resulting in hospitalisation, new medical problems and deterioration of existing medical problems are expected. In recognition of this, events fulfilling

For peer review only

For peer review only - http://bmjopen.bmj.com/site/about/guidelines.xhtml

the definition of an AE or serious adverse events (SAE) will not be reportable in this study unless they result from administration of any research procedures and therefore fulfil the definition of an Unexpected and Related Serious AE, or if they constitute one of the following: death, hospital readmission, institutionalisation, treatment on an emergency out-patient basis or emergency department attendance. The latter will be reported at six-, 12- and 24- months post-registration follow-up by the clinical research team using health and social care records. All Related/ Unexpected Serious AEs occurring from the date of consent up to twenty-four months post registration must be reported within 24 hours of the clinical research staff becoming aware of the event. All Related/ Unexpected Serious AEs will be reviewed by the CI and subject to expedited reporting to the Sponsor and the main REC by the CTRU on behalf of the CI within 15 days.

Patient and Public Involvement (PPI)

PPI is central to this research. The research question was informed by initial discussion with our stroke survivor and carer group, who highlighted the need for interventions to encourage active movement after stroke and contributed to grant development. PPI is subsequently being sought at every stage of the programme of research. Specific input from our PPI representatives includes: being actively involved in the design and management (e.g. steering/advisory group) of this research; assisting with developing participant information resources (including trial documents and intervention materials); contributing to ongoing reporting of the study and dissemination of emerging research findings.

Ethics and dissemination

The current protocol (v9.0 10/08/22) received ethical approval from the Yorkshire & The Humber – Bradford-Leeds REC (Ref: 19/YH/0403). Protocol amendments are processed in line with Health Research Authority and REC guidelines and processes. The results of the study will be published in peer-reviewed publications and will be presented at relevant national and international conferences. Authorship will be agreed in accordance with International Committee of Medical Journal Editors recommendations and the publication policy that has been agreed by the PMG for

For peer review only - http://bmjopen.bmj.com/site/about/guidelines.xhtml

this programme of research. In collaboration with patient and public involvement representatives, lay reports to disseminate research findings to patient groups and clinical staff at participating sites will also be compiled.

For peer review only

For peer review only - http://bmjopen.bmj.com/site/about/guidelines.xhtml

References

1. Dempsey PC, Biddle SJ, Buman MP, et al. New global guidelines on sedentary behaviour and health for adults: broadening the behavioural targets. International Journal of Behavioral Nutrition and Physical Activity. 2020;17(1):1-12.

2. U.S. Department of Health and Human Services. Physical Activity Guidelines for Americans, 2nd edition. Washington, DC: U.S. Department of Health and Human Services; 2018.

3. Tremblay MS, Aubert S, Barnes JD, et al. Sedentary behavior research network (SBRN)–terminology consensus project process and outcome. International journal of behavioral nutrition and physical activity. 2017;14:1-17.

4. Butler EN, Evenson KR. Prevalence of physical activity and sedentary behavior among stroke survivors in the United States. Topics in stroke rehabilitation. 2014;21(3):246-55.

5. Paul L, Brewster S, Wyke S, et al. Physical activity profiles and sedentary behaviour in people following stroke: a cross-sectional study. Disability and rehabilitation. 2016;38(4):362-7.

6. English C, Healy GN, Coates A, et al. Sitting and activity time in people with stroke. Physical therapy. 2016;96(2):193-201.

7. Tieges Z, Mead G, Allerhand M, et al. Sedentary behavior in the first year after stroke: a longitudinal cohort study with objective measures. Archives of physical medicine and rehabilitation. 2015;96(1):15-23.

8. English C, Manns PJ, Tucak C, et al. Physical activity and sedentary behaviors in people with stroke living in the community: a systematic review. Physical therapy. 2014;94(2):185-96.

9. Galea SL, Lee M-J, English C, et al. Sedentary versus active behavior in people after stroke. Physical Therapy Reviews. 2015;20(1):1-7.

10. English C, Healy GN, Coates A, et al. Sitting time and physical activity after stroke: physical ability is only part of the story. Topics in stroke rehabilitation. 2016;23(1):36-42.

11. Wullems JA, Verschueren SM, Degens H, et al. A review of the assessment and prevalence of sedentarism in older adults, its physiology/health impact and non-exercise mobility counter-measures. Biogerontology. 2016;17:547-65.

For peer review only

For peer review only - http://bmjopen.bmj.com/site/about/guidelines.xhtml

12. World Health Organization. WHO guidelines on physical activity and sedentary behaviour. 2020.

13. Dempsey PC, Owen N, Biddle SJ, et al. Managing sedentary behavior to reduce the risk of diabetes and cardiovascular disease. Current diabetes reports. 2014;14:1-11.

14. Benatti FB, Ried-Larsen M. The effects of breaking up prolonged sitting time: a review of experimental studies. Medicine & Science in Sports & Exercise. 2015;47(10):2053-61.

15. English C, Janssen H, Crowfoot G, et al. Frequent, short bouts of light-intensity exercises while standing decreases systolic blood pressure: Breaking Up Sitting Time after Stroke (BUST-Stroke) trial. International journal of stroke. 2018;13(9):932-40.

16. Mackie P, Weerasekara I, Crowfoot G, et al. What is the effect of interrupting prolonged sitting with frequent bouts of physical activity or standing on first or recurrent stroke risk factors? A scoping review. PloS one. 2019;14(6):e0217981.

17. Michie S, Van Stralen MM, West R. The behaviour change wheel: a new method for characterising and designing behaviour change interventions. Implementation science. 2011;6(1):1-12.

18. Hall J, Morton S, Hall J, et al. A co-production approach guided by the behaviour change wheel to develop an intervention for reducing sedentary behaviour after stroke. Pilot and Feasibility Studies. 2020;6(1):1-13.

19. Hoffmann TC, Glasziou PP, Boutron I, et al. Better reporting of interventions: template for intervention description and replication (TIDieR) checklist and guide. BMJ. 2014;348.

20. Ahmed S, Airlie J, Clegg A, et al. A new opportunity for enhancing trial efficiency: Can we investigate intervention implementation processes within trials using SWAT (study within a trial) methodology? Research Methods in Medicine & Health Sciences. 2022;3(3):66-73.

21. Taraldsen K, Askim T, Sletvold O, et al. Evaluation of a body-worn sensor system to measure physical activity in older people with impaired function. Physical therapy. 2011;91(2):277-85.

22. Nouri F, Lincoln N. An extended activities of daily living scale for stroke patients. Clinical rehabilitation. 1987;1(4):301-5.

For peer review only

For peer review only - http://bmjopen.bmj.com/site/about/guidelines.xhtml

23. Üstün TB, Chatterji S, Kostanjsek N, et al. Developing the World Health Organization disability assessment schedule 2.0. Bulletin of the World Health Organization. 2010;88:815-23.

24. Schlote A, Richter M, Wunderlich M, et al. WHODAS II with people after stroke and their relatives. Disability and rehabilitation. 2009;31(11):855-64.

25. Garin O, Ayuso-Mateos JL, Almansa J, et al. Validation of the "World Health Organization Disability Assessment Schedule, WHODAS-2" in patients with chronic diseases. Health and quality of life outcomes. 2010;8:1-15.

26. Küçükdeveci AA, Kutlay Ş, Yıldızlar D, et al. The reliability and validity of the World Health Organization Disability Assessment Schedule (WHODAS-II) in stroke. Disability and rehabilitation. 2013;35(3):214-20.

27. Hirve S, Juvekar S, Sambhudas S, et al. Does self-rated health predict death in adults aged 50 years and above in India? Evidence from a rural population under health and demographic surveillance. International journal of epidemiology. 2012;41(6):1719-27.

28. Tennant R, Hiller L, Fishwick R, et al. The Warwick-Edinburgh mental well-being scale (WEMWBS): development and UK validation. Health and Quality of life Outcomes. 2007;5(1):1-13.

29. Herdman M, Gudex C, Lloyd A, et al. Development and preliminary testing of the new five-level version of EQ-5D (EQ-5D-5L). Quality of life research. 2011;20:1727-36.

30. Mead G, Lynch J, Greig C, et al. Evaluation of fatigue scales in stroke patients. Stroke. 2007;38(7):2090-5.

31. Michielsen HJ, De Vries J, Van Heck GL. Psychometric qualities of a brief self-rated fatigue measure: The Fatigue Assessment Scale. Journal of psychosomatic research. 2003;54(4):345-52.

32. Gardiner PA, Eakin EG, Healy GN, et al. Feasibility of reducing older adults' sedentary time. American journal of preventive medicine. 2011;41(2):174-7.

33. Chastin SF, Dontje ML, Skelton DA, et al. Systematic comparative validation of self-report measures of sedentary time against an objective measure of postural sitting (activPAL). International Journal of Behavioral Nutrition and Physical Activity. 2018;15(1):1-12.

For peer review only

For peer review only - http://bmjopen.bmj.com/site/about/guidelines.xhtml

34. PROGRESS Collaborative Group. Randomised trial of a perindopril-based blood-pressure-lowering regimen among 6105 individuals with previous stroke or transient ischaemic attack. The Lancet. 2001;358(9287):1033-41.

35. Forster A, Dickerson J, Young J, et al. A structured training programme for caregivers of inpatients after stroke (TRACS): a cluster randomised controlled trial and cost-effectiveness analysis. The Lancet. 2013;382(9910):2069-76.

36. Forster A, Ozer S, Crocker TF, et al. Longer-term health and social care strategies for stroke survivors and their carers: the LoTS2Care research programme including cluster feasibility RCT. Programme Grants Appl Res 2021;9(3).

37. Thornton M, Travis SS. Analysis of the reliability of the modified caregiver strain index. The Journals of Gerontology Series B: Psychological Sciences and Social Sciences. 2003;58(2):S127-S32.

38. Sentinel Stroke National Audit Programme (SSNAP). Annual Public Report National Results2019. Available from: https://www.strokeaudit.org/Documents/National/Clinical/Apr2017Mar2018/Apr2017Mar2018-AnnualReport.aspx (Accessed 13 March 2023).

39. Altman DG. Practical statistics for medical research. Boca Raton, Florida: Chapman & Hall/CRC; 1999.

40. Hayes RJ, Moulton LH. Cluster randomised trials, 2nd Ed. Boca Raton: CRC Press; 2017.

For peer review only

For peer review only - http://bmjopen.bmj.com/site/about/guidelines.xhtml

Authors' contributions: AF is lead grant holder and Chief Investigator, and will oversee the design and implementation of the trial. CE, CF and GM, are coinvestigators who have contributed to development of the protocol, and attend regular programme meetings, providing advice as needed. AFa, assisted by IH and BC, led the statistical and methodological design of the trial and is the statistical guarantor responsible for the main statistical analysis and reporting. AP leads the design of the health economic components of the trial and is responsible for the health economic analyses, assisted by NY. LM, assisted by JH, leads on data management and supports the day-to-day operation of the trial. SO, assisted by JA, is responsible for managing implementation of the intervention and delivery of the trial. LJB also assisted with managing delivery of the trial. JFJ leads the embedded process evaluation. JA leads on activPAL data implementation and data issues. JA and LJB drafted the manuscript. All authors read and approved the final manuscript. This paper is written on behalf of the RECREATE Programme Management Group who meet regularly to oversee the programme of research. All authors reviewed and approved the final manuscript.

Acknowledgements: The authors would like to acknowledge the support of the Clinical Research Network (CRN) and also express our appreciation for all of the hard work and support provided by the clinical teams who are helping to deliver this trial. The authors would also like to thank members of the Programme Steering Committee for their helpful contributions through the RECREATE programme of research.

Finally the authors would like to acknowledge the expert support of the RECREATE Programme Group who meet regularly to oversee the programme of research. The group is comprised of: Jennifer Airlie, Karen Birch, Gillian Carter, Bethan Copsey, Florence Day, Coralie English, Amanda Farrin, Alison Fergusson, Claire Fitzsimons, Anne Forster, Jessica Faye Johansson, Rebecca Lawton, Laura Marsden, Gillian Mead, Lauren Moreau, Seline Ozer, Anita Patel, Rosemary Shannon, Nahel Yaziji.

For peer review only

For peer review only - http://bmjopen.bmj.com/site/about/guidelines.xhtml

Funding statement: This work was supported by the National Institute for Health and Care Research (NIHR) under its Programme Grants for Applied Research Programme, grant number RP-PG-0615-20019). The views expressed are those of the author(s) and not necessarily those of the NIHR or the Department of Health and Social Care. The Funder has had no role in trial design, beyond setting the research question the trial addresses. Data collection, management, analysis and interpretation will remain independent of the Funder.

Sponsor: The trial sponsor is Bradford Teaching Hospitals NHS Foundation Trust, Research Management & Support Office, Bradford Institute for Health Research, Bradford, BD9 6RJ, United Kingdom. Tel: +44 (0)1274 38 2575; e-mail: jane.dennison@bthft.nhs.uk

The Sponsor will maintain oversight of trial processes, but is not involved in trial design or delivery processes. The Sponsor will not participate in data analysis or trial reporting processes.

Data sharing statement: The quantitative dataset will be maintained by the CTRU, with direct access provided to the CI, study researchers and to the necessary authorities, in order to enable monitoring of the data for audit. Any data requests should be sent to the corresponding author and would be subject to review by a subgroup of the trial team, which will include the data guarantor, Professor Farrin. All data-sharing activities would require a data-sharing agreement.

Competing interests statement:

AF, AFa, CE, CF, IH, GM and AP are coinvestigators on the grant funding this work therefore are partially supported by the National Institute for Health and Social Care Research (NIHR) (grant number RP-PG-0615-20019).

AF has received additional research grant support from NIHR through the following funding streams: Senior Investigator award, Health Technology Assessment (HTA) and Health and Social Care Delivery Research (HS&DR). AF has previously received support from the Stroke Association to attend the UK stroke forum and received payment from the National Institute for Health (USA)

For peer review only - http://bmjopen.bmj.com/site/about/guidelines.xhtml

for panel membership (2021, 2022). AF is currently the chair / a member of programme steering committees for NIHR research programmes (Grant reference numbers: NIHR 202339, NIHR 202020) and has served on the following panels: NIHR Doctoral Fellowships, NIHR senior investigators committee (2019/20), NIHR HS&DR committee (2016-2018) and Stroke Association Funding.

CE has received grant funding from the Netherlands Organisation for Scientific Research (NOW) Taskforce for Applied Research (SIA RAAK) for work in a similar area (i.e. sitting less and moving more after stroke) and is a Non-executive Director representing interests of Research and Chair of Research Advisory Committee for the Stroke Foundation of Australia (unpaid).

CF is a coinvestigator / collaborator on other grants on the topic of sedentary behaviour / physical activity and is therefore partially supported by grant funding received from the University of Edinburgh and the Irish Health Board. CE has previously been supported to conduct work in a similar area by grant funding received from the Chief Scientist Office of the Scottish Government, Medical Research Council (MRC) Public Health Intervention Development (PHIND) award and the University of Edinburgh.

JA, LJB, BC, JH, JFJ, LM, SO and NY report no competing interests related to the manuscript.

Patient and public involvement: Patients and/or the public were involved in the design, conduct, reporting and dissemination plans of this research. Please refer to the methods section for further details.

Twitter: @RECREATE_stroke

Figure 1 legend: Study timeline for participants

For peer review only

For peer review only - http://bmjopen.bmj.com/site/about/guidelines.xhtml

Figure 1: Study timeline for participants

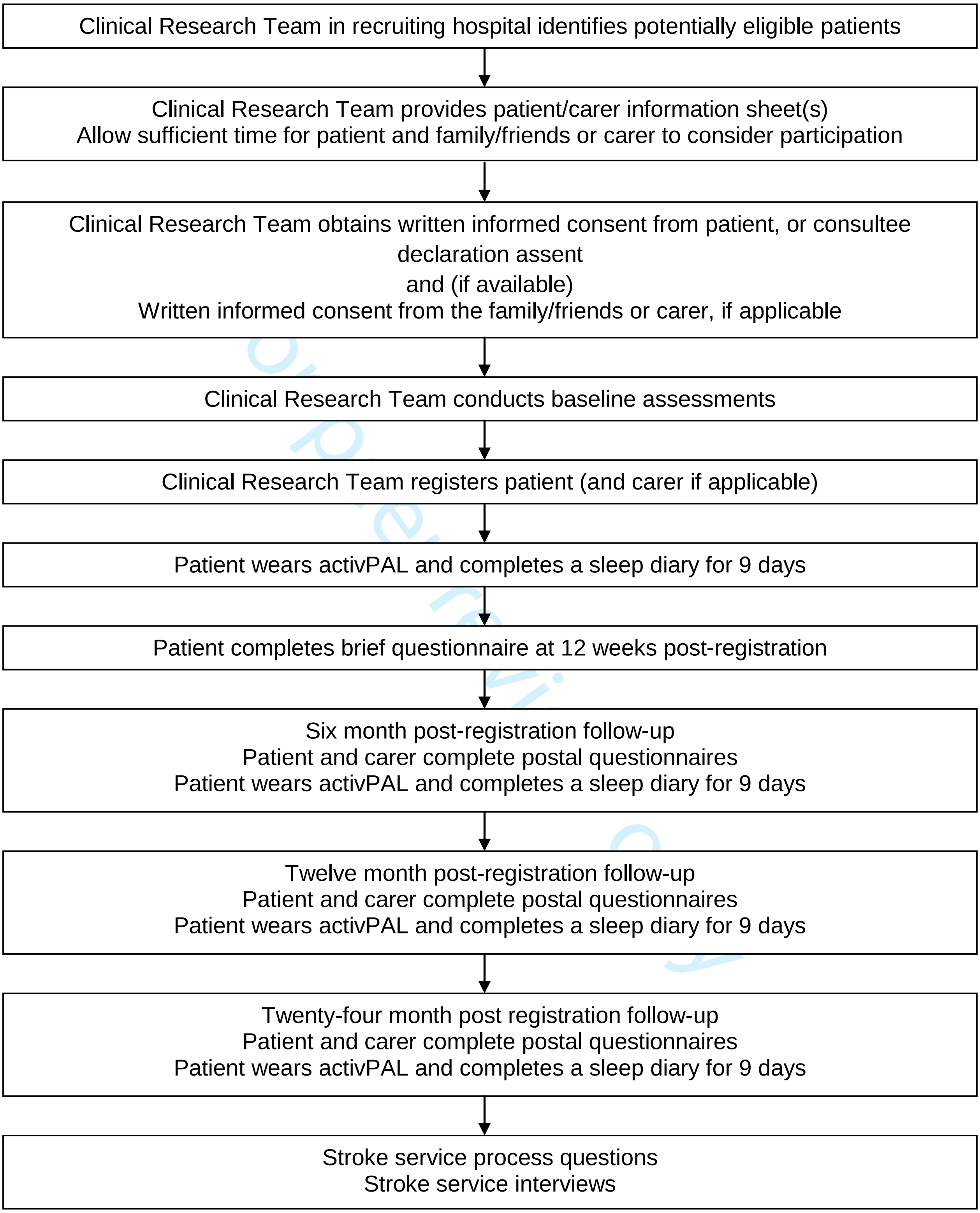

For peer review only - http://bmjopen.bmj.com/site/about/guidelines.xhtml

Appendix 1 – Participant outcome measures

Stroke survivors

- Nottingham Extended Activities of Daily Living scale (NEADL) (1): The NEADL assesses aspects of physical and social independence performance across 22 items (score range 0–66) grouped in four categories (mobility, kitchen, domestic and leisure activities), with higher scores indicating greater independence.
- World Health Organisation Disability Assessment Schedule 2.0 (12-item; WHODAS 2.0-short) (2-6). The WHODAS 2.0-short (2) was designed as a self-administered questionnaire which asks about difficulties due to health conditions. Items are scored on a 5-point Likert scale (from 0 (none) to 4 (extreme) and summed, then converted into a percentage, with higher scores indicating greater disability. A unit increment in WHODAS 2.0 z-score increased mortality hazard (6).
- Warwick-Edinburgh Mental Well-being Scale (WEMWBS) (7). The WEMWBS is designed for self-completion and comprises 14 items relating to an individuals' state of mental well-being in the previous two weeks. The scale is scored by summing responses to each item answered on a five-point Likert scale; total scores range from 14-70, with higher scores indicating higher levels of mental well-being.
- European Quality of Life 5-Dimension Health Questionnaire (5 levels; EQ-5D-5L) (8). The non-disease-specific EQ-5D-5L instrument will be used to measure health related quality of life across five dimensions: mobility, self-care, usual activities, pain/discomfort, and anxiety/depression. Each dimension is scored on a five-point scale, from 1 (no problems) to 5 (unable/ extreme problems); scores from the five dimensions are combined into a five-digit number to describe current health state. A visual analogue scale is also provided for participants to self-rate their health. The scale was developed to yield utility values which can be used to calculate QALY gains or losses, and thus will facilitate the health economic evaluation. Participants

For peer review only - http://bmjopen.bmj.com/site/about/guidelines.xhtml

For peer review only

are also asked to self-rate their health on the accompanying vertical Visual Analogue Scale, with the best and worst health they can imagine as endpoints.

- Fatigue Assessment Scale (FAS) (9, 10). The FAS is a 10-item questionnaire consisting of five questions relating to mental fatigue and five questions for physical fatigue. Participants are asked to rate how they usually feel on a five-point scale from 1 (never) to 5 (always), with total scores ranging from 10 to 50. Higher scores indicate increased fatigue.
- Measure of Older Adults' Sedentary Time (MOST) (11) and Sedentary Behaviour Visual Analogue Scale (adapted from (12)). The MOST asks participants to estimate the amount of time spent in seven sedentary activities during the previous week.
- Whether they have fallen and whether or not this led to injury (13). At baseline this will be related to in hospital falls and falls during the three months prior to admission
- Whether they use a walking aid
- Health and social care service use and informal care inputs

Carers

Modified Caregiver Strain Index (MCSI (14)). The MCSI is a 13-item measure of strain related to care provision covering five domains: financial; physical; psychological; social; and personal. Each item is scored 0 (no), 1 (sometimes) or 2 (yes), with total scores ranging from 0-26. Higher scores indicate higher levels of caregiver strain.

For peer review only - http://bmjopen.bmj.com/site/about/guidelines.xhtml

References

1. Nouri F, Lincoln N. An extended activities of daily living scale for stroke patients. Clinical rehabilitation. 1987;1(4):301-5.

2. Üstün TB, Chatterji S, Kostanjsek N, et al. Developing the World Health Organization disability assessment schedule 2.0. Bulletin of the World Health Organization. 2010;88:815-23.

3. Schlote A, Richter M, Wunderlich M, et al. WHODAS II with people after stroke and their relatives. Disability and rehabilitation. 2009;31(11):855-64.

4. Garin O, Ayuso-Mateos JL, Almansa J, et al. Validation of the" World Health Organization Disability Assessment Schedule, WHODAS-2" in patients with chronic diseases. Health and quality of life outcomes. 2010;8:1-15.

5. Küçükdeveci AA, Kutlay Ş, Yıldızlar D, et al. The reliability and validity of the World Health Organization Disability Assessment Schedule (WHODAS-II) in stroke. Disability and rehabilitation. 2013;35(3):214-20.

6. Hirve S, Juvekar S, Sambhudas S, et al. Does self-rated health predict death in adults aged 50 years and above in India? Evidence from a rural population under health and demographic surveillance. International journal of epidemiology. 2012;41(6):1719-27.

7. Tennant R, Hiller L, Fishwick R, et al. The Warwick-Edinburgh mental well-being scale (WEMWBS): development and UK validation. Health and Quality of life Outcomes. 2007;5(1):1-13.

8. Herdman M, Gudex C, Lloyd A, et al. Development and preliminary testing of the new five-level version of EQ-5D (EQ-5D-5L). Quality of life research. 2011;20:1727-36.

9. Mead G, Lynch J, Greig C, et al. Evaluation of fatigue scales in stroke patients. Stroke. 2007;38(7):2090-5.

10. Michielsen HJ, De Vries J, Van Heck GL. Psychometric qualities of a brief self-rated fatigue measure: The Fatigue Assessment Scale. Journal of psychosomatic research. 2003;54(4):345-52.

For peer review only - http://bmjopen.bmj.com/site/about/guidelines.xhtml

11. Gardiner PA, Eakin EG, Healy GN, et al. Feasibility of reducing older adults' sedentary time. American journal of preventive medicine. 2011;41(2):174-7.

12. Chastin SF, Dontje ML, Skelton DA, et al. Systematic comparative validation of self-report measures of sedentary time against an objective measure of postural sitting (activPAL). International Journal of Behavioral Nutrition and Physical Activity. 2018;15(1):1-12.

13. Perry L, Kendrick D, Morris R, et al. Completion and return of fall diaries varies with participants' level of education, first language, and baseline fall risk. Journals of Gerontology Series A: Biomedical Sciences and Medical Sciences. 2012;67(2):210-4.

14. Thornton M, Travis SS. Analysis of the reliability of the modified caregiver strain index. The Journals of Gerontology Series B: Psychological Sciences and Social Sciences. 2003;58(2):S127-S32.

For peer review only

For peer review only - http://bmjopen.bmj.com/site/about/guidelines.xhtml

STANDARD PROTOCOL ITEMS: RECOMMENDATIONS FOR INTERVENTIONAL TRIALS

SPIRIT 2013 Checklist: Recommended items to address in a clinical trial protocol and related documents*

Section/item	Item No	Description	Addressed on page number
**Administrative information**			
Title	1	Descriptive title identifying the study design, population, interventions, and, if applicable, trial acronym	Page 1
Trial registration	2a	Trial identifier and registry name. If not yet registered, name of intended registry	Page 2
	2b	All items from the World Health Organization Trial Registration Data Set	Throughout
Protocol version	3	Date and version identifier	Page 24
Funding	4	Sources and types of financial, material, and other support	Page 31
Roles and responsibilities	5a	Names, affiliations, and roles of protocol contributors	Page 1
	5b	Name and contact information for the trial sponsor	Page 31
	5c	Role of study sponsor and funders, if any, in study design; collection, management, analysis, and interpretation of data; writing of the report; and the decision to submit the report for publication, including whether they will have ultimate authority over any of these activities	Page 31
	5d	Composition, roles, and responsibilities of the coordinating centre, steering committee, endpoint adjudication committee, data management team, and other individuals or groups overseeing the trial, if applicable (see Item 21a for data monitoring committee)	Page 23
**Introduction**			
Background and rationale	6a	Description of research question and justification for undertaking the trial, including summary of relevant studies (published and unpublished) examining benefits and harms for each intervention	Page 4 - 5
	6b	Explanation for choice of comparators	Page 9
Objectives	7	Specific objectives or hypotheses	Page 5 - 6

For peer review only

Trial design	8	Description of trial design including type of trial (eg, parallel group, crossover, factorial, single group), allocation ratio, and framework (eg, superiority, equivalence, noninferiority, exploratory)	Page 6
**Methods: Participants, interventions, and outcomes**			
Study setting	9	Description of study settings (eg, community clinic, academic hospital) and list of countries where data will be collected. Reference to where list of study sites can be obtained	Page 7
Eligibility criteria	10	Inclusion and exclusion criteria for participants. If applicable, eligibility criteria for study centres and individuals who will perform the interventions (eg, surgeons, psychotherapists)	Page 7 - 9
Interventions	11a	Interventions for each group with sufficient detail to allow replication, including how and when they will be administered	Page 9 - 10
	11b	Criteria for discontinuing or modifying allocated interventions for a given trial participant (eg, drug dose change in response to harms, participant request, or improving/worsening disease)	Page 10
	11c	Strategies to improve adherence to intervention protocols, and any procedures for monitoring adherence (eg, drug tablet return, laboratory tests)	Page 10
	11d	Relevant concomitant care and interventions that are permitted or prohibited during the trial	Page 9
Outcomes	12	Primary, secondary, and other outcomes, including the specific measurement variable (eg, systolic blood pressure), analysis metric (eg, change from baseline, final value, time to event), method of aggregation (eg, median, proportion), and time point for each outcome. Explanation of the clinical relevance of chosen efficacy and harm outcomes is strongly recommended	Page 11 - 15
Participant timeline	13	Time schedule of enrolment, interventions (including any run-ins and washouts), assessments, and visits for participants. A schematic diagram is highly recommended (see Figure)	Page 16 - 19
Sample size	14	Estimated number of participants needed to achieve study objectives and how it was determined, including clinical and statistical assumptions supporting any sample size calculations	Page 7
Recruitment	15	Strategies for achieving adequate participant enrolment to reach target sample size	Page 7
**Methods: Assignment of interventions (for controlled trials)**			
Allocation:			
Sequence generation	16a	Method of generating the allocation sequence (eg, computer-generated random numbers), and list of any factors for stratification. To reduce predictability of a random sequence, details of any planned restriction (eg, blocking) should be provided in a separate document that is unavailable to those who enrol participants or assign interventions	Page 15
Allocation concealment mechanism	16b	Mechanism of implementing the allocation sequence (eg, central telephone; sequentially numbered, opaque, sealed envelopes), describing any steps to conceal the sequence until interventions are assigned	Page 15

For peer review only - http://bmjopen.bmj.com/site/about/guidelines.xhtml

For peer review only

Implementation	16c	Who will generate the allocation sequence, who will enrol participants, and who will assign participants to interventions	Page 15
Blinding (masking)	17a	Who will be blinded after assignment to interventions (eg, trial participants, care providers, outcome assessors, data analysts), and how	Page 16
	17b	If blinded, circumstances under which unblinding is permissible, and procedure for revealing a participant's allocated intervention during the trial	n/a
**Methods: Data collection, management, and analysis**			
Data collection methods	18a	Plans for assessment and collection of outcome, baseline, and other trial data, including any related processes to promote data quality (eg, duplicate measurements, training of assessors) and a description of study instruments (eg, questionnaires, laboratory tests) along with their reliability and validity, if known. Reference to where data collection forms can be found, if not in the protocol	Page 16 - 19, Appendix 1
	18b	Plans to promote participant retention and complete follow-up, including list of any outcome data to be collected for participants who discontinue or deviate from intervention protocols	Page 18 - 19
Data management	19	Plans for data entry, coding, security, and storage, including any related processes to promote data quality (eg, double data entry; range checks for data values). Reference to where details of data management procedures can be found, if not in the protocol	Page 19
Statistical methods	20a	Statistical methods for analysing primary and secondary outcomes. Reference to where other details of the statistical analysis plan can be found, if not in the protocol	Page 19 - 21
	20b	Methods for any additional analyses (eg, subgroup and adjusted analyses)	Page 21
	20c	Definition of analysis population relating to protocol non-adherence (eg, as randomised analysis), and any statistical methods to handle missing data (eg, multiple imputation)	Page 21 - 22
**Methods: Monitoring**			
Data monitoring	21a	Composition of data monitoring committee (DMC); summary of its role and reporting structure; statement of whether it is independent from the sponsor and competing interests; and reference to where further details about its charter can be found, if not in the protocol. Alternatively, an explanation of why a DMC is not needed	Page 23
	21b	Description of any interim analyses and stopping guidelines, including who will have access to these interim results and make the final decision to terminate the trial	n/a - No formal interim or sub-group analyses are planned, and final analysis will take place when

For peer review only

			all available data have been received.
Harms	22	Plans for collecting, assessing, reporting, and managing solicited and spontaneously reported adverse events and other unintended effects of trial interventions or trial conduct	Page 23 - 24
Auditing	23	Frequency and procedures for auditing trial conduct, if any, and whether the process will be independent from investigators and the sponsor	Page 19, 23, 31
**Ethics and dissemination**			
Research ethics approval	24	Plans for seeking research ethics committee/institutional review board (REC/IRB) approval	Page 24 - 25
Protocol amendments	25	Plans for communicating important protocol modifications (eg, changes to eligibility criteria, outcomes, analyses) to relevant parties (eg, investigators, REC/IRBs, trial participants, trial registries, journals, regulators)	Page 24 - 25
Consent or assent	26a	Who will obtain informed consent or assent from potential trial participants or authorised surrogates, and how (see Item 32)	Page 16 - 17
	26b	Additional consent provisions for collection and use of participant data and biological specimens in ancillary studies, if applicable	n/a
Confidentiality	27	How personal information about potential and enrolled participants will be collected, shared, and maintained in order to protect confidentiality before, during, and after the trial	Page 19
Declaration of interests	28	Financial and other competing interests for principal investigators for the overall trial and each study site	Page 31 - 32
Access to data	29	Statement of who will have access to the final trial dataset, and disclosure of contractual agreements that limit such access for investigators	Page 31
Ancillary and post-trial care	30	Provisions, if any, for ancillary and post-trial care, and for compensation to those who suffer harm from trial participation	n/a
Dissemination policy	31a	Plans for investigators and sponsor to communicate trial results to participants, healthcare professionals, the public, and other relevant groups (eg, via publication, reporting in results databases, or other data sharing arrangements), including any publication restrictions	Page 24 - 25
	31b	Authorship eligibility guidelines and any intended use of professional writers	Page 24 - 25
	31c	Plans, if any, for granting public access to the full protocol, participant-level dataset, and statistical code	Page 31
**Appendices**			
Informed consent	32	Model consent form and other related documentation given to participants and authorised surrogates	Not included -

For peer review only

materials			available on request
Biological specimens	33	Plans for collection, laboratory evaluation, and storage of biological specimens for genetic or molecular analysis in the current trial and for future use in ancillary studies, if applicable	n/a

*It is strongly recommended that this checklist be read in conjunction with the SPIRIT 2013 Explanation & Elaboration for important clarification on the items. Amendments to the protocol should be tracked and dated. The SPIRIT checklist is copyrighted by the SPIRIT Group under the Creative Commons "Attribution-NonCommercial-NoDerivs 3.0 Unported" license.

For peer review only

BMJ Open

RECREATE: A study protocol for a multi-centre pilot cluster Randomised Controlled Trial in UK stroke services evaluating an intervention to reduce sedentary behaviour in stroke survivors (Get Set Go) with embedded process and economic evaluations

Journal:	*BMJ Open*
Manuscript ID	bmjopen-2023-074607.R1
Article Type:	Protocol
Date Submitted by the Author:	14-Jun-2023
Complete List of Authors:	Airlie, Jennifer; Bradford Institute for Health Research, Academic Unit for Ageing and Stroke Research<br>Burton, Louisa-Jane; Bradford Institute for Health Research, Academic Unit for Ageing and Stroke Research<br>Copsey, Bethan; University of Leeds, Clinical Trials Research Unit (CTRU)<br>English, Coralie; The University of Newcastle, School of Health Sciences; The University of Newcastle Hunter Medical Research Institute, Heart and Stroke Research Program<br>Farrin, Amanda; University of Leeds, Clinical Trials Research Unit (CTRU)<br>Fitzsimons, Claire F.; University of Edinburgh Institute for Sport Physical Education and Health Sciences, Physical Activity for Health and Research Centre<br>Holloway, Ivana; University Medical Center Göttingen, Institute of Medical Psychology and Medical Sociology<br>Horrocks, Judith; University of Leeds, Clinical Trials Research Unit (CTRU)<br>Johansson, Jessica; University of Leeds, Academic Unit for Ageing and Stroke Research<br>Mead, Gillian; University of Edinburgh, Centre for Clinical Brain Sciences<br>Moreau, Lauren; University of Leeds, Clinical Trials Research Unit (CTRU)<br>Ozer, Seline; Bradford Institute for Health Research, Academic Unit for Ageing and Stroke Research<br>Patel, Anita; Anita Patel Health Economics Consulting Ltd<br>Yaziji, Nahel; King's College London, Institute of Psychiatry, Psychology and Neuroscience<br>Forster, Anne; Bradford Institute for Health Research, Academic Unit for Ageing and Stroke Research; University of Leeds, Academic Unit for Ageing and Stroke Research
**Primary Subject Heading**:	Rehabilitation medicine
Secondary Subject Heading:	Health services research
Keywords:	Clinical Trial, REHABILITATION MEDICINE, Stroke < NEUROLOGY

SCHOLARONE™
Manuscripts

Title: RECREATE: A study protocol for a multi-centre pilot cluster Randomised Controlled Trial in UK stroke services evaluating an intervention to reduce sedentary behaviour in stroke survivors (Get Set Go) with embedded process and economic evaluations

Jennifer Airlie[1], Louisa-Jane Burton[1], Bethan Copsey[2], Coralie English[3,4], Amanda Farrin[2], Claire Fitzsimons[5], Ivana Holloway[6], Judith Horrocks[2] , Jessica Faye Johansson[7], Gillian Mead[8], Lauren Moreau[2], Seline Ozer[1], Anita Patel[9], Nahel Yaziji[10], Anne Forster[1,7] on behalf of the RECREATE Programme Management Group

[1.] Academic Unit for Ageing and Stroke Research, Bradford Institute for Health Research, Bradford Teaching Hospitals NHS Foundation Trust, Bradford, UK

[2.] Clinical Trials Research Unit (CTRU), Leeds Institute of Clinical Trials Research, University of Leeds, Leeds, UK

[3.] School of Health Sciences, University of Newcastle, Newcastle, Australia.

[4.] Heart and Stroke Research Program, Hunter Medical Research Institute, Newcastle, Australia

[5.] Physical Activity for Health Research Centre, University of Edinburgh, Edinburgh, UK

[6.] Institute of Medical Psychology and Medical Sociology, University Medical Center Göttingen, Waldweg 37A, 37073, Göttingen, Germany

[7.] Academic Unit for Ageing and Stroke Research, Leeds Institute of Health Sciences, University of Leeds, Leeds, UK

[8.] Centre for Clinical Brain Sciences, University of Edinburgh, Edinburgh, UK

[9.] Anita Patel Health Economics Consulting Ltd, London, UK.

[10.] King's College London, Institute of Psychiatry Psychology and Neuroscience, London, UK.

Correspondence to Dr Jennifer Airlie, Academic Unit for Ageing and Stroke Research, Bradford Institute for Health Research, Bradford Teaching Hospitals NHS Foundation Trust, Bradford, BD9 6RJ, UK. Email: Jennifer.Airlie@bthft.nhs.uk

For peer review only

For peer review only - http://bmjopen.bmj.com/site/about/guidelines.xhtml

Abstract

Introduction: Sedentary behaviour (sitting or lying during waking hours without being otherwise active) is strongly associated with adverse health outcomes, including all-cause, cancer, and cardiovascular mortality in adults. Stroke survivors are consistently reported as being more sedentary than healthy age-matched controls, spending more hours sedentary daily and sustaining longer unbroken bouts of sedentary time. An evidence-based and clinically feasible intervention ('Get Set Go') was developed. A pragmatic definitive trial to evaluate Get Set Go was planned; however, due to the unprecedented effects of the COVID-19 pandemic on National Health Service (NHS) services this study was reduced in size and scope to become an external pilot trial. We report the protocol for this external pilot trial, which aims to undertake a preliminary exploration of whether Get Set Go is likely to improve ability to complete extended activities of daily living in the first year post-stroke and inform future trial designs in stroke rehabilitation.

Methods and analysis: This study is a pragmatic multi-centre, two-arm, external pilot cluster Randomised Controlled Trial (cRCT) with embedded process and economic evaluations. UK-based stroke services will be randomized 1:1 to the intervention (usual care plus Get Set Go) or control (usual care) arm. Fifteen stroke services will recruit 300-400 stroke in-patient and carer participants, with follow-up at six, 12 and 24 months. The proposed primary endpoint is stroke survivor self-reported Nottingham Extended Activities of Daily Living (NEADL) scale. Endpoint analyses will be exploratory and provide preliminary estimates of intervention effect. The process evaluation will provide valuable information on intervention fidelity, acceptability, and how it can be optimised.

Ethics and dissemination: The study has been approved by Yorkshire & The Humber-Bradford Leeds Research Ethics Committee (Ref: 19/YH/0403). Results will be disseminated through journal publications and conference presentations.

Trial registration: This trial was registered prospectively on 01/04/2020 (ISRCTN ref: 82280581).

For peer review only - http://bmjopen.bmj.com/site/about/guidelines.xhtml

Strengths and limitations of this study

- An external pilot cluster randomised control trial (cRCT) design enables the collection of feasibility and acceptability data to inform future trial designs in stroke rehabilitation.
- The comprehensive set of outcome measures collected will ensure any potential impacts of the intervention on meaningful outcomes for stroke survivors (e.g. short- and long-term changes to activities of daily living, quality of life and well-being) are captured.
- The study will collect objective measures of sedentary behaviour (using activity monitors), which to date have been infrequently assessed in post-stroke rehabilitation trials with longer term outcomes up to 24 months.
- The inclusion of an embedded process evaluation (reported separately) will provide valuable information on the intervention fidelity, acceptability, and how it can be optimised.
- The inclusion of a nested Study Within A Trial (SWAT) (reported separately) enables the systematic evaluation of whether a video animation could enhance participant understanding and subsequently uptake, engagement and compliance with the intervention and thus inform how best to implement the intervention in practice.

Introduction

Background and rationale

There are clear associations between sedentary behaviour in the general population and increased risk of adverse health outcomes, including all-cause, cancer, and cardiovascular mortality, and incidence of cardiovascular disease, cancer and Type II diabetes (1, 2). Sedentary behaviour is defined as any waking behaviour characterised by low energy expenditure ≤1.5 Metabolic Equivalent of Task (METs) while in a sitting, lying or reclining posture (3), and importantly differs from physical inactivity, which refers to achieving insufficient levels of moderate to vigorous physical activity (MVPA). An individual may therefore not reach the recommended levels of physical activity yet spend little time sitting, whereas others may be physically active for short

For peer review only - http://bmjopen.bmj.com/site/about/guidelines.xhtml

bursts (e.g., running for an hour), but spend prolonged periods sitting. Increasing MVPA can offset some of the detrimental effects of sedentary behaviour, although high levels are required (i.e., >300 minutes/ week) (1).

Stroke survivors are more sedentary than other population groups (4-6). Longitudinal studies (7); systematic reviews (8, 9) and observational studies (6) reported that stroke survivors are considerably more sedentary (in hours per day) and have longer unbroken bouts of sedentary time than healthy age-matched controls. Sedentary behaviour does not improve over the first year after stroke (7) and appears independent of the level of functional recovery (7, 8, 10), with physical ability only having a small influence on time spent sitting in people living at home six months after stroke (10). The high amount of reported sitting time by stroke survivors is likely to generate a range of negative outcomes. Epidemiological studies (11) place them in the highest quartile for cardiovascular risk. For a stroke survivor, increased sedentary behaviour may thus result in a "perfect storm" of deteriorating physical function and health-related quality of life, coincident with rising risks for cardiovascular disease.

There has been increasing focus in international public health guidance on reducing sedentary behaviour, particularly for those with disabilities, such as those resulting from stroke, as for many it may be a more achievable target than increasing PA (12). Short brief activity breaks throughout the day may be as effective as a continuous 30- or 60-minute bout of exercise (summarised in (13)). Experimental studies (primarily short-term, laboratory-based work) provide supporting evidence of the positive effect on metabolic outcomes of breaking up sitting time with short burst of activity in standing (14, 15) and reducing stroke risk (16).

Furthermore, the potential benefits of reducing sedentary behaviour reach beyond those relating to mortality and cardiovascular risk. Indeed, sedentary behaviour has been identified as a modifiable behaviour to enhance physical function (17) and maintain muscle strength, thus potentially supporting the ability to undertake activities of daily living (ADLs) (17-19). These benefits are important not just for stroke survivors themselves but may also extend to their carers; a recent

For peer review only - http://bmjopen.bmj.com/site/about/guidelines.xhtml
For peer review only

systematic review identified post-stroke disability as the biggest predictor of carer burden (20). Improved physical function in stroke survivors may thus reduce carer strain. Sedentary behaviour has also been identified as important in relation to mental health, cognitive outcomes, and health-related quality of life (21).

To address the challenge of reducing sedentary behaviour after stroke, we embarked upon a programme of work (RECREATE) to develop an evidence-based and clinically feasible intervention to reduce/ break up sedentary behaviour in stroke survivors. Intervention development was informed by systematic reviews and qualitative work, and underpinned by behaviour change theory (the Behaviour Change Wheel (22)). The final intervention ('Get Set Go') was developed using co-production methods (23). A logic model of intended intervention mechanisms and outcomes was also constructed, and implementation strategies were refined using a case study approach. The intervention is intended to begin early after stroke (in the in-patient setting) and continue into the community, and designed to fit into existing rehabilitation and recovery pathways.

This protocol represents the final stage in the RECREATE programme, which aims to evaluate the developed intervention in a sample of stroke survivors and carers. A large definitive multi-centre cluster Randomised Controlled Trial (cRCT) with 34 stroke services each recruiting 34 participants was initially planned, with the aim of evaluating the effectiveness and cost-effectiveness of the developed intervention. However, due to issues related to the worldwide COVID-19 pandemic, site recruitment was slower than expected. As a result, in Spring 2022, following meetings with the Programme Steering Committee (PSC) and a stakeholder meeting convened by the funder (National Institute for Health and care Research (NIHR)), a decision was taken with the funder to amend the protocol to reduce the trial in size and scope to become an external pilot trial.

Objectives

The primary objective is to undertake a preliminary exploration of whether the intervention (Get Set Go) is likely to improve the ability to complete Extended Activities of Daily Living (EADLs) in the

For peer review only - http://bmjopen.bmj.com/site/about/guidelines.xhtml

first year after stroke. A key secondary objective is to explore whether the intervention is likely to reduce sedentary behaviour. Other secondary objectives are to:

a) Explore core resource use and costs associated with delivering the intervention including impacts on wider care;
b) Explore whether the intervention reduces cardiovascular risk factors;
c) Explore whether the intervention reduces disability;
d) Explore whether the intervention improves health and wellbeing outcomes, such as health related quality of life and mental wellbeing;
e) Explore whether the intervention reduces carer strain;
f) Explore and understand implementation of the intervention, including intervention adherence, compliance, the process, benefits and challenges;
g) Explore and understand how the intervention is experienced and understood by recipients and providers;
h) Explore potential moderators and mediators of the intervention effect.

Objectives a-e will be addressed in the cRCT. Additional objectives addressed in the cRCT relate to gathering feasibility and acceptability data to inform future trial designs in stroke rehabilitation. Objectives f -h will be addressed in an embedded mixed-methods process evaluation (protocol reported separately), which will develop an understanding of how the intervention is implemented, understood and experienced by both providers and recipients.

Methods

Design

This trial is a pragmatic, multicentre, two-arm external pilot cRCT. The Get Set Go intervention will be delivered alongside usual care at the level of the stroke service (i.e., cluster). The cluster design will reduce between-group contamination. Eligible stroke services will be randomised on a 1:1 basis to the two arms of the trial: either intervention (usual care plus Get Set Go) or control (i.e., usual care). All stroke survivors in services allocated to the intervention arm will receive Get Set

For peer review only - http://bmjopen.bmj.com/site/about/guidelines.xhtml

Go; however outcomes will only be measured for those who consent to trial participation.

Settings

The trial will be conducted in up to 15 National Health Service (NHS) stroke services across the United Kingdom (UK). A stroke service is defined as an acute and/ or rehabilitation stroke unit with a linked community service over a defined geographical area. Service eligibility criteria are detailed in Table 1.

Table 1: Stroke service eligibility criteria

**Inclusion criteria**	**Exclusion criteria**
Both the inpatient and community service agree to participate	Previous participation in research leading to the development of the intervention
Availability of research staff to undertake participant recruitment	Currently implementing/ intending to implement similar interventions within trial duration
Agreement that recruitment targets are feasible/ acceptable (considering patient throughput)	Early Supported Discharge (ESD) (or Community if no ESD) waiting list >four weeks post-discharge
The intervention can be feasibly implemented (if so randomised)	

Recruitment

Three to four hundred stroke survivors and carers will be recruited to participate. Eligibility criteria are outlined in Table 2. Carer involvement is not a requirement for stroke survivor inclusion.

For peer review only - http://bmjopen.bmj.com/site/about/guidelines.xhtml

Table 2: Stroke survivor and carer eligibility criteria

	**Inclusion criteria**	**Exclusion criteria**
Stroke survivor	Aged ≥16 years at time of stroke	Receiving palliative care
	Clinical diagnosis of new or recurrent ischaemic or haemorrhagic (excluding subarachnoid haemorrhage) stroke	Due to be discharged outside the defined geographical area of the associated community service(s) participating in the trial.
	Requires manual contact of no more than one person to stand to prevent falling (manual contact consists of continuous or intermittent light touch to assist balance or co-ordination, i.e., not to support body weight)	
	Plan to live in the community post-discharge	
	Informed consent/ consultee declaration is provided	
Carer	Aged ≥16 years	Stroke survivor does not consent to participate
	Family member or friend regularly engaging with a stroke survivor participant (≥once per fortnight)	
	Provide informed consent	

Conversations with interested stroke services began in October 2019 in preparation for the study starting in December 2019. Recruitment of stroke survivors and carers started in January 2021. The study is projected to complete recruitment by the end of April 2023 with the follow-up due to

For peer review only

For peer review only - http://bmjopen.bmj.com/site/about/guidelines.xhtml

complete in April 2024. The end of the study is defined as the date the last participant's data item is collected.

Interventions

All stroke survivor participants (irrespective of randomisation allocation) will receive usual care within an organised stroke service. In line with the pragmatic nature of the trial, the comparator is 'usual care' as determined by local policy and practices, to draw comparison with current practice in NHS stroke services, especially given the variation in pathways. Information on the care delivered by both control and intervention sites will be captured by survey and enhanced by conversations with clinical staff (if further details are required) prior to randomisation and at regular intervals (approximately every three to six months) until the end of the trial. Observations conducted as part of the embedded process evaluation will provide additional insights into the nature of usual care provision at both control and intervention sites. This information will also provide context for trial implementation and monitor any potential contamination and/ or confounding between the two arms of the trial.

In stroke services allocated to the intervention arm, participants will also receive the Get Set Go intervention. Get Set Go is a whole-service intervention designed to be implemented and embedded within routine practice. Delivery commences in the in-patient stroke unit setting and continues into the community for at least 12 weeks post-discharge. The intervention focuses on:

a) Educating staff and stroke survivors (and their family/ friends/ carers where appropriate) about the importance of standing and moving after stroke;
b) Preparing and enabling staff to support and encourage stroke survivors to stand and move more in everyday stroke care (as part of routine practice);
c) Encouraging stroke survivors to monitor their own standing and moving, with assistance from family/ friends/ carers where appropriate.

As Get Set Go will be delivered at service level, all clinical staff members working within the stroke services randomised to deliver the intervention (i.e., inpatient and community settings) will be

For peer review only - http://bmjopen.bmj.com/site/about/guidelines.xhtml

invited to attend a training session (~one hour) to prepare for delivering Get Set Go. This will include the intervention rationale and an overview of key components. Staff will participate in practical tasks to ensure they feel confident in supporting and encouraging stroke survivors, who are capable of standing independently/ with the assistance of one person, to stand and move more in everyday stroke care (i.e., as part of routine clinical practice). A Template for Intervention Description and Replication (TIDieR) checklist (24) will be published with trial findings. There are no special criteria for discontinuing or modifying allocated interventions. Recommendations for standing and moving are made by staff as part of delivering the intervention alongside usual care in the stroke unit or community setting, based on their usual assessment techniques and clinical judgement. These recommendations will be regularly reviewed and modified in line with stroke survivors' individual capabilities and circumstances.

There are no provisions in place for post-trial care beyond the standard care provided within the participating NHS stroke services.

Study Within a Trial (SWAT)

This cRCT will also incorporate a SWAT (protocol reported separately (25), to evaluate whether a video animation could increase participant understanding of the intervention, and result in improved engagement and compliance, potentially resulting in improved outcomes. Intervention sites will be randomised 1:1 to the SWAT intervention (Get Set Go plus video animation) or control (Get Set Go with no video animation). Results will be reported separately.

Strategies to improve intervention adherence

The implementation team will maintain regular contact with clinical leads in sites allocated to the intervention arm to monitor delivery of the intervention and provide support as required. Clinical staff who are responsible for delivering the intervention will also complete monitoring records for all stroke survivors (whether or not they are trial participants) to document whether key tasks relating to the intervention have been completed (no personal data will be recorded for non-trial participants). It will be advised that a nominated member of staff checks these records have been

For peer review only

For peer review only - http://bmjopen.bmj.com/site/about/guidelines.xhtml

completed on a regular basis (e.g., towards the end of each shift). Regular review of these records will enable the study team to monitor intervention adherence and provide insight into intervention delivery. A short postal questionnaire completed by stroke survivor trial participants at 12-weeks post-hospital discharge will also assess intervention compliance, with those in the intervention arm responding to questions about the intervention; all participants will be asked about falls and use of a walking aid. Data on compliance will be analysed using descriptive summary statistics by allocation. Finally, intervention fidelity will be explored separately within the process evaluation, through site-based observations of intervention delivery, interviews with those delivering and receiving the intervention, and documentary analysis (including completed intervention materials).

Endpoints

The primary endpoint is stroke survivor self-reported Extended Activities of Daily Living (EADLs) at 12 months post-registration. The key secondary endpoint is mean daily sedentary time (minutes) at 12 months post-registration using activity monitor (ActivPAL) data, which provides a valid measure of posture and transitions in people with impaired mobility (26). Other secondary endpoints at six, 12- and 24- months post-registration (unless stated otherwise) for stroke survivor participants are listed below:

- Sedentary behaviour measured by activity monitor;
- EADLs (at six and 24 months only) measured by Nottingham Extended Activities of Daily Living scale (NEADL) (27);
- Health and disability measured by World Health Organisation Disability Assessment Schedule 2.0 (12-item; WHODAS 2.0-short) (28-32);
- Mental well-being measured by Warwick-Edinburgh Mental Well-being Scale (WEMWBS) (33);
- Health status measured by European Quality of Life 5-Dimension Health Questionnaire (5 levels; EQ-5D-5L) (34);
- Quality-Adjusted Life Year (QALY) gains (as above);
- Fatigue as measured by Fatigue Assessment Scale (FAS) (35, 36);

For peer review only - http://bmjopen.bmj.com/site/about/guidelines.xhtml
For peer review only

- Death;
- Falls and use of a walking aid (12-weeks post-discharge, and at six, 12 and 24 months);
- Self-reported sedentary behaviour measured by the Measure of Older Adults' Sedentary Time (MOST) (37) and a Sedentary Behaviour Visual Analogue Scale (adapted from (38)).
- Institutionalisation, hospital re-admission, and emergency department attendance rates;
- Cardiovascular risk markers: Body Mass Index (BMI), waist circumference, blood pressure (BP)
- Total major vascular events: Composite measure of non-fatal stroke, non-fatal myocardial infarction or death due to any vascular cause (including unexplained sudden death) (39);
- Self-reported health and social care service use and informal care inputs measured by a Client Services Receipt Inventory (CSRI) specifically adapted for this trial from versions used in previous stroke rehabilitation trials (40, 41).
- Costs from a health and social care perspective, and a societal perspective that further includes informal care.

The carer end-point is caregiver burden measured by Modified Caregiver Strain Index (MCSI (42)) at six, 12 and 24 months.

An overview of which assessments are undertaken at each time-point and the method of completion is provided in the Table 3. Information about the validity and reliability of the measures used is available in Appendix 1. Stroke survivors will also complete an additional brief postal questionnaire at 12-weeks post-discharge.

For peer review only

For peer review only - http://bmjopen.bmj.com/site/about/guidelines.xhtml

Table 3: Overview of assessments and method of completion at each study time-point

Outcome / Assessment	Method of completion	Time-point			
		Baseline	6 months post-registration	12 months post-registration	24 months post-registration
**Stroke Survivor**					
Demographic Details	Self-report	X			
Nottingham Extended Activities of Daily Living Scale (NEADL) (27)	Self-report	X	X	X	X
World Health Organisation Disability Assessment Scale (WHODAS) 2.0 12 Item (28-32)	Self-report	X	X	X	X
Warwick-Edinburgh Mental Well-being Scale (WEMWBS) (33)	Self-report	X	X	X	X
European Quality of Life 5-Dimension Health Questionnaire (EQ-5D-5L) (34)	Self-report	X	X	X	X
Fatigue Assessment Scale (FAS) (35, 36)	Self-report	X	X	X	X
Measure of Older Adults' Sedentary Time (MOST) (37)	Self-report	X	X	X	X
Sedentary Behaviour Visual Analogue Scale (adapted from	Self-report	X	X	X	X

For peer review only

For peer review only - http://bmjopen.bmj.com/site/about/guidelines.xhtml

(38))					
Use of a walking aid	Self-report	X	X	X	X
Falls question	Self-report	X	X	X	X
Hospital Admissions	Self-report / electronic health records		X	X	X
Client service receipt inventories (CSRI) to collect data on health and social care use and informal care inputs (adapted from (40, 41)	Self-report	X	X	X	X
Total major vascular events (39)	Electronic health records via routine data providers	X	X	X	X
Sedentary behaviour – activPAL measurements	Researcher administration / post	X	X	X	X
Risk markers for cardiovascular disease (Height* and weight for Body Mass Index (BMI); waist circumference; blood pressure (BP))<br>*collected at baseline only	Researcher collection from notes/ Researcher visit/ self-completion	X	X	X	X
Adverse Events (AE)	Self-report / Electronic		X	X	X

For peer review only - http://bmjopen.bmj.com/site/about/guidelines.xhtml

	health records				
Survival status			X	X	X
Admission to institution	Self-report		X	X	X
**Carer**					
Demographic Details	Self-report	X	(X)	(X)	(X)
Modified Caregiver Strain Index (MCSI) (42)	Self-report		X	X	X
Employment/ occupation status	Self-report	X	X	X	X

(X) only collected if 'new carer'

Randomisation and blinding

Site randomisation will be performed centrally by the Clinical Trials Research Unit (CTRU) and the allocation sequence will be computer-generated using a minimisation programme incorporating a random element to ensure the treatment arms are well-balanced. Eligible stroke services will be randomised on a 1:1 basis either to intervention or control, stratified by:

1. Sentinel Stroke National Audit Programme (SSNAP) grading level (43): A vs B/C/D/E (based on the latest available audit data at the time of randomisation: A=First class service, B=Good/ excellent in many aspects, C=Reasonable overall, some areas require improvement, D=Several areas require improvement, E=Substantial improvement required).

2. Single or combination of trusts: Single or combination.

3. Stroke unit size (acute): Median cut-off of ≤24 and >24 beds based on SSNAP acute organisational audit data (type 2 (solely for patients >72 hours post-stroke) and type 3 beds (used for both pre- and post-72-hour care) combined – based on the SSNAP audit data and confirmation by site).

For peer review only - http://bmjopen.bmj.com/site/about/guidelines.xhtml

For peer review only

Concealment of sequencing is ensured by the physically separate locations of the CTRU (where the randomisation outcome is stored) and the blinded study researchers. Following the randomisation, unblinded members of the study team, the Principal Investigator at the site, and clinical staff who will be involved in intervention delivery, will be informed of the randomisation outcome via secure email to facilitate organisation of intervention training in services allocated to this arm. Local researchers responsible for participant recruitment will be notified that randomisation has taken place but will remain blinded to randomisation outcome and will operate independently of the clinical staff involved in intervention delivery.

Local researchers involved in participant recruitment and in-person data collection (including at baseline, administering activity monitors and assessment of cardiovascular risk markers) will be blinded to the treatment allocation for their site. At the follow-up time-points, participant-completed outcome measures will be administered by CTRU staff via postal questionnaire, eliminating the potential for interviewer bias. The activity monitor (upon which data is recorded and stored until download) is sealed with no external indication of recording therefore it is not open to bias. Finally, data on mortality, re-admissions, and use of institutional care will be collected from independent sources and are unlikely to be subject to bias. Instances of unblinding will be recorded via staff self-report.

Participants, clinical staff involved in intervention delivery, and the statistical team analysing the outcome data, will not be blinded to treatment allocation. However, to minimise treatment bias, clinical staff involved in intervention delivery will not be overtly informed of which patients are participating in the trial. Additionally, to monitor for potential selection bias, key characteristics of included trial participants will be compared with publicly available datasets (e.g., SSNAP (43)) to monitor for evidence of selection bias.

For peer review only - http://bmjopen.bmj.com/site/about/guidelines.xhtml
For peer review only

Procedure

Eligible stroke survivors will be initially approached whilst they are an in-patient by a local researcher, who will provide verbal and written information about the trial before seeking informed consent.

To ensure that the trial population is representative of the clinical stroke population and inclusive of stroke survivors with cognitive impairment (including receptive, comprehension or language difficulties), recruitment procedures will include the use of accessible materials and procedures for consultee declaration (where appropriate), in compliance with the Mental Capacity Act 2005 (MCA) (44). As per the MCA, each stroke survivor will be assumed to have capacity unless it is established that they lack capacity. If there is any concern about capacity the local researcher will consult ward staff and/ or a family member/ carer (as appropriate) and a collective decision will be made as to whether the stroke survivor is deemed to have capacity to consent to participation in the trial. Changes in capacity will be monitored throughout the trial.

Potential stroke survivor participants who are deemed to have capacity to consent will be provided with written and verbal information about what their participation in the study would involve. Following time to consider participation (including discussion with family and/ or health professionals), those wishing to take part will provide informed consent through signing (or making a mark), or if they are unable, through witnessed verbal consent (by an independent observer). Should a potential participant be discharged prior to consent being gained and they consent to being contacted by the researcher, consent procedures will take place in their own home.

Should stroke survivors be deemed to lack capacity to provide informed consent, the researcher will seek to identify ‘a supporting family member, friend or carer’ (defined as someone who is in regular (at least weekly) contact with the stroke survivor), to support intervention delivery and outcome assessment. This individual will not necessarily be enrolled in the study themselves, unless they choose to enrol as a carer participant. If such a supporter is identified, the local researcher will either: act in accordance with an advance directive relative to research participation

For peer review only - http://bmjopen.bmj.com/site/about/guidelines.xhtml

(if available) or seek advice from a personal consultee (through Consultee declaration). If a personal consultee is approached, they will be provided with an additional information sheet outlining their role. Consultees will provide assent in person or via telephone (and signed by the local researcher, e.g., if the consultee is unable to visit the hospital prior to the stroke survivor's discharge). Carer participants will be approached by the local researcher once stroke survivors with capacity have given their verbal agreement or following provision of a consultee declaration for a stroke survivor participant who lacks capacity. Those wishing to participate will be asked to provide informed consent using the same procedure described above. Carers lacking the capacity to provide written informed consent will be excluded.

The local researcher will administer baseline questionnaires to stroke survivor and carer participants face-to-face and offer support where required. Proxy completion of questionnaires by a carer, family member or friend will be permitted (for those with and without capacity), with separate proxy questionnaires provided if required. Stroke survivors will also be asked to continuously wear an activPAL activity monitor on the thigh of their strongest/ unaffected leg for nine consecutive days, The monitor will be attached by the local researcher. They will also be asked to complete a purposely developed daily diary. This will capture if/ when the monitor has been removed; the time they went to bed and got up each day and whether they had episodes of sleep (i.e., naps) during the day. This information will assist with analysis and facilitate monitoring of adverse reactions to the materials used to attach the monitor. Local researchers will also collect data on risk markers for cardiovascular disease through face-to-face assessment or retrieval from medical records. These include height and weight (for calculation of BMI), waist circumference and blood pressure.

At the follow-up time points (six, 12- and 24-months post-registration), participant-completed outcome assessments will be administered by post in an assessment pack, including a covering letter and a pre-paid envelope for postal return. Activity monitors may be administered face-to-face by a local researcher or alternatively, be sent by post to the participant and returned from stroke survivor participants via pre-paid envelope. Cardiovascular risk markers (with the exception of height) will be collected alongside the activity monitor assessment. If it is not possible to organise a

For peer review only - http://bmjopen.bmj.com/site/about/guidelines.xhtml

face-to-face visit, with the participants' agreement, options for self-assessment will be explored. Guidance will be provided to local researchers as to the action required if a blood pressure reading is abnormal, including reporting this to a named clinician or notifying the participant's General Practitioner (GP). The participant will also be encouraged to discuss this with their GP.

At each participant follow-up, changes in circumstances, including change of address, institutionalisation, and death will be obtained from either recruiting teams, healthcare providers, participants, or their relatives. Additionally, for stroke survivors, the following data will be collected from electronic health records via routine data providers or directly from the recruiting hospital at each time-point: total major vascular events (composite of non-fatal stroke; non-fatal myocardial infarction; or death due to any vascular cause (including unexplained sudden death) (39); death; hospital admissions; emergency department attendance and institutionalisation. Stroke survivors will be made aware of the use of routine data providers (including NHS England, SystmOne, EMISWeb, and other relevant providers) at the time of study entry.

The study timeline for participants is presented in Figure 1.

[Figure 1 about here]

Initial letters, reminder letters and a participant pathway diagram will be distributed to participants to maximise data return at all time-points. The questionnaire sent to assess intervention compliance at 12-weeks post-hospital discharge will also serve as a reminder of study participation and act as a retention tool. At the follow-up time-points, reminders will be administered via telephone, text message, email, or post (according to participant preference) should the questionnaire not be returned within two weeks. If outcome assessments (with particular emphasis on the NEADL as the primary outcome), cannot be obtained by post, then (where appropriate) the assessments may be administered over the telephone or face-to-face to maximise return of data. Missing data, except individual data items collected via the postal assessment packs, will be sought until: it is received; confirmed as not available; or the trial is at analysis.

For peer review only - http://bmjopen.bmj.com/site/about/guidelines.xhtml

In instances where a participant withdraws from the trial clarification will be sought as to whether this is from all trial activities or individual components, e. g., wearing the activity monitor or consenting to be contacted about participation in process evaluation activities. Unless participants explicitly instruct otherwise, electronic health record data will continue to be collected.

Data management

Data will be monitored for quality and completeness using established verification, validation and checking processes, e.g., double data entry, and securely stored. Received activPAL data will be subject to quality checks by researchers and where needed, additional details to aid interpretation will be sought from the recruiting site and/ or participant diaries. All data collected during the course of the trial will be kept strictly confidential using established processes. Data will be securely archived for ten years after the completion of the study and then destroyed.

Statistical analysis

All analyses and data summaries will be conducted on the intention-to-treat population (ITT) (45), defined as all participants registered to the trial, regardless of non-compliance with the protocol or withdrawal from the study or losses to follow-up. All participants recruited within a stroke service will be analysed according to the randomised allocation for that stroke service. No formal interim or sub-group analyses are planned, and final analysis will take place when all available data have been received.

Sample size

As previously described, our original plan for a large definitive multi-centre RCT was reduced in size and scope, due to issues related to the COVID-19 pandemic. We aim to recruit 300-400 participants across 15 stroke services. This revised sample size is based on the anticipated recruitment rate within the remaining time available in the funded programme, estimated at two participants per month per site. As intervention effectiveness will not be evaluated and all analyses will be of an exploratory nature, the study does not have a formal power calculation.

For peer review only - http://bmjopen.bmj.com/site/about/guidelines.xhtml
For peer review only

Primary endpoint analysis

In line with study objectives, all endpoint analyses will be exploratory and provide preliminary estimates of intervention effect. The primary outcome will be compared between randomised groups using multi-level mixed effects model (46) adjusting for design effects and other relevant known predictors of outcome, with participants nested within services, with services treated as random effects. If appropriate, missing data will be multiply imputed at the individual participant level. Parameter estimates will be reported with 95% confidence intervals. Sensitivity analyses of the primary endpoint will be conducted to assess the impact of missing data, the choice of imputation model and of assuming data are missing not at random, as appropriate. For the primary analysis, missing data will be assumed missing at random.

Incorporating the SWAT into the statistical analysis

For the primary analysis, the SWAT intervention will be accounted for in the regression model using an interaction term. Sensitivity analyses will explore the impact of the SWAT on the Get Set Go intervention effect, for example, analysing the outcomes as a 3-arm study separating the two SWAT arms. This will be described in further detail in the statistical analysis plan.

Secondary endpoints analyses

Secondary outcomes will be analysed in a similar manner to the primary outcome. For secondary outcomes, summary statistics will be presented for each time point by treatment group (means, standard deviations, medians, minimum, maximum, and quartiles for continuous variables, and counts and percentages for categorical variables). Secondary outcomes will be analysed using the same approach as the primary outcome with the relevant model for the type of outcome variable using multi-level linear or logistic regression, with multiple imputation for missing data where appropriate. Continuous distributions will be transformed where residuals are non-normal.

Self-reported falls, institutionalisation, re-admission, cardiovascular risk markers, total major vascular events and death rates between treatment arms will be summarised descriptively.

For peer review only - http://bmjopen.bmj.com/site/about/guidelines.xhtml

For peer review only

Quantitative summaries of intervention delivery will evaluate uptake of the intervention, adherence to the processes, staff and participant engagement and quality of intervention delivery, overall and by each site. Data on usual care will be summarised descriptively.

Activity monitor (activPAL) data

Sedentary time, as measured by activPAL activity monitors, will be analysed as total sedentary minutes on an average day as well as the percentage of sedentary time during wake time. Data from the monitors will be automatically recorded and processed using the activPAL software (PAL Technologies Ltd.). We will process data, remove sleep time and calculate, for each day, the total sedentary time; total sitting time accumulated in bouts ≥30 minutes, and bouts ≥ 60 minutes, number of sedentary bouts ≥30 minutes and bouts ≥ 60 minutes, total standing time, total stepping time and number of sit-to-stand transitions.

We will then calculate the key secondary outcome of mean daily sedentary time for the days when the monitor was worn. All other sedentary behaviour secondary outcomes will be calculated in the same way. The sedentary behaviour outcomes would ideally be based on seven days of continuous wear of the monitor. The calculation of sleep time, definition of a valid wear day and number of valid wear days required for inclusion in the analysis will be informed by the results of earlier feasibility work.

Further secondary analyses

Potential moderator variables: Potential predictors of response to the intervention will be explored via inclusion of key baseline stroke survivor characteristics (identified in our earlier work) as interaction effects in the primary analysis model, to understand who might benefit from the intervention.

Potential mediator variables will be explored by modelling the relationship between process variables (e.g., intervention adherence, number/ frequency of prompts and visits from staff, participant self-reported use of the guide, mediators such as fatigue or mood, etc.) and the primary

For peer review only - http://bmjopen.bmj.com/site/about/guidelines.xhtml

outcome.

Methods in analysis to handle protocol non-adherence and any statistical methods to handle missing data

All analyses and data summaries will be conducted on the ITT population. Missing data is anticipated at the item, scale and assessment levels. Missing data on key variables/ questionnaires will be explored. Missing data will be multiply imputed, where appropriate. Reasons for missing participant assessments will be explored and considered in the imputation process (e.g., died, moved away, etc.). If someone dies, they will be classed as lost to follow-up. When individual scales within the assessment questionnaire have missing items, scoring instructions specific to the scale will be followed, or if no such instructions exist, the summary scores will be calculated from the non-missing items using multiple imputation under the missing at random (MAR) mechanism, as long as at least 75% items are non-missing. We will test missing data assumptions in a sensitivity analysis.

Health economic analysis

The embedded health economic evaluation will assess resource use, costs and outcomes to inform data collection approaches for a future definitive trial, specifically:

- Alternative approaches to measuring hospital resource use (routine records versus self-report);
- A short (6-month) versus medium (12-month) versus longer (24-month) analytical time horizon; and
- A reduced list (narrow perspective) evaluation based on routine data versus long list (comprehensive perspective) evaluation incorporating self-reported data.

For peer review only

For peer review only - http://bmjopen.bmj.com/site/about/guidelines.xhtml

Two perspectives will be considered, as relevant to the analyses: health/social care (including institutionalisation), and a wider perspective covering health/social care (including institutionalisation) plus informal care given the significance of such inputs for this patient group.

Resource use data will be collected using a combination of NHS England secondary care data and self-report approaches via the CSRI. Resource use will be combined with relevant unit costs, using within-programme estimates related to the intervention, national estimates for other health and social care resources, and both an opportunity cost and replacement cost approach in turn for informal care. Total costs will be computed for each individual according to each of the estimation alternatives set out above. Differences between the two trial arms will be compared using bootstrap regression methods due to the expected skewness in data distributions.

Exploratory cost-effectiveness analyses will combine total costs with the primary outcome and with QALY gains estimated by attaching relevant general population utility weights to EQ-5D-5L health states at each time point. Uncertainty will be analysed using cost-effectiveness planes and cost-effectiveness acceptability curves (CEACs). Sensitivity analyses will explore the potential impact on cost-effectiveness from different implementation/costing scenarios. This would better inform future trial design and implementation discussions. Other relevant sensitivity analyses will be determined during the study and will be specified in the analysis plan prior to analyses.

The economic analyses will follow the same broad principles as the outcomes analyses, e.g. use of a signed off detailed analysis plan prior to analysis, adherence to ITT principles, imputation to account for missing values as appropriate, inclusion of baseline and other covariates for the comparisons, accounting for the clustering of patients within stroke services.

Oversight and monitoring

This trial is the final study in a seven-year Programme Grant for Applied Research funded by the NIHR. As such, a Programme Management Group (PMG), chaired by the Chief Investigator (CI) and attended by the programme co-applicants meets at least every four months, for strategic

For peer review only

For peer review only - http://bmjopen.bmj.com/site/about/guidelines.xhtml

oversight and management of the programme, and to monitor progress against key objectives. An independent Programme Steering Committee (PSC) chaired and attended by independent experts meets as a minimum every 12 months, and will provide external guidance and monitor progress. Finally, a Trial Management Group (TMG) comprised of the CI, key co-applicants, study researchers and key members of the project delivery team at the CTRU will be convened to meet every month to oversee day-to-day management of the trial. A Data Monitoring Committee is not required due to the lack of serious adverse events anticipated to be due to the intervention. The PSC will take on the role of monitoring safety concerns.

Adverse event reporting and harms

Adverse Events (AEs) such as falls and musculoskeletal injuries represent an inherent consequence of an active rehabilitation process and therefore cannot be entirely avoided. Similarly, in this patient population, acute illness resulting in hospitalisation, new medical problems and deterioration of existing medical problems are expected. In recognition of this, events fulfilling the definition of an AE or serious adverse events (SAE) will not be reportable in this study unless they result from administration of any research procedures and therefore fulfil the definition of an Unexpected and Related Serious AE, or if they constitute one of the following: death, hospital readmission, institutionalisation, treatment on an emergency out-patient basis or emergency department attendance. The latter will be reported at six-, 12- and 24- months post-registration follow-up by the clinical research team using health and social care records. All Related/ Unexpected Serious AEs occurring from the date of consent up to twenty-four months post registration must be reported within 24 hours of the clinical research staff becoming aware of the event. All Related/ Unexpected Serious AEs will be reviewed by the CI and subject to expedited reporting to the Sponsor and the main REC by the CTRU on behalf of the CI within 15 days.

Patient and Public Involvement (PPI)

PPI is central to this research. The research question was informed by initial discussion with our stroke survivor and carer group, who highlighted the need for interventions to encourage active

For peer review only - http://bmjopen.bmj.com/site/about/guidelines.xhtml

movement after stroke and contributed to grant development. PPI is subsequently being sought at every stage of the programme of research. Specific input from our PPI representatives includes: being actively involved in the design and management (e.g. steering/advisory group) of this research; assisting with developing participant information resources (including trial documents and intervention materials); contributing to ongoing reporting of the study and dissemination of emerging research findings.

Ethics and dissemination

The current protocol (v9.0 10/08/22) received ethical approval from the Yorkshire & The Humber – Bradford-Leeds REC (Ref: 19/YH/0403). Protocol amendments are processed in line with Health Research Authority and REC guidelines and processes. The results of the study will be published in peer-reviewed publications and will be presented at relevant national and international conferences. Authorship will be agreed in accordance with International Committee of Medical Journal Editors recommendations and the publication policy that has been agreed by the PMG for this programme of research. In collaboration with patient and public involvement representatives, lay reports to disseminate research findings to patient groups and clinical staff at participating sites will also be compiled.

For peer review only

For peer review only - http://bmjopen.bmj.com/site/about/guidelines.xhtml

References

1. Dempsey PC, Biddle SJ, Buman MP, et al. New global guidelines on sedentary behaviour and health for adults: broadening the behavioural targets. International Journal of Behavioral Nutrition and Physical Activity. 2020;17(1):1-12.

2. U.S. Department of Health and Human Services. Physical Activity Guidelines for Americans, 2nd edition. Washington, DC: U.S. Department of Health and Human Services; 2018.

3. Tremblay MS, Aubert S, Barnes JD, et al. Sedentary behavior research network (SBRN)–terminology consensus project process and outcome. International journal of behavioral nutrition and physical activity. 2017;14:1-17.

4. Butler EN, Evenson KR. Prevalence of physical activity and sedentary behavior among stroke survivors in the United States. Topics in stroke rehabilitation. 2014;21(3):246-55.

5. Paul L, Brewster S, Wyke S, et al. Physical activity profiles and sedentary behaviour in people following stroke: a cross-sectional study. Disability and rehabilitation. 2016;38(4):362-7.

6. English C, Healy GN, Coates A, et al. Sitting and activity time in people with stroke. Physical therapy. 2016;96(2):193-201.

7. Tieges Z, Mead G, Allerhand M, et al. Sedentary behavior in the first year after stroke: a longitudinal cohort study with objective measures. Archives of physical medicine and rehabilitation. 2015;96(1):15-23.

8. English C, Manns PJ, Tucak C, et al. Physical activity and sedentary behaviors in people with stroke living in the community: a systematic review. Physical therapy. 2014;94(2):185-96.

9. Galea SL, Lee M-J, English C, et al. Sedentary versus active behavior in people after stroke. Physical Therapy Reviews. 2015;20(1):1-7.

10. English C, Healy GN, Coates A, et al. Sitting time and physical activity after stroke: physical ability is only part of the story. Topics in stroke rehabilitation. 2016;23(1):36-42.

11. Wullems JA, Verschueren SM, Degens H, et al. A review of the assessment and prevalence of sedentarism in older adults, its physiology/health impact and non-exercise mobility counter-measures. Biogerontology. 2016;17:547-65.

For peer review only

For peer review only - http://bmjopen.bmj.com/site/about/guidelines.xhtml

12. World Health Organization. WHO guidelines on physical activity and sedentary behaviour. 2020.

13. Dempsey PC, Owen N, Biddle SJ, et al. Managing sedentary behavior to reduce the risk of diabetes and cardiovascular disease. Current diabetes reports. 2014;14:1-11.

14. Benatti FB, Ried-Larsen M. The effects of breaking up prolonged sitting time: a review of experimental studies. Medicine & Science in Sports & Exercise. 2015;47(10):2053-61.

15. English C, Janssen H, Crowfoot G, et al. Frequent, short bouts of light-intensity exercises while standing decreases systolic blood pressure: Breaking Up Sitting Time after Stroke (BUST-Stroke) trial. International journal of stroke. 2018;13(9):932-40.

16. Mackie P, Weerasekara I, Crowfoot G, et al. What is the effect of interrupting prolonged sitting with frequent bouts of physical activity or standing on first or recurrent stroke risk factors? A scoping review. PloS one. 2019;14(6):e0217981.

17. Rosenberg DE, Bellettiere J, Gardiner PA, et al. Independent associations between sedentary behaviors and mental, cognitive, physical, and functional health among older adults in retirement communities. Journals of gerontology series a: Biomedical sciences and medical sciences. 2016;71(1):78-83.

18. Ezeugwu VE, Manns PJ. The feasibility and longitudinal effects of a home-based sedentary behavior change intervention after stroke. Archives of Physical Medicine and Rehabilitation. 2018;99(12):2540-7.

19. Amaral Gomes ES, Ramsey KA, Rojer AG, et al. The association of objectively measured physical activity and sedentary behavior with (instrumental) activities of daily living in community-dwelling older adults: a systematic review. Clinical interventions in aging. 2021:1877-915.

20. Shamoun C, Harris MA. Impact of stroke survivors' physical disability on the presence of caregiver burden: a systematic review. Journal of Neuroscience Nursing. 2022;54(4):146-50.

21. Saunders DH, Mead GE, Fitzsimons C, et al. Interventions for reducing sedentary behaviour in people with stroke. Cochrane Database of Systematic Reviews. 2021(6).

For peer review only

For peer review only - http://bmjopen.bmj.com/site/about/guidelines.xhtml

22. Michie S, Van Stralen MM, West R. The behaviour change wheel: a new method for characterising and designing behaviour change interventions. Implementation science. 2011;6(1):1-12.

23. Hall J, Morton S, Hall J, et al. A co-production approach guided by the behaviour change wheel to develop an intervention for reducing sedentary behaviour after stroke. Pilot and Feasibility Studies. 2020;6(1):1-13.

24. Hoffmann TC, Glasziou PP, Boutron I, et al. Better reporting of interventions: template for intervention description and replication (TIDieR) checklist and guide. BMJ. 2014;348.

25. Ahmed S, Airlie J, Clegg A, et al. A new opportunity for enhancing trial efficiency: Can we investigate intervention implementation processes within trials using SWAT (study within a trial) methodology? Research Methods in Medicine & Health Sciences. 2022;3(3):66-73.

26. Taraldsen K, Askim T, Sletvold O, et al. Evaluation of a body-worn sensor system to measure physical activity in older people with impaired function. Physical therapy. 2011;91(2):277-85.

27. Nouri F, Lincoln N. An extended activities of daily living scale for stroke patients. Clinical rehabilitation. 1987;1(4):301-5.

28. Üstün TB, Chatterji S, Kostanjsek N, et al. Developing the World Health Organization disability assessment schedule 2.0. Bulletin of the World Health Organization. 2010;88:815-23.

29. Schlote A, Richter M, Wunderlich M, et al. WHODAS II with people after stroke and their relatives. Disability and rehabilitation. 2009;31(11):855-64.

30. Garin O, Ayuso-Mateos JL, Almansa J, et al. Validation of the "World Health Organization Disability Assessment Schedule, WHODAS-2" in patients with chronic diseases. Health and quality of life outcomes. 2010;8:1-15.

31. Küçükdeveci AA, Kutlay Ş, Yıldızlar D, et al. The reliability and validity of the World Health Organization Disability Assessment Schedule (WHODAS-II) in stroke. Disability and rehabilitation. 2013;35(3):214-20.

For peer review only

For peer review only - http://bmjopen.bmj.com/site/about/guidelines.xhtml

32. Hirve S, Juvekar S, Sambhudas S, et al. Does self-rated health predict death in adults aged 50 years and above in India? Evidence from a rural population under health and demographic surveillance. International journal of epidemiology. 2012;41(6):1719-27.

33. Tennant R, Hiller L, Fishwick R, et al. The Warwick-Edinburgh mental well-being scale (WEMWBS): development and UK validation. Health and Quality of life Outcomes. 2007;5(1):1-13.

34. Herdman M, Gudex C, Lloyd A, et al. Development and preliminary testing of the new five-level version of EQ-5D (EQ-5D-5L). Quality of life research. 2011;20:1727-36.

35. Mead G, Lynch J, Greig C, et al. Evaluation of fatigue scales in stroke patients. Stroke. 2007;38(7):2090-5.

36. Michielsen HJ, De Vries J, Van Heck GL. Psychometric qualities of a brief self-rated fatigue measure: The Fatigue Assessment Scale. Journal of psychosomatic research. 2003;54(4):345-52.

37. Gardiner PA, Eakin EG, Healy GN, et al. Feasibility of reducing older adults' sedentary time. American journal of preventive medicine. 2011;41(2):174-7.

38. Chastin SF, Dontje ML, Skelton DA, et al. Systematic comparative validation of self-report measures of sedentary time against an objective measure of postural sitting (activPAL). International Journal of Behavioral Nutrition and Physical Activity. 2018;15(1):1-12.

39. PROGRESS Collaborative Group. Randomised trial of a perindopril-based blood-pressure-lowering regimen among 6105 individuals with previous stroke or transient ischaemic attack. The Lancet. 2001;358(9287):1033-41.

40. Forster A, Dickerson J, Young J, et al. A structured training programme for caregivers of inpatients after stroke (TRACS): a cluster randomised controlled trial and cost-effectiveness analysis. The Lancet. 2013;382(9910):2069-76.

41. Forster A, Ozer S, Crocker TF, et al. Longer-term health and social care strategies for stroke survivors and their carers: the LoTS2Care research programme including cluster feasibility RCT. Programme Grants Appl Res 2021;9(3).

42. Thornton M, Travis SS. Analysis of the reliability of the modified caregiver strain index. The Journals of Gerontology Series B: Psychological Sciences and Social Sciences. 2003;58(2):S127-S32.

For peer review only

For peer review only - http://bmjopen.bmj.com/site/about/guidelines.xhtml

43. Sentinel Stroke National Audit Programme (SSNAP). Annual Public Report National Results2019. Available from: https://www.strokeaudit.org/Documents/National/Clinical/Apr2017Mar2018/Apr2017Mar2018-AnnualReport.aspx (Accessed 13 March 2023).

44. Mental Capacity Act [Internet]. The Stationary Office. 2005. Available from: https://www.legislation.gov.uk/ukpga/2005/9/contents (accessed March 2023).

45. Altman DG. Practical statistics for medical research. Boca Raton, Florida: Chapman & Hall/CRC; 1999.

46. Hayes RJ, Moulton LH. Cluster randomised trials, 2nd Ed. Boca Raton: CRC Press; 2017.

For peer review only

For peer review only - http://bmjopen.bmj.com/site/about/guidelines.xhtml

Authors' contributions: AF is lead grant holder and Chief Investigator, and will oversee the design and implementation of the trial. CE, CF and GM, are coinvestigators who have contributed to development of the protocol, and attend regular programme meetings, providing advice as needed. AFa, assisted by IH and BC, led the statistical and methodological design of the trial and is the statistical guarantor responsible for the main statistical analysis and reporting. AP leads the design of the health economic components of the trial and is responsible for the health economic analyses, assisted by NY. LM, assisted by JH, leads on data management and supports the day-to-day operation of the trial. SO, assisted by JA, is responsible for managing implementation of the intervention and delivery of the trial. LJB also assisted with managing delivery of the trial. JFJ leads the embedded process evaluation. JA leads on activPAL data implementation and data issues. JA and LJB drafted the manuscript. All authors read and approved the final manuscript. This paper is written on behalf of the RECREATE Programme Management Group who meet regularly to oversee the programme of research. All authors reviewed and approved the final manuscript.

Acknowledgements: The authors would like to acknowledge the support of the Clinical Research Network (CRN) and also express our appreciation for all of the hard work and support provided by the clinical teams who are helping to deliver this trial. The authors would also like to thank members of the Programme Steering Committee for their helpful contributions through the RECREATE programme of research.

Finally the authors would like to acknowledge the expert support of the RECREATE Programme Group who meet regularly to oversee the programme of research. The group is comprised of: Jennifer Airlie, Karen Birch, Gillian Carter, Bethan Copsey, Florence Day, Coralie English, Amanda Farrin, Alison Fergusson, Claire Fitzsimons, Anne Forster, Jessica Faye Johansson, Rebecca Lawton, Laura Marsden, Gillian Mead, Lauren Moreau, Seline Ozer, Anita Patel, Rosemary Shannon, Nahel Yaziji.

For peer review only

For peer review only - http://bmjopen.bmj.com/site/about/guidelines.xhtml

Funding statement: This work was supported by the National Institute for Health and Care Research (NIHR) under its Programme Grants for Applied Research Programme, grant number RP-PG-0615-20019). The views expressed are those of the author(s) and not necessarily those of the NIHR or the Department of Health and Social Care. The Funder has had no role in trial design, beyond setting the research question the trial addresses. Data collection, management, analysis and interpretation will remain independent of the Funder.

Sponsor: The trial sponsor is Bradford Teaching Hospitals NHS Foundation Trust, Research Management & Support Office, Bradford Institute for Health Research, Bradford, BD9 6RJ, United Kingdom. Tel: +44 (0)1274 38 2575; e-mail: jane.dennison@bthft.nhs.uk

The Sponsor will maintain oversight of trial processes, but is not involved in trial design or delivery processes. The Sponsor will not participate in data analysis or trial reporting processes.

Data sharing statement: De-identified individual participant data datasets generated and/or analysed during the current study will be available upon request from the Clinical Trials Research Unit, University of Leeds (contact CTRU-DataAccess@leeds.ac.uk in the first instance). Data will be made available at the end of the trial, i.e. usually when all primary and secondary endpoints have been met and all key analyses are complete. Data will remain available from then on for as long as CTRU retains the data.

CTRU makes data available by a 'controlled access' approach. Data will only be released for legitimate secondary research purposes, where the Chief Investigator, Sponsor and CTRU agree that the proposed use has scientific value and will be carried out to a high standard (in terms of scientific rigour and information governance and security) and that there are resources available to satisfy the request. Data will only be released in line with participants' consent, all applicable laws relating to data protection and confidentiality, and any contractual obligations to which the CTRU is subject. No individual participant data will be released before an appropriate agreement is in place

For peer review only - http://bmjopen.bmj.com/site/about/guidelines.xhtml

setting out the conditions of release. The agreement will govern data retention, usually stipulating that data recipients must delete their copy of the released data at the end of the planned project.

Competing interests statement:

AF, AFa, CE, CF, IH, GM and AP are coinvestigators on the grant funding this work therefore are partially supported by the National Institute for Health and Social Care Research (NIHR) (grant number RP-PG-0615-20019).

AF has received additional research grant support from NIHR through the following funding streams: Senior Investigator award, Health Technology Assessment (HTA) and Health and Social Care Delivery Research (HS&DR). AF has previously received support from the Stroke Association to attend the UK stroke forum and received payment from the National Institute for Health (USA) for panel membership (2021, 2022). AF is currently the chair / a member of programme steering committees for NIHR research programmes (Grant reference numbers: NIHR 202339, NIHR 202020) and has served on the following panels: NIHR Doctoral Fellowships, NIHR senior investigators committee (2019/20), NIHR HS&DR committee (2016-2018) and Stroke Association Funding.

CE has received grant funding from the Netherlands Organisation for Scientific Research (NOW) Taskforce for Applied Research (SIA RAAK) for work in a similar area (i.e. sitting less and moving more after stroke) and is a Non-executive Director representing interests of Research and Chair of Research Advisory Committee for the Stroke Foundation of Australia (unpaid).

CF is a coinvestigator / collaborator on other grants on the topic of sedentary behaviour / physical activity and is therefore partially supported by grant funding received from the University of Edinburgh and the Irish Health Board. CE has previously been supported to conduct work in a similar area by grant funding received from the Chief Scientist Office of the Scottish Government, Medical Research Council (MRC) Public Health Intervention Development (PHIND) award and the University of Edinburgh.

JA, LJB, BC, JH, JFJ, LM, SO and NY report no competing interests related to the manuscript.

For peer review only - http://bmjopen.bmj.com/site/about/guidelines.xhtml
For peer review only

Patient and public involvement: Patients and/or the public were involved in the design, conduct, reporting and dissemination plans of this research. Please refer to the methods section for further details.

Twitter: @RECREATE_stroke

Figure 1 legend: Study timeline for participants

For peer review only

For peer review only - http://bmjopen.bmj.com/site/about/guidelines.xhtml

Figure 1: Study timeline for participants

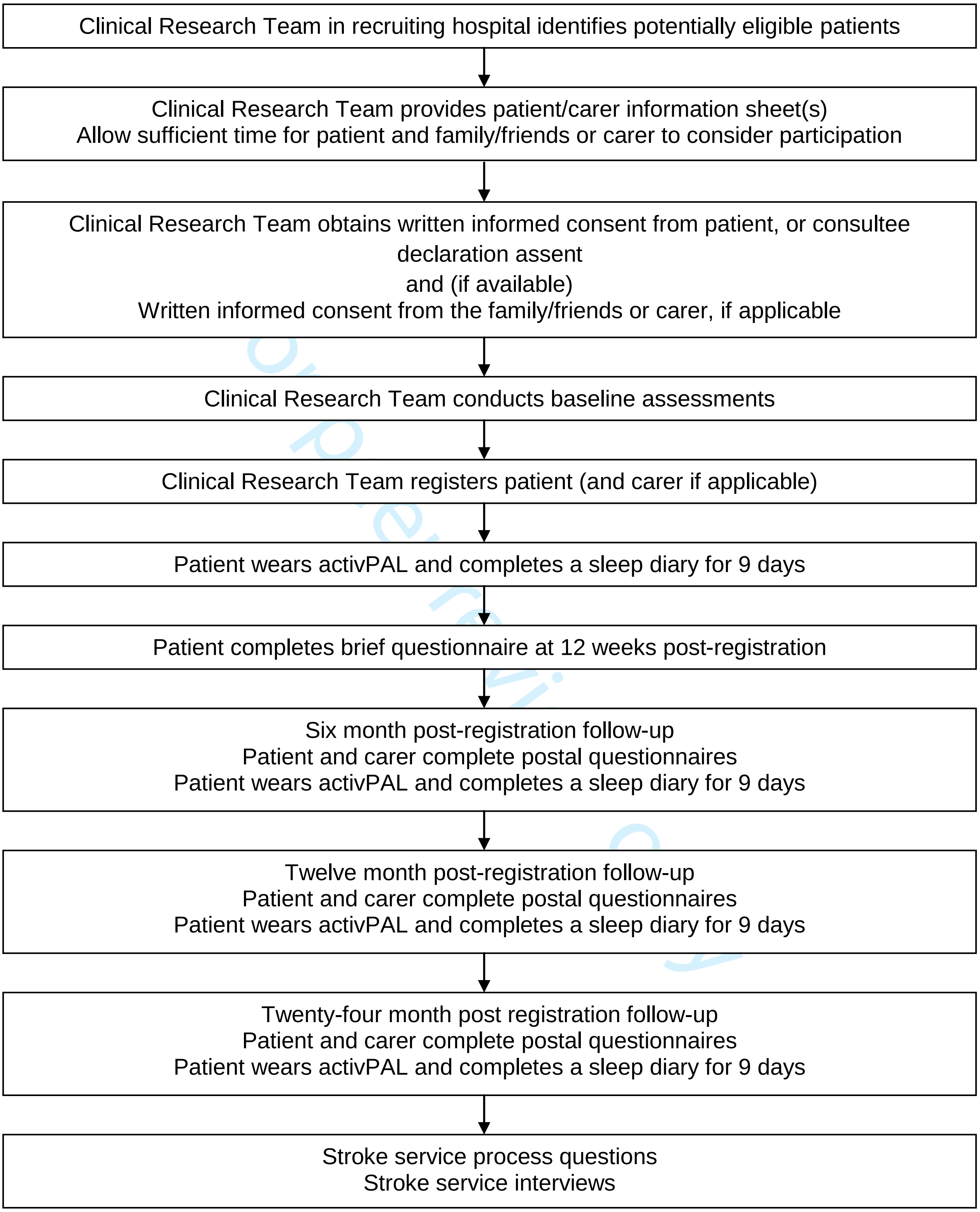

For peer review only - http://bmjopen.bmj.com/site/about/guidelines.xhtml

Appendix 1 – Participant outcome measures

Stroke survivors

- Nottingham Extended Activities of Daily Living scale (NEADL) (1): The NEADL assesses aspects of physical and social independence performance across 22 items (score range 0–66) grouped in four categories (mobility, kitchen, domestic and leisure activities), with higher scores indicating greater independence.
- World Health Organisation Disability Assessment Schedule 2.0 (12-item; WHODAS 2.0-short) (2-6). The WHODAS 2.0-short (2) was designed as a self-administered questionnaire which asks about difficulties due to health conditions. Items are scored on a 5-point Likert scale (from 0 (none) to 4 (extreme) and summed, then converted into a percentage, with higher scores indicating greater disability. A unit increment in WHODAS 2.0 z-score increased mortality hazard (6).
- Warwick-Edinburgh Mental Well-being Scale (WEMWBS) (7). The WEMWBS is designed for self-completion and comprises 14 items relating to an individuals' state of mental well-being in the previous two weeks. The scale is scored by summing responses to each item answered on a five-point Likert scale; total scores range from 14-70, with higher scores indicating higher levels of mental well-being.
- European Quality of Life 5-Dimension Health Questionnaire (5 levels; EQ-5D-5L) (8). The non-disease-specific EQ-5D-5L instrument will be used to measure health related quality of life across five dimensions: mobility, self-care, usual activities, pain/discomfort, and anxiety/depression. Each dimension is scored on a five-point scale, from 1 (no problems) to 5 (unable/ extreme problems); scores from the five dimensions are combined into a five-digit number to describe current health state. A visual analogue scale is also provided for participants to self-rate their health. The scale was developed to yield utility values which can be used to calculate QALY gains or losses, and thus will facilitate the health economic evaluation. Participants

For peer review only - http://bmjopen.bmj.com/site/about/guidelines.xhtml

are also asked to self-rate their health on the accompanying vertical Visual Analogue Scale, with the best and worst health they can imagine as endpoints.

- Fatigue Assessment Scale (FAS) (9, 10). The FAS is a 10-item questionnaire consisting of five questions relating to mental fatigue and five questions for physical fatigue. Participants are asked to rate how they usually feel on a five-point scale from 1 (never) to 5 (always), with total scores ranging from 10 to 50. Higher scores indicate increased fatigue.
- Measure of Older Adults' Sedentary Time (MOST) (11) and Sedentary Behaviour Visual Analogue Scale (adapted from (12)). The MOST asks participants to estimate the amount of time spent in seven sedentary activities during the previous week.
- Whether they have fallen and whether or not this led to injury (13). At baseline this will be related to in hospital falls and falls during the three months prior to admission
- Whether they use a walking aid
- Health and social care service use and informal care inputs

Carers

Modified Caregiver Strain Index (MCSI (14)). The MCSI is a 13-item measure of strain related to care provision covering five domains: financial; physical; psychological; social; and personal. Each item is scored 0 (no), 1 (sometimes) or 2 (yes), with total scores ranging from 0-26. Higher scores indicate higher levels of caregiver strain.

For peer review only - http://bmjopen.bmj.com/site/about/guidelines.xhtml

References

1. Nouri F, Lincoln N. An extended activities of daily living scale for stroke patients. Clinical rehabilitation. 1987;1(4):301-5.

2. Üstün TB, Chatterji S, Kostanjsek N, et al. Developing the World Health Organization disability assessment schedule 2.0. Bulletin of the World Health Organization. 2010;88:815-23.

3. Schlote A, Richter M, Wunderlich M, et al. WHODAS II with people after stroke and their relatives. Disability and rehabilitation. 2009;31(11):855-64.

4. Garin O, Ayuso-Mateos JL, Almansa J, et al. Validation of the" World Health Organization Disability Assessment Schedule, WHODAS-2" in patients with chronic diseases. Health and quality of life outcomes. 2010;8:1-15.

5. Küçükdeveci AA, Kutlay Ş, Yıldızlar D, et al. The reliability and validity of the World Health Organization Disability Assessment Schedule (WHODAS-II) in stroke. Disability and rehabilitation. 2013;35(3):214-20.

6. Hirve S, Juvekar S, Sambhudas S, et al. Does self-rated health predict death in adults aged 50 years and above in India? Evidence from a rural population under health and demographic surveillance. International journal of epidemiology. 2012;41(6):1719-27.

7. Tennant R, Hiller L, Fishwick R, et al. The Warwick-Edinburgh mental well-being scale (WEMWBS): development and UK validation. Health and Quality of life Outcomes. 2007;5(1):1-13.

8. Herdman M, Gudex C, Lloyd A, et al. Development and preliminary testing of the new five-level version of EQ-5D (EQ-5D-5L). Quality of life research. 2011;20:1727-36.

9. Mead G, Lynch J, Greig C, et al. Evaluation of fatigue scales in stroke patients. Stroke. 2007;38(7):2090-5.

10. Michielsen HJ, De Vries J, Van Heck GL. Psychometric qualities of a brief self-rated fatigue measure: The Fatigue Assessment Scale. Journal of psychosomatic research. 2003;54(4):345-52.

For peer review only

For peer review only - http://bmjopen.bmj.com/site/about/guidelines.xhtml

11. Gardiner PA, Eakin EG, Healy GN, et al. Feasibility of reducing older adults' sedentary time. American journal of preventive medicine. 2011;41(2):174-7.

12. Chastin SF, Dontje ML, Skelton DA, et al. Systematic comparative validation of self-report measures of sedentary time against an objective measure of postural sitting (activPAL). International Journal of Behavioral Nutrition and Physical Activity. 2018;15(1):1-12.

13. Perry L, Kendrick D, Morris R, et al. Completion and return of fall diaries varies with participants' level of education, first language, and baseline fall risk. Journals of Gerontology Series A: Biomedical Sciences and Medical Sciences. 2012;67(2):210-4.

14. Thornton M, Travis SS. Analysis of the reliability of the modified caregiver strain index. The Journals of Gerontology Series B: Psychological Sciences and Social Sciences. 2003;58(2):S127-S32.

For peer review only

For peer review only - http://bmjopen.bmj.com/site/about/guidelines.xhtml

STANDARD PROTOCOL ITEMS: RECOMMENDATIONS FOR INTERVENTIONAL TRIALS

SPIRIT 2013 Checklist: Recommended items to address in a clinical trial protocol and related documents*

Section/item	Item No	Description	Addressed on page number
**Administrative information**			
Title	1	Descriptive title identifying the study design, population, interventions, and, if applicable, trial acronym	Page 1
Trial registration	2a	Trial identifier and registry name. If not yet registered, name of intended registry	Page 2
	2b	All items from the World Health Organization Trial Registration Data Set	Throughout
Protocol version	3	Date and version identifier	Page 26
Funding	4	Sources and types of financial, material, and other support	Page 33
Roles and responsibilities	5a	Names, affiliations, and roles of protocol contributors	Page 1
	5b	Name and contact information for the trial sponsor	Page 33
	5c	Role of study sponsor and funders, if any, in study design; collection, management, analysis, and interpretation of data; writing of the report; and the decision to submit the report for publication, including whether they will have ultimate authority over any of these activities	Page 33
	5d	Composition, roles, and responsibilities of the coordinating centre, steering committee, endpoint adjudication committee, data management team, and other individuals or groups overseeing the trial, if applicable (see Item 21a for data monitoring committee)	Page 24 - 25
**Introduction**			
Background and rationale	6a	Description of research question and justification for undertaking the trial, including summary of relevant studies (published and unpublished) examining benefits and harms for each intervention	Page 3 - 5
	6b	Explanation for choice of comparators	Page 9
Objectives	7	Specific objectives or hypotheses	Page 5 - 6

For peer review only

Trial design	8	Description of trial design including type of trial (eg, parallel group, crossover, factorial, single group), allocation ratio, and framework (eg, superiority, equivalence, noninferiority, exploratory)	Page 6
**Methods: Participants, interventions, and outcomes**			
Study setting	9	Description of study settings (eg, community clinic, academic hospital) and list of countries where data will be collected. Reference to where list of study sites can be obtained	Page 7
Eligibility criteria	10	Inclusion and exclusion criteria for participants. If applicable, eligibility criteria for study centres and individuals who will perform the interventions (eg, surgeons, psychotherapists)	Page 7 - 8
Interventions	11a	Interventions for each group with sufficient detail to allow replication, including how and when they will be administered	Page 9 -10
	11b	Criteria for discontinuing or modifying allocated interventions for a given trial participant (eg, drug dose change in response to harms, participant request, or improving/worsening disease)	Page 10
	11c	Strategies to improve adherence to intervention protocols, and any procedures for monitoring adherence (eg, drug tablet return, laboratory tests)	Page 10 - 11
	11d	Relevant concomitant care and interventions that are permitted or prohibited during the trial	Page 9
Outcomes	12	Primary, secondary, and other outcomes, including the specific measurement variable (eg, systolic blood pressure), analysis metric (eg, change from baseline, final value, time to event), method of aggregation (eg, median, proportion), and time point for each outcome. Explanation of the clinical relevance of chosen efficacy and harm outcomes is strongly recommended	Page 11 - 15
Participant timeline	13	Time schedule of enrolment, interventions (including any run-ins and washouts), assessments, and visits for participants. A schematic diagram is highly recommended (see Figure)	Page 17 - 19
Sample size	14	Estimated number of participants needed to achieve study objectives and how it was determined, including clinical and statistical assumptions supporting any sample size calculations	Page 20
Recruitment	15	Strategies for achieving adequate participant enrolment to reach target sample size	Page 17 – 19
**Methods: Assignment of interventions (for controlled trials)**			
Allocation:			
Sequence generation	16a	Method of generating the allocation sequence (eg, computer-generated random numbers), and list of any factors for stratification. To reduce predictability of a random sequence, details of any planned restriction (eg, blocking) should be provided in a separate document that is unavailable to those who enrol participants or assign interventions	Page 15
Allocation concealment mechanism	16b	Mechanism of implementing the allocation sequence (eg, central telephone; sequentially numbered, opaque, sealed envelopes), describing any steps to conceal the sequence until interventions are assigned	Page 16

For peer review only

Implementation	16c	Who will generate the allocation sequence, who will enrol participants, and who will assign participants to interventions	Page 15
Blinding (masking)	17a	Who will be blinded after assignment to interventions (eg, trial participants, care providers, outcome assessors, data analysts), and how	Page 16
	17b	If blinded, circumstances under which unblinding is permissible, and procedure for revealing a participant's allocated intervention during the trial	n/a
**Methods: Data collection, management, and analysis**			
Data collection methods	18a	Plans for assessment and collection of outcome, baseline, and other trial data, including any related processes to promote data quality (eg, duplicate measurements, training of assessors) and a description of study instruments (eg, questionnaires, laboratory tests) along with their reliability and validity, if known. Reference to where data collection forms can be found, if not in the protocol	Page 11 – 15, 18 - 20 , Appendix 1
	18b	Plans to promote participant retention and complete follow-up, including list of any outcome data to be collected for participants who discontinue or deviate from intervention protocols	Page 19 - 20
Data management	19	Plans for data entry, coding, security, and storage, including any related processes to promote data quality (eg, double data entry; range checks for data values). Reference to where details of data management procedures can be found, if not in the protocol	Page 20
Statistical methods	20a	Statistical methods for analysing primary and secondary outcomes. Reference to where other details of the statistical analysis plan can be found, if not in the protocol	Page 20 - 23
	20b	Methods for any additional analyses (eg, subgroup and adjusted analyses)	Page 22
	20c	Definition of analysis population relating to protocol non-adherence (eg, as randomised analysis), and any statistical methods to handle missing data (eg, multiple imputation)	Page 20, 23
**Methods: Monitoring**			
Data monitoring	21a	Composition of data monitoring committee (DMC); summary of its role and reporting structure; statement of whether it is independent from the sponsor and competing interests; and reference to where further details about its charter can be found, if not in the protocol. Alternatively, an explanation of why a DMC is not needed	Page 24- 25
	21b	Description of any interim analyses and stopping guidelines, including who will have access to these interim results and make the final decision to terminate the trial	n/a - No formal interim or sub-group analyses are planned, and final analysis will take place when

For peer review only - http://bmjopen.bmj.com/site/about/guidelines.xhtml

			all available data have been received.
Harms	22	Plans for collecting, assessing, reporting, and managing solicited and spontaneously reported adverse events and other unintended effects of trial interventions or trial conduct	Page 25
Auditing	23	Frequency and procedures for auditing trial conduct, if any, and whether the process will be independent from investigators and the sponsor	Page 20, 24-25, 33
**Ethics and dissemination**			
Research ethics approval	24	Plans for seeking research ethics committee/institutional review board (REC/IRB) approval	Page 26
Protocol amendments	25	Plans for communicating important protocol modifications (eg, changes to eligibility criteria, outcomes, analyses) to relevant parties (eg, investigators, REC/IRBs, trial participants, trial registries, journals, regulators)	Page 26
Consent or assent	26a	Who will obtain informed consent or assent from potential trial participants or authorised surrogates, and how (see Item 32)	Page 17 -18
	26b	Additional consent provisions for collection and use of participant data and biological specimens in ancillary studies, if applicable	n/a
Confidentiality	27	How personal information about potential and enrolled participants will be collected, shared, and maintained in order to protect confidentiality before, during, and after the trial	Page 20
Declaration of interests	28	Financial and other competing interests for principal investigators for the overall trial and each study site	Page 34
Access to data	29	Statement of who will have access to the final trial dataset, and disclosure of contractual agreements that limit such access for investigators	Page 33 - 34
Ancillary and post-trial care	30	Provisions, if any, for ancillary and post-trial care, and for compensation to those who suffer harm from trial participation	n/a
Dissemination policy	31a	Plans for investigators and sponsor to communicate trial results to participants, healthcare professionals, the public, and other relevant groups (eg, via publication, reporting in results databases, or other data sharing arrangements), including any publication restrictions	Page 26
	31b	Authorship eligibility guidelines and any intended use of professional writers	Page 26
	31c	Plans, if any, for granting public access to the full protocol, participant-level dataset, and statistical code	Page 33 - 34
**Appendices**			
Informed consent	32	Model consent form and other related documentation given to participants and authorised surrogates	Not included -

For peer review only - http://bmjopen.bmj.com/site/about/guidelines.xhtml

materials			available on request
Biological specimens	33	Plans for collection, laboratory evaluation, and storage of biological specimens for genetic or molecular analysis in the current trial and for future use in ancillary studies, if applicable	n/a

*It is strongly recommended that this checklist be read in conjunction with the SPIRIT 2013 Explanation & Elaboration for important clarification on the items. Amendments to the protocol should be tracked and dated. The SPIRIT checklist is copyrighted by the SPIRIT Group under the Creative Commons "Attribution-NonCommercial-NoDerivs 3.0 Unported" license.

For peer review only

RECREATE: A study protocol for a multi-centre pilot cluster Randomised Controlled Trial (cRCT) in UK stroke services evaluating an intervention to reduce sedentary behaviour in stroke survivors (Get Set Go) with embedded process and economic evaluations

Journal:	*BMJ Open*
Manuscript ID	bmjopen-2023-074607.R2
Article Type:	Protocol
Date Submitted by the Author:	07-Jul-2023
Complete List of Authors:	Airlie, Jennifer; Bradford Institute for Health Research, Academic Unit for Ageing and Stroke Research<br>Burton, Louisa-Jane; Bradford Institute for Health Research, Academic Unit for Ageing and Stroke Research<br>Copsey, Bethan; University of Leeds, Clinical Trials Research Unit (CTRU)<br>English, Coralie; The University of Newcastle, School of Health Sciences; The University of Newcastle Hunter Medical Research Institute, Heart and Stroke Research Program<br>Farrin, Amanda; University of Leeds, Clinical Trials Research Unit (CTRU)<br>Fitzsimons, Claire F.; University of Edinburgh Institute for Sport Physical Education and Health Sciences, Physical Activity for Health and Research Centre<br>Holloway, Ivana; University Medical Center Göttingen, Institute of Medical Psychology and Medical Sociology<br>Horrocks, Judith; University of Leeds, Clinical Trials Research Unit (CTRU)<br>Johansson, Jessica; University of Leeds, Academic Unit for Ageing and Stroke Research<br>Mead, Gillian; University of Edinburgh, Centre for Clinical Brain Sciences<br>Moreau, Lauren; University of Leeds, Clinical Trials Research Unit (CTRU)<br>Ozer, Seline; Bradford Institute for Health Research, Academic Unit for Ageing and Stroke Research<br>Patel, Anita; Anita Patel Health Economics Consulting Ltd<br>Yaziji, Nahel; King's College London, Institute of Psychiatry, Psychology and Neuroscience<br>Forster, Anne; Bradford Institute for Health Research, Academic Unit for Ageing and Stroke Research; University of Leeds, Academic Unit for Ageing and Stroke Research
**Primary Subject Heading**:	Rehabilitation medicine
Secondary Subject Heading:	Health services research
Keywords:	Clinical Trial, REHABILITATION MEDICINE, Stroke < NEUROLOGY

Title: RECREATE: A study protocol for a multi-centre pilot cluster Randomised Controlled Trial (cRCT) in UK stroke services evaluating an intervention to reduce sedentary behaviour in stroke survivors (Get Set Go) with embedded process and economic evaluations

Jennifer Airlie[1], Louisa-Jane Burton[1], Bethan Copsey[2], Coralie English[3,4], Amanda Farrin[2], Claire Fitzsimons[5], Ivana Holloway[6], Judith Horrocks[2] , Jessica Faye Johansson[7], Gillian Mead[8], Lauren Moreau[2], Seline Ozer[1], Anita Patel[9], Nahel Yaziji[10], Anne Forster[1,7] on behalf of the RECREATE Programme Management Group

[1.] Academic Unit for Ageing and Stroke Research, Bradford Institute for Health Research, Bradford Teaching Hospitals NHS Foundation Trust, Bradford, UK

[2.] Clinical Trials Research Unit (CTRU), Leeds Institute of Clinical Trials Research, University of Leeds, Leeds, UK

[3.] School of Health Sciences, University of Newcastle, Newcastle, Australia.

[4.] Heart and Stroke Research Program, Hunter Medical Research Institute, Newcastle, Australia

[5.] Physical Activity for Health Research Centre, University of Edinburgh, Edinburgh, UK

[6.] Institute of Medical Psychology and Medical Sociology, University Medical Center Göttingen, Waldweg 37A, 37073, Göttingen, Germany

[7.] Academic Unit for Ageing and Stroke Research, Leeds Institute of Health Sciences, University of Leeds, Leeds, UK

[8.] Centre for Clinical Brain Sciences, University of Edinburgh, Edinburgh, UK

[9.] Anita Patel Health Economics Consulting Ltd, London, UK.

[10.] King's College London, Institute of Psychiatry Psychology and Neuroscience, London, UK.

Correspondence to Dr Jennifer Airlie, Academic Unit for Ageing and Stroke Research, Bradford Institute for Health Research, Bradford Teaching Hospitals NHS Foundation Trust, Bradford, BD9 6RJ, UK. Email: Jennifer.Airlie@bthft.nhs.uk

For peer review only - http://bmjopen.bmj.com/site/about/guidelines.xhtml

Abstract

Introduction: Sedentary behaviour (sitting or lying during waking hours without being otherwise active) is strongly associated with adverse health outcomes, including all-cause, cancer, and cardiovascular mortality in adults. Stroke survivors are consistently reported as being more sedentary than healthy age-matched controls, spending more hours sedentary daily and sustaining longer unbroken bouts of sedentary time. An evidence-based and clinically feasible intervention ('Get Set Go') was developed. A pragmatic definitive trial to evaluate Get Set Go was planned; however, due to the unprecedented effects of the COVID-19 pandemic on National Health Service (NHS) services this study was reduced in size and scope to become an external pilot trial. We report the protocol for this external pilot trial, which aims to undertake a preliminary exploration of whether Get Set Go is likely to improve ability to complete extended activities of daily living in the first year post-stroke and inform future trial designs in stroke rehabilitation.

Methods and analysis: This study is a pragmatic multi-centre, two-arm, external pilot cluster Randomised Controlled Trial (cRCT) with embedded process and economic evaluations. UK-based stroke services will be randomized 1:1 to the intervention (usual care plus Get Set Go) or control (usual care) arm. Fifteen stroke services will recruit 300-400 stroke in-patient and carer participants, with follow-up at six, 12 and 24 months. The proposed primary endpoint is stroke survivor self-reported Nottingham Extended Activities of Daily Living (NEADL) scale. Endpoint analyses will be exploratory and provide preliminary estimates of intervention effect. The process evaluation will provide valuable information on intervention fidelity, acceptability, and how it can be optimised.

Ethics and dissemination: The study has been approved by Yorkshire & The Humber-Bradford Leeds Research Ethics Committee (Ref: 19/YH/0403). Results will be disseminated through journal publications and conference presentations.

Trial registration: This trial was registered prospectively on 01/04/2020 (ISRCTN ref: 82280581).

For peer review only

For peer review only - http://bmjopen.bmj.com/site/about/guidelines.xhtml

Strengths and limitations of this study

- An external pilot cluster randomised control trial (cRCT) design enables the collection of feasibility and acceptability data to inform future trial designs in stroke rehabilitation.
- The comprehensive set of outcome measures collected will ensure any potential impacts of the intervention on meaningful outcomes for stroke survivors (e.g. short- and long-term changes to activities of daily living, quality of life and well-being) are captured.
- The study will collect objective measures of sedentary behaviour (using activity monitors), which to date have been infrequently assessed in post-stroke rehabilitation trials with longer term outcomes up to 24 months.
- The inclusion of an embedded process evaluation (reported separately) will provide valuable information on the intervention fidelity, acceptability, and how it can be optimised.
- The inclusion of a nested Study Within A Trial (SWAT) (reported separately) enables the systematic evaluation of whether a video animation could enhance participant understanding and subsequently uptake, engagement and compliance with the intervention and thus inform how best to implement the intervention in practice.

Introduction

Background and rationale

There are clear associations between sedentary behaviour in the general population and increased risk of adverse health outcomes, including all-cause, cancer, and cardiovascular mortality, and incidence of cardiovascular disease, cancer and Type II diabetes (1, 2). Sedentary behaviour is defined as any waking behaviour characterised by low energy expenditure ≤1.5 Metabolic Equivalent of Task (METs) while in a sitting, lying or reclining posture (3), and importantly differs from physical inactivity, which refers to achieving insufficient levels of moderate to vigorous physical activity (MVPA). An individual may therefore not reach the recommended levels of physical activity yet spend little time sitting, whereas others may be physically active for short

For peer review only - http://bmjopen.bmj.com/site/about/guidelines.xhtml

bursts (e.g., running for an hour), but spend prolonged periods sitting. Increasing MVPA can offset some of the detrimental effects of sedentary behaviour, although high levels are required (i.e., >300 minutes/ week) (1).

Stroke survivors are more sedentary than other population groups (4-6). Longitudinal studies (7); systematic reviews (8, 9) and observational studies (6) reported that stroke survivors are considerably more sedentary (in hours per day) and have longer unbroken bouts of sedentary time than healthy age-matched controls. Sedentary behaviour does not improve over the first year after stroke (7) and appears independent of the level of functional recovery (7, 8, 10), with physical ability only having a small influence on time spent sitting in people living at home six months after stroke (10). The high amount of reported sitting time by stroke survivors is likely to generate a range of negative outcomes. Epidemiological studies (11) place them in the highest quartile for cardiovascular risk. For a stroke survivor, increased sedentary behaviour may thus result in a “perfect storm” of deteriorating physical function and health-related quality of life, coincident with rising risks for cardiovascular disease.

There has been increasing focus in international public health guidance on reducing sedentary behaviour, particularly for those with disabilities, such as those resulting from stroke, as for many it may be a more achievable target than increasing PA (12). Short brief activity breaks throughout the day may be as effective as a continuous 30- or 60-minute bout of exercise (summarised in (13)). Experimental studies (primarily short-term, laboratory-based work) provide supporting evidence of the positive effect on metabolic outcomes of breaking up sitting time with short burst of activity in standing (14, 15) and reducing stroke risk (16).

Furthermore, the potential benefits of reducing sedentary behaviour reach beyond those relating to mortality and cardiovascular risk. Indeed, sedentary behaviour has been identified as a modifiable behaviour to enhance physical function (17) and maintain muscle strength, thus potentially supporting the ability to undertake activities of daily living (ADLs) (17-19). These benefits are important not just for stroke survivors themselves but may also extend to their carers; a recent

For peer review only

For peer review only - http://bmjopen.bmj.com/site/about/guidelines.xhtml

systematic review identified post-stroke disability as the biggest predictor of carer burden (20). Improved physical function in stroke survivors may thus reduce carer strain. Sedentary behaviour has also been identified as important in relation to mental health, cognitive outcomes, and health-related quality of life (21).

To address the challenge of reducing sedentary behaviour after stroke, we embarked upon a programme of work (RECREATE) to develop an evidence-based and clinically feasible intervention to reduce/ break up sedentary behaviour in stroke survivors. Intervention development was informed by systematic reviews and qualitative work, and underpinned by behaviour change theory (the Behaviour Change Wheel (22)). The final intervention ('Get Set Go') was developed using co-production methods (23). A logic model of intended intervention mechanisms and outcomes was also constructed, and implementation strategies were refined using a case study approach. The intervention is intended to begin early after stroke (in the in-patient setting) and continue into the community, and designed to fit into existing rehabilitation and recovery pathways.

This protocol represents the final stage in the RECREATE programme, which aims to evaluate the developed intervention in a sample of stroke survivors and carers. A large definitive multi-centre cluster Randomised Controlled Trial (cRCT) with 34 stroke services each recruiting 34 participants was initially planned, with the aim of evaluating the effectiveness and cost-effectiveness of the developed intervention. However, due to issues related to the worldwide COVID-19 pandemic, site recruitment was slower than expected. As a result, in Spring 2022, following meetings with the Programme Steering Committee (PSC) and a stakeholder meeting convened by the funder (National Institute for Health and care Research (NIHR)), a decision was taken with the funder to amend the protocol to reduce the trial in size and scope to become an external pilot trial.

Objectives

The primary objective is to undertake a preliminary exploration of whether the intervention (Get Set Go) is likely to improve the ability to complete Extended Activities of Daily Living (EADLs) in the

For peer review only - http://bmjopen.bmj.com/site/about/guidelines.xhtml

first year after stroke. A key secondary objective is to explore whether the intervention is likely to reduce sedentary behaviour. Other secondary objectives are to:

a) Explore core resource use and costs associated with delivering the intervention including impacts on wider care;
b) Explore whether the intervention reduces cardiovascular risk factors;
c) Explore whether the intervention reduces disability;
d) Explore whether the intervention improves health and wellbeing outcomes, such as health related quality of life and mental wellbeing;
e) Explore whether the intervention reduces carer strain;
f) Explore and understand implementation of the intervention, including intervention adherence, compliance, the process, benefits and challenges;
g) Explore and understand how the intervention is experienced and understood by recipients and providers;
h) Explore potential moderators and mediators of the intervention effect.

Objectives a-e will be addressed in the cRCT. Additional objectives addressed in the cRCT relate to gathering feasibility and acceptability data to inform future trial designs in stroke rehabilitation. Objectives f -h will be addressed in an embedded mixed-methods process evaluation (protocol reported separately), which will develop an understanding of how the intervention is implemented, understood and experienced by both providers and recipients.

Methods

Design

This trial is a pragmatic, multicentre, two-arm external pilot cRCT. The Get Set Go intervention will be delivered alongside usual care at the level of the stroke service (i.e., cluster). The cluster design will reduce between-group contamination. Eligible stroke services will be randomised on a 1:1 basis to the two arms of the trial: either intervention (usual care plus Get Set Go) or control (i.e., usual care). All stroke survivors in services allocated to the intervention arm will receive Get Set

For peer review only - http://bmjopen.bmj.com/site/about/guidelines.xhtml

Go; however outcomes will only be measured for those who consent to trial participation.

Settings

The trial will be conducted in up to 15 National Health Service (NHS) stroke services across the United Kingdom (UK). A stroke service is defined as an acute and/ or rehabilitation stroke unit with a linked community service over a defined geographical area. Service eligibility criteria are detailed in Table 1.

Table 1: Stroke service eligibility criteria

**Inclusion criteria**	**Exclusion criteria**
Both the inpatient and community service agree to participate	Previous participation in research leading to the development of the intervention
Availability of research staff to undertake participant recruitment	Currently implementing/ intending to implement similar interventions within trial duration
Agreement that recruitment targets are feasible/ acceptable (considering patient throughput)	Early Supported Discharge (ESD) (or Community if no ESD) waiting list >four weeks post-discharge
The intervention can be feasibly implemented (if so randomised)	

Recruitment

Three to four hundred stroke survivors and carers will be recruited to participate. Eligibility criteria are outlined in Table 2. Carer involvement is not a requirement for stroke survivor inclusion.

For peer review only - http://bmjopen.bmj.com/site/about/guidelines.xhtml

Table 2: Stroke survivor and carer eligibility criteria

	Inclusion criteria	Exclusion criteria
Stroke survivor	Aged ≥16 years at time of stroke	Receiving palliative care
	Clinical diagnosis of new or recurrent ischaemic or haemorrhagic (excluding subarachnoid haemorrhage) stroke	Due to be discharged outside the defined geographical area of the associated community service(s) participating in the trial.
	Requires manual contact of no more than one person to stand to prevent falling (manual contact consists of continuous or intermittent light touch to assist balance or co-ordination, i.e., not to support body weight)	
	Plan to live in the community post-discharge	
	Informed consent/ consultee declaration is provided	
Carer	Aged ≥16 years	Stroke survivor does not consent to participate
	Family member or friend regularly engaging with a stroke survivor participant (≥once per fortnight)	
	Provide informed consent	

Conversations with interested stroke services began in October 2019 in preparation for the study starting in December 2019. Recruitment of stroke survivors and carers started in January 2021. The study is projected to complete recruitment by the end of April 2023 with the follow-up due to

For peer review only

For peer review only - http://bmjopen.bmj.com/site/about/guidelines.xhtml

complete in April 2024. The end of the study is defined as the date the last participant's data item is collected.

Interventions

All stroke survivor participants (irrespective of randomisation allocation) will receive usual care within an organised stroke service. In line with the pragmatic nature of the trial, the comparator is 'usual care' as determined by local policy and practices, to draw comparison with current practice in NHS stroke services, especially given the variation in pathways. Information on the care delivered by both control and intervention sites will be captured by survey and enhanced by conversations with clinical staff (if further details are required) prior to randomisation and at regular intervals (approximately every three to six months) until the end of the trial. Observations conducted as part of the embedded process evaluation will provide additional insights into the nature of usual care provision at both control and intervention sites. This information will also provide context for trial implementation and monitor any potential contamination and/ or confounding between the two arms of the trial.

In stroke services allocated to the intervention arm, participants will also receive the Get Set Go intervention. Get Set Go is a whole-service intervention designed to be implemented and embedded within routine practice. Delivery commences in the in-patient stroke unit setting and continues into the community for at least 12 weeks post-discharge. The intervention focuses on:

a) Educating staff and stroke survivors (and their family/ friends/ carers where appropriate) about the importance of standing and moving after stroke;
b) Preparing and enabling staff to support and encourage stroke survivors to stand and move more in everyday stroke care (as part of routine practice);
c) Encouraging stroke survivors to monitor their own standing and moving, with assistance from family/ friends/ carers where appropriate.

As Get Set Go will be delivered at service level, all clinical staff members working within the stroke services randomised to deliver the intervention (i.e., inpatient and community settings) will be

For peer review only - http://bmjopen.bmj.com/site/about/guidelines.xhtml

invited to attend a training session (~one hour) to prepare for delivering Get Set Go. This will include the intervention rationale and an overview of key components. Staff will participate in practical tasks to ensure they feel confident in supporting and encouraging stroke survivors, who are capable of standing independently/ with the assistance of one person, to stand and move more in everyday stroke care (i.e., as part of routine clinical practice). A Template for Intervention Description and Replication (TIDieR) checklist (24) will be published with trial findings. There are no special criteria for discontinuing or modifying allocated interventions. Recommendations for standing and moving are made by staff as part of delivering the intervention alongside usual care in the stroke unit or community setting, based on their usual assessment techniques and clinical judgement. These recommendations will be regularly reviewed and modified in line with stroke survivors' individual capabilities and circumstances.

There are no provisions in place for post-trial care beyond the standard care provided within the participating NHS stroke services.

Study Within a Trial (SWAT)

This cRCT will also incorporate a SWAT (protocol reported separately (25), to evaluate whether a video animation could increase participant understanding of the intervention, and result in improved engagement and compliance, potentially resulting in improved outcomes. Intervention sites will be randomised 1:1 to the SWAT intervention (Get Set Go plus video animation) or control (Get Set Go with no video animation). Results will be reported separately.

Strategies to improve intervention adherence

The implementation team will maintain regular contact with clinical leads in sites allocated to the intervention arm to monitor delivery of the intervention and provide support as required. Clinical staff who are responsible for delivering the intervention will also complete monitoring records for all stroke survivors (whether or not they are trial participants) to document whether key tasks relating to the intervention have been completed (no personal data will be recorded for non-trial participants). It will be advised that a nominated member of staff checks these records have been

For peer review only - http://bmjopen.bmj.com/site/about/guidelines.xhtml
For peer review only

completed on a regular basis (e.g., towards the end of each shift). Regular review of these records will enable the study team to monitor intervention adherence and provide insight into intervention delivery. A short postal questionnaire completed by stroke survivor trial participants at 12-weeks post-hospital discharge will also assess intervention compliance, with those in the intervention arm responding to questions about the intervention; all participants will be asked about falls and use of a walking aid. Data on compliance will be analysed using descriptive summary statistics by allocation. Finally, intervention fidelity will be explored separately within the process evaluation, through site-based observations of intervention delivery, interviews with those delivering and receiving the intervention, and documentary analysis (including completed intervention materials).

Endpoints

The primary endpoint is stroke survivor self-reported Extended Activities of Daily Living (EADLs) at 12 months post-registration. The key secondary endpoint is mean daily sedentary time (minutes) at 12 months post-registration using activity monitor (ActivPAL) data, which provides a valid measure of posture and transitions in people with impaired mobility (26). Other secondary endpoints at six, 12- and 24- months post-registration (unless stated otherwise) for stroke survivor participants are listed below:

- Sedentary behaviour measured by activity monitor;
- EADLs (at six and 24 months only) measured by Nottingham Extended Activities of Daily Living scale (NEADL) (27);
- Health and disability measured by World Health Organisation Disability Assessment Schedule 2.0 (12-item; WHODAS 2.0-short) (28-32);
- Mental well-being measured by Warwick-Edinburgh Mental Well-being Scale (WEMWBS) (33);
- Health status measured by European Quality of Life 5-Dimension Health Questionnaire (5 levels; EQ-5D-5L) (34);
- Quality-Adjusted Life Year (QALY) gains (as above);
- Fatigue as measured by Fatigue Assessment Scale (FAS) (35, 36);

For peer review only - http://bmjopen.bmj.com/site/about/guidelines.xhtml
For peer review only

- Death;
- Falls and use of a walking aid (12-weeks post-discharge, and at six, 12 and 24 months);
- Self-reported sedentary behaviour measured by the Measure of Older Adults' Sedentary Time (MOST) (37) and a Sedentary Behaviour Visual Analogue Scale (adapted from (38)).
- Institutionalisation, hospital re-admission, and emergency department attendance rates;
- Cardiovascular risk markers: Body Mass Index (BMI), waist circumference, blood pressure (BP)
- Total major vascular events: Composite measure of non-fatal stroke, non-fatal myocardial infarction or death due to any vascular cause (including unexplained sudden death) (39);
- Self-reported health and social care service use and informal care inputs measured by a Client Services Receipt Inventory (CSRI) specifically adapted for this trial from versions used in previous stroke rehabilitation trials (40, 41).
- Costs from a health and social care perspective, and a societal perspective that further includes informal care.

The carer end-point is caregiver burden measured by Modified Caregiver Strain Index (MCSI (42)) at six, 12 and 24 months.

An overview of which assessments are undertaken at each time-point and the method of completion is provided in the Table 3. Information about the validity and reliability of the measures used is available in Appendix 1. Stroke survivors will also complete an additional brief postal questionnaire at 12-weeks post-discharge.

For peer review only

For peer review only - http://bmjopen.bmj.com/site/about/guidelines.xhtml

Table 3: Overview of assessments and method of completion at each study time-point

Outcome / Assessment	Method of completion	Time-point			
		Baseline	6 months post-registration	12 months post-registration	24 months post-registration
**Stroke Survivor**					
Demographic Details	Self-report	X			
Nottingham Extended Activities of Daily Living Scale (NEADL) (27)	Self-report	X	X	X	X
World Health Organisation Disability Assessment Scale (WHODAS) 2.0 12 Item (28-32)	Self-report	X	X	X	X
Warwick-Edinburgh Mental Well-being Scale (WEMWBS) (33)	Self-report	X	X	X	X
European Quality of Life 5-Dimension Health Questionnaire (EQ-5D-5L) (34)	Self-report	X	X	X	X
Fatigue Assessment Scale (FAS) (35, 36)	Self-report	X	X	X	X
Measure of Older Adults' Sedentary Time (MOST) (37)	Self-report	X	X	X	X
Sedentary Behaviour Visual Analogue Scale (adapted from	Self-report	X	X	X	X

For peer review only - http://bmjopen.bmj.com/site/about/guidelines.xhtml

(38))					
Use of a walking aid	Self-report	X	X	X	X
Falls question	Self-report	X	X	X	X
Hospital Admissions	Self-report / electronic health records		X	X	X
Client service receipt inventories (CSRI) to collect data on health and social care use and informal care inputs (adapted from (40, 41)	Self-report	X	X	X	X
Total major vascular events (39)	Electronic health records via routine data providers	X	X	X	X
Sedentary behaviour – activPAL measurements	Researcher administration / post	X	X	X	X
Risk markers for cardiovascular disease (Height* and weight for Body Mass Index (BMI); waist circumference; blood pressure (BP)) *collected at baseline only	Researcher collection from notes/ Researcher visit/ self-completion	X	X	X	X
Adverse Events (AE)	Self-report / Electronic		X	X	X

For peer review only - http://bmjopen.bmj.com/site/about/guidelines.xhtml

	health records				
Survival status			X	X	X
Admission to institution	Self-report		X	X	X
**Carer**					
Demographic Details	Self-report	X	(X)	(X)	(X)
Modified Caregiver Strain Index (MCSI) (42)	Self-report		X	X	X
Employment/ occupation status	Self-report	X	X	X	X

(X) only collected if 'new carer'

Randomisation and blinding

Site randomisation will be performed centrally by the Clinical Trials Research Unit (CTRU) and the allocation sequence will be computer-generated using a minimisation programme incorporating a random element to ensure the treatment arms are well-balanced. Eligible stroke services will be randomised on a 1:1 basis either to intervention or control, stratified by:

1. Sentinel Stroke National Audit Programme (SSNAP) grading level (43): A vs B/C/D/E (based on the latest available audit data at the time of randomisation: A=First class service, B=Good/ excellent in many aspects, C=Reasonable overall, some areas require improvement, D=Several areas require improvement, E=Substantial improvement required).

2. Single or combination of trusts: Single or combination.

3. Stroke unit size (acute): Median cut-off of ≤24 and >24 beds based on SSNAP acute organisational audit data (type 2 (solely for patients >72 hours post-stroke) and type 3 beds (used for both pre- and post-72-hour care) combined – based on the SSNAP audit data and confirmation by site).

For peer review only - http://bmjopen.bmj.com/site/about/guidelines.xhtml

Concealment of sequencing is ensured by the physically separate locations of the CTRU (where the randomisation outcome is stored) and the blinded study researchers. Following the randomisation, unblinded members of the study team, the Principal Investigator at the site, and clinical staff who will be involved in intervention delivery, will be informed of the randomisation outcome via secure email to facilitate organisation of intervention training in services allocated to this arm. Local researchers responsible for participant recruitment will be notified that randomisation has taken place but will remain blinded to randomisation outcome and will operate independently of the clinical staff involved in intervention delivery.

Local researchers involved in participant recruitment and in-person data collection (including at baseline, administering activity monitors and assessment of cardiovascular risk markers) will be blinded to the treatment allocation for their site. At the follow-up time-points, participant-completed outcome measures will be administered by CTRU staff via postal questionnaire, eliminating the potential for interviewer bias. The activity monitor (upon which data is recorded and stored until download) is sealed with no external indication of recording therefore it is not open to bias. Finally, data on mortality, re-admissions, and use of institutional care will be collected from independent sources and are unlikely to be subject to bias. Instances of unblinding will be recorded via staff self-report.

Participants, clinical staff involved in intervention delivery, and the statistical team analysing the outcome data, will not be blinded to treatment allocation. However, to minimise treatment bias, clinical staff involved in intervention delivery will not be overtly informed of which patients are participating in the trial. Additionally, to monitor for potential selection bias, key characteristics of included trial participants will be compared with publicly available datasets (e.g., SSNAP (43)) to monitor for evidence of selection bias.

Procedure

Eligible stroke survivors will be initially approached whilst they are an in-patient by a local researcher, who will provide verbal and written information about the trial before seeking informed

For peer review only

For peer review only - http://bmjopen.bmj.com/site/about/guidelines.xhtml

consent. Examples of an information sheet and informed consent form for stroke survivor participants are provided in Appendices 2 – 4.

To ensure that the trial population is representative of the clinical stroke population and inclusive of stroke survivors with cognitive impairment (including receptive, comprehension or language difficulties), recruitment procedures will include the use of accessible materials and procedures for consultee declaration (where appropriate), in compliance with the Mental Capacity Act 2005 (MCA) (44). As per the MCA, each stroke survivor will be assumed to have capacity unless it is established that they lack capacity. If there is any concern about capacity the local researcher will consult ward staff and/ or a family member/ carer (as appropriate) and a collective decision will be made as to whether the stroke survivor is deemed to have capacity to consent to participation in the trial. Changes in capacity will be monitored throughout the trial.

Potential stroke survivor participants who are deemed to have capacity to consent will be provided with written and verbal information about what their participation in the study would involve. Following time to consider participation (including discussion with family and/ or health professionals), those wishing to take part will provide informed consent through signing (or making a mark), or if they are unable, through witnessed verbal consent (by an independent observer). Should a potential participant be discharged prior to consent being gained and they consent to being contacted by the researcher, consent procedures will take place in their own home.

Should stroke survivors be deemed to lack capacity to provide informed consent, the researcher will seek to identify 'a supporting family member, friend or carer' (defined as someone who is in regular (at least weekly) contact with the stroke survivor), to support intervention delivery and outcome assessment. This individual will not necessarily be enrolled in the study themselves, unless they choose to enrol as a carer participant. If such a supporter is identified, the local researcher will either: act in accordance with an advance directive relative to research participation (if available) or seek advice from a personal consultee (through Consultee declaration). If a personal consultee is approached, they will be provided with an additional information sheet

For peer review only

For peer review only - http://bmjopen.bmj.com/site/about/guidelines.xhtml

outlining their role. Consultees will provide assent in person or via telephone (and signed by the local researcher, e.g., if the consultee is unable to visit the hospital prior to the stroke survivor's discharge). Carer participants will be approached by the local researcher once stroke survivors with capacity have given their verbal agreement or following provision of a consultee declaration for a stroke survivor participant who lacks capacity. Those wishing to participate will be asked to provide informed consent using the same procedure described above. Carers lacking the capacity to provide written informed consent will be excluded.

The local researcher will administer baseline questionnaires to stroke survivor and carer participants face-to-face and offer support where required. Proxy completion of questionnaires by a carer, family member or friend will be permitted (for those with and without capacity), with separate proxy questionnaires provided if required. Stroke survivors will also be asked to continuously wear an activPAL activity monitor on the thigh of their strongest/ unaffected leg for nine consecutive days, The monitor will be attached by the local researcher. They will also be asked to complete a purposely developed daily diary. This will capture if/ when the monitor has been removed; the time they went to bed and got up each day and whether they had episodes of sleep (i.e., naps) during the day. This information will assist with analysis and facilitate monitoring of adverse reactions to the materials used to attach the monitor. Local researchers will also collect data on risk markers for cardiovascular disease through face-to-face assessment or retrieval from medical records. These include height and weight (for calculation of BMI), waist circumference and blood pressure.

At the follow-up time points (six, 12- and 24-months post-registration), participant-completed outcome assessments will be administered by post in an assessment pack, including a covering letter and a pre-paid envelope for postal return. Activity monitors may be administered face-to-face by a local researcher or alternatively, be sent by post to the participant and returned from stroke survivor participants via pre-paid envelope. Cardiovascular risk markers (with the exception of height) will be collected alongside the activity monitor assessment. If it is not possible to organise a face-to-face visit, with the participants' agreement, options for self-assessment will be explored. Guidance will be provided to local researchers as to the action required if a blood pressure reading

For peer review only - http://bmjopen.bmj.com/site/about/guidelines.xhtml

is abnormal, including reporting this to a named clinician or notifying the participant's General Practitioner (GP). The participant will also be encouraged to discuss this with their GP.

At each participant follow-up, changes in circumstances, including change of address, institutionalisation, and death will be obtained from either recruiting teams, healthcare providers, participants, or their relatives. Additionally, for stroke survivors, the following data will be collected from electronic health records via routine data providers or directly from the recruiting hospital at each time-point: total major vascular events (composite of non-fatal stroke; non-fatal myocardial infarction; or death due to any vascular cause (including unexplained sudden death) (39); death; hospital admissions; emergency department attendance and institutionalisation. Stroke survivors will be made aware of the use of routine data providers (including NHS England, SystmOne, EMISWeb, and other relevant providers) at the time of study entry.

The study timeline for participants is presented in Figure 1.

[Figure 1 about here]

Initial letters, reminder letters and a participant pathway diagram will be distributed to participants to maximise data return at all time-points. The questionnaire sent to assess intervention compliance at 12-weeks post-hospital discharge will also serve as a reminder of study participation and act as a retention tool. At the follow-up time-points, reminders will be administered via telephone, text message, email, or post (according to participant preference) should the questionnaire not be returned within two weeks. If outcome assessments (with particular emphasis on the NEADL as the primary outcome), cannot be obtained by post, then (where appropriate) the assessments may be administered over the telephone or face-to-face to maximise return of data. Missing data, except individual data items collected via the postal assessment packs, will be sought until: it is received; confirmed as not available; or the trial is at analysis.

In instances where a participant withdraws from the trial clarification will be sought as to whether this is from all trial activities or individual components, e. g., wearing the activity monitor or

For peer review only - http://bmjopen.bmj.com/site/about/guidelines.xhtml

consenting to be contacted about participation in process evaluation activities. Unless participants explicitly instruct otherwise, electronic health record data will continue to be collected.

Data management

Data will be monitored for quality and completeness using established verification, validation and checking processes, e.g., double data entry, and securely stored. Received activPAL data will be subject to quality checks by researchers and where needed, additional details to aid interpretation will be sought from the recruiting site and/ or participant diaries. All data collected during the course of the trial will be kept strictly confidential using established processes. Data will be securely archived for ten years after the completion of the study and then destroyed.

Statistical analysis

All analyses and data summaries will be conducted on the intention-to-treat population (ITT) (45), defined as all participants registered to the trial, regardless of non-compliance with the protocol or withdrawal from the study or losses to follow-up. All participants recruited within a stroke service will be analysed according to the randomised allocation for that stroke service. No formal interim or sub-group analyses are planned, and final analysis will take place when all available data have been received.

Sample size

As previously described, our original plan for a large definitive multi-centre RCT was reduced in size and scope, due to issues related to the COVID-19 pandemic. We aim to recruit 300-400 participants across 15 stroke services. This revised sample size is based on the anticipated recruitment rate within the remaining time available in the funded programme, estimated at two participants per month per site. As intervention effectiveness will not be evaluated and all analyses will be of an exploratory nature, the study does not have a formal power calculation.

For peer review only - http://bmjopen.bmj.com/site/about/guidelines.xhtml

For peer review only

Primary endpoint analysis

In line with study objectives, all endpoint analyses will be exploratory and provide preliminary estimates of intervention effect. The primary outcome will be compared between randomised groups using multi-level mixed effects model (46) adjusting for design effects and other relevant known predictors of outcome, with participants nested within services, with services treated as random effects. If appropriate, missing data will be multiply imputed at the individual participant level. Parameter estimates will be reported with 95% confidence intervals. Sensitivity analyses of the primary endpoint will be conducted to assess the impact of missing data, the choice of imputation model and of assuming data are missing not at random, as appropriate. For the primary analysis, missing data will be assumed missing at random.

Incorporating the SWAT into the statistical analysis

For the primary analysis, the SWAT intervention will be accounted for in the regression model using an interaction term. Sensitivity analyses will explore the impact of the SWAT on the Get Set Go intervention effect, for example, analysing the outcomes as a 3-arm study separating the two SWAT arms. This will be described in further detail in the statistical analysis plan.

Secondary endpoints analyses

Secondary outcomes will be analysed in a similar manner to the primary outcome. For secondary outcomes, summary statistics will be presented for each time point by treatment group (means, standard deviations, medians, minimum, maximum, and quartiles for continuous variables, and counts and percentages for categorical variables). Secondary outcomes will be analysed using the same approach as the primary outcome with the relevant model for the type of outcome variable using multi-level linear or logistic regression, with multiple imputation for missing data where appropriate. Continuous distributions will be transformed where residuals are non-normal.

Self-reported falls, institutionalisation, re-admission, cardiovascular risk markers, total major vascular events and death rates between treatment arms will be summarised descriptively.

For peer review only - http://bmjopen.bmj.com/site/about/guidelines.xhtml

Quantitative summaries of intervention delivery will evaluate uptake of the intervention, adherence to the processes, staff and participant engagement and quality of intervention delivery, overall and by each site. Data on usual care will be summarised descriptively.

Activity monitor (activPAL) data

Sedentary time, as measured by activPAL activity monitors, will be analysed as total sedentary minutes on an average day as well as the percentage of sedentary time during wake time. Data from the monitors will be automatically recorded and processed using the activPAL software (PAL Technologies Ltd.). We will process data, remove sleep time and calculate, for each day, the total sedentary time; total sitting time accumulated in bouts ≥30 minutes, and bouts ≥ 60 minutes, number of sedentary bouts ≥30 minutes and bouts ≥ 60 minutes, total standing time, total stepping time and number of sit-to-stand transitions.

We will then calculate the key secondary outcome of mean daily sedentary time for the days when the monitor was worn. All other sedentary behaviour secondary outcomes will be calculated in the same way. The sedentary behaviour outcomes would ideally be based on seven days of continuous wear of the monitor. The calculation of sleep time, definition of a valid wear day and number of valid wear days required for inclusion in the analysis will be informed by the results of earlier feasibility work.

Further secondary analyses

Potential moderator variables: Potential predictors of response to the intervention will be explored via inclusion of key baseline stroke survivor characteristics (identified in our earlier work) as interaction effects in the primary analysis model, to understand who might benefit from the intervention.

Potential mediator variables will be explored by modelling the relationship between process variables (e.g., intervention adherence, number/ frequency of prompts and visits from staff, participant self-reported use of the guide, mediators such as fatigue or mood, etc.) and the primary

For peer review only - http://bmjopen.bmj.com/site/about/guidelines.xhtml
For peer review only

outcome.

Methods in analysis to handle protocol non-adherence and any statistical methods to handle missing data

All analyses and data summaries will be conducted on the ITT population. Missing data is anticipated at the item, scale and assessment levels. Missing data on key variables/ questionnaires will be explored. Missing data will be multiply imputed, where appropriate. Reasons for missing participant assessments will be explored and considered in the imputation process (e.g., died, moved away, etc.). If someone dies, they will be classed as lost to follow-up. When individual scales within the assessment questionnaire have missing items, scoring instructions specific to the scale will be followed, or if no such instructions exist, the summary scores will be calculated from the non-missing items using multiple imputation under the missing at random (MAR) mechanism, as long as at least 75% items are non-missing. We will test missing data assumptions in a sensitivity analysis.

Health economic analysis

The embedded health economic evaluation will assess resource use, costs and outcomes to inform data collection approaches for a future definitive trial, specifically:

- Alternative approaches to measuring hospital resource use (routine records versus self-report);
- A short (6-month) versus medium (12-month) versus longer (24-month) analytical time horizon; and
- A reduced list (narrow perspective) evaluation based on routine data versus long list (comprehensive perspective) evaluation incorporating self-reported data.

For peer review only - http://bmjopen.bmj.com/site/about/guidelines.xhtml
For peer review only

Two perspectives will be considered, as relevant to the analyses: health/social care (including institutionalisation), and a wider perspective covering health/social care (including institutionalisation) plus informal care given the significance of such inputs for this patient group.

Resource use data will be collected using a combination of NHS England secondary care data and self-report approaches via the CSRI. Resource use will be combined with relevant unit costs, using within-programme estimates related to the intervention, national estimates for other health and social care resources, and both an opportunity cost and replacement cost approach in turn for informal care. Total costs will be computed for each individual according to each of the estimation alternatives set out above. Differences between the two trial arms will be compared using bootstrap regression methods due to the expected skewness in data distributions.

Exploratory cost-effectiveness analyses will combine total costs with the primary outcome and with QALY gains estimated by attaching relevant general population utility weights to EQ-5D-5L health states at each time point. Uncertainty will be analysed using cost-effectiveness planes and cost-effectiveness acceptability curves (CEACs). Sensitivity analyses will explore the potential impact on cost-effectiveness from different implementation/costing scenarios. This would better inform future trial design and implementation discussions. Other relevant sensitivity analyses will be determined during the study and will be specified in the analysis plan prior to analyses.

The economic analyses will follow the same broad principles as the outcomes analyses, e.g. use of a signed off detailed analysis plan prior to analysis, adherence to ITT principles, imputation to account for missing values as appropriate, inclusion of baseline and other covariates for the comparisons, accounting for the clustering of patients within stroke services.

Oversight and monitoring

This trial is the final study in a seven-year Programme Grant for Applied Research funded by the NIHR. As such, a Programme Management Group (PMG), chaired by the Chief Investigator (CI) and attended by the programme co-applicants meets at least every four months, for strategic

For peer review only

For peer review only - http://bmjopen.bmj.com/site/about/guidelines.xhtml

oversight and management of the programme, and to monitor progress against key objectives. An independent Programme Steering Committee (PSC) chaired and attended by independent experts meets as a minimum every 12 months, and will provide external guidance and monitor progress. Finally, a Trial Management Group (TMG) comprised of the CI, key co-applicants, study researchers and key members of the project delivery team at the CTRU will be convened to meet every month to oversee day-to-day management of the trial. A Data Monitoring Committee is not required due to the lack of serious adverse events anticipated to be due to the intervention. The PSC will take on the role of monitoring safety concerns.

Adverse event reporting and harms

Adverse Events (AEs) such as falls and musculoskeletal injuries represent an inherent consequence of an active rehabilitation process and therefore cannot be entirely avoided. Similarly, in this patient population, acute illness resulting in hospitalisation, new medical problems and deterioration of existing medical problems are expected. In recognition of this, events fulfilling the definition of an AE or serious adverse events (SAE) will not be reportable in this study unless they result from administration of any research procedures and therefore fulfil the definition of an Unexpected and Related Serious AE, or if they constitute one of the following: death, hospital readmission, institutionalisation, treatment on an emergency out-patient basis or emergency department attendance. The latter will be reported at six-, 12- and 24- months post-registration follow-up by the clinical research team using health and social care records. All Related/ Unexpected Serious AEs occurring from the date of consent up to twenty-four months post registration must be reported within 24 hours of the clinical research staff becoming aware of the event. All Related/ Unexpected Serious AEs will be reviewed by the CI and subject to expedited reporting to the Sponsor and the main REC by the CTRU on behalf of the CI within 15 days.

Patient and Public Involvement (PPI)

PPI is central to this research. The research question was informed by initial discussion with our stroke survivor and carer group, who highlighted the need for interventions to encourage active

For peer review only - http://bmjopen.bmj.com/site/about/guidelines.xhtml

movement after stroke and contributed to grant development. PPI is subsequently being sought at every stage of the programme of research. Specific input from our PPI representatives includes: being actively involved in the design and management (e.g. steering/advisory group) of this research; assisting with developing participant information resources (including trial documents and intervention materials); contributing to ongoing reporting of the study and dissemination of emerging research findings.

Ethics and dissemination

The current protocol (v9.0 10/08/22) received ethical approval from the Yorkshire & The Humber – Bradford-Leeds REC (Ref: 19/YH/0403). Protocol amendments are processed in line with Health Research Authority and REC guidelines and processes. The results of the study will be published in peer-reviewed publications and will be presented at relevant national and international conferences. Authorship will be agreed in accordance with International Committee of Medical Journal Editors recommendations and the publication policy that has been agreed by the PMG for this programme of research. In collaboration with patient and public involvement representatives, lay reports to disseminate research findings to patient groups and clinical staff at participating sites will also be compiled.

For peer review only

For peer review only - http://bmjopen.bmj.com/site/about/guidelines.xhtml

References

1. Dempsey PC, Biddle SJ, Buman MP, et al. New global guidelines on sedentary behaviour and health for adults: broadening the behavioural targets. International Journal of Behavioral Nutrition and Physical Activity. 2020;17(1):1-12.

2. U.S. Department of Health and Human Services. Physical Activity Guidelines for Americans, 2nd edition. Washington, DC: U.S. Department of Health and Human Services; 2018.

3. Tremblay MS, Aubert S, Barnes JD, et al. Sedentary behavior research network (SBRN)–terminology consensus project process and outcome. International journal of behavioral nutrition and physical activity. 2017;14:1-17.

4. Butler EN, Evenson KR. Prevalence of physical activity and sedentary behavior among stroke survivors in the United States. Topics in stroke rehabilitation. 2014;21(3):246-55.

5. Paul L, Brewster S, Wyke S, et al. Physical activity profiles and sedentary behaviour in people following stroke: a cross-sectional study. Disability and rehabilitation. 2016;38(4):362-7.

6. English C, Healy GN, Coates A, et al. Sitting and activity time in people with stroke. Physical therapy. 2016;96(2):193-201.

7. Tieges Z, Mead G, Allerhand M, et al. Sedentary behavior in the first year after stroke: a longitudinal cohort study with objective measures. Archives of physical medicine and rehabilitation. 2015;96(1):15-23.

8. English C, Manns PJ, Tucak C, et al. Physical activity and sedentary behaviors in people with stroke living in the community: a systematic review. Physical therapy. 2014;94(2):185-96.

9. Galea SL, Lee M-J, English C, et al. Sedentary versus active behavior in people after stroke. Physical Therapy Reviews. 2015;20(1):1-7.

10. English C, Healy GN, Coates A, et al. Sitting time and physical activity after stroke: physical ability is only part of the story. Topics in stroke rehabilitation. 2016;23(1):36-42.

11. Wullems JA, Verschueren SM, Degens H, et al. A review of the assessment and prevalence of sedentarism in older adults, its physiology/health impact and non-exercise mobility counter-measures. Biogerontology. 2016;17:547-65.

For peer review only

For peer review only - http://bmjopen.bmj.com/site/about/guidelines.xhtml

12. World Health Organization. WHO guidelines on physical activity and sedentary behaviour. 2020.

13. Dempsey PC, Owen N, Biddle SJ, et al. Managing sedentary behavior to reduce the risk of diabetes and cardiovascular disease. Current diabetes reports. 2014;14:1-11.

14. Benatti FB, Ried-Larsen M. The effects of breaking up prolonged sitting time: a review of experimental studies. Medicine & Science in Sports & Exercise. 2015;47(10):2053-61.

15. English C, Janssen H, Crowfoot G, et al. Frequent, short bouts of light-intensity exercises while standing decreases systolic blood pressure: Breaking Up Sitting Time after Stroke (BUST-Stroke) trial. International journal of stroke. 2018;13(9):932-40.

16. Mackie P, Weerasekara I, Crowfoot G, et al. What is the effect of interrupting prolonged sitting with frequent bouts of physical activity or standing on first or recurrent stroke risk factors? A scoping review. PloS one. 2019;14(6):e0217981.

17. Rosenberg DE, Bellettiere J, Gardiner PA, et al. Independent associations between sedentary behaviors and mental, cognitive, physical, and functional health among older adults in retirement communities. Journals of gerontology series a: Biomedical sciences and medical sciences. 2016;71(1):78-83.

18. Ezeugwu VE, Manns PJ. The feasibility and longitudinal effects of a home-based sedentary behavior change intervention after stroke. Archives of Physical Medicine and Rehabilitation. 2018;99(12):2540-7.

19. Amaral Gomes ES, Ramsey KA, Rojer AG, et al. The association of objectively measured physical activity and sedentary behavior with (instrumental) activities of daily living in community-dwelling older adults: a systematic review. Clinical interventions in aging. 2021:1877-915.

20. Shamoun C, Harris MA. Impact of stroke survivors' physical disability on the presence of caregiver burden: a systematic review. Journal of Neuroscience Nursing. 2022;54(4):146-50.

21. Saunders DH, Mead GE, Fitzsimons C, et al. Interventions for reducing sedentary behaviour in people with stroke. Cochrane Database of Systematic Reviews. 2021(6).

For peer review only

For peer review only - http://bmjopen.bmj.com/site/about/guidelines.xhtml

22. Michie S, Van Stralen MM, West R. The behaviour change wheel: a new method for characterising and designing behaviour change interventions. Implementation science. 2011;6(1):1-12.

23. Hall J, Morton S, Hall J, et al. A co-production approach guided by the behaviour change wheel to develop an intervention for reducing sedentary behaviour after stroke. Pilot and Feasibility Studies. 2020;6(1):1-13.

24. Hoffmann TC, Glasziou PP, Boutron I, et al. Better reporting of interventions: template for intervention description and replication (TIDieR) checklist and guide. BMJ. 2014;348.

25. Ahmed S, Airlie J, Clegg A, et al. A new opportunity for enhancing trial efficiency: Can we investigate intervention implementation processes within trials using SWAT (study within a trial) methodology? Research Methods in Medicine & Health Sciences. 2022;3(3):66-73.

26. Taraldsen K, Askim T, Sletvold O, et al. Evaluation of a body-worn sensor system to measure physical activity in older people with impaired function. Physical therapy. 2011;91(2):277-85.

27. Nouri F, Lincoln N. An extended activities of daily living scale for stroke patients. Clinical rehabilitation. 1987;1(4):301-5.

28. Üstün TB, Chatterji S, Kostanjsek N, et al. Developing the World Health Organization disability assessment schedule 2.0. Bulletin of the World Health Organization. 2010;88:815-23.

29. Schlote A, Richter M, Wunderlich M, et al. WHODAS II with people after stroke and their relatives. Disability and rehabilitation. 2009;31(11):855-64.

30. Garin O, Ayuso-Mateos JL, Almansa J, et al. Validation of the "World Health Organization Disability Assessment Schedule, WHODAS-2" in patients with chronic diseases. Health and quality of life outcomes. 2010;8:1-15.

31. Küçükdeveci AA, Kutlay Ş, Yıldızlar D, et al. The reliability and validity of the World Health Organization Disability Assessment Schedule (WHODAS-II) in stroke. Disability and rehabilitation. 2013;35(3):214-20.

For peer review only

For peer review only - http://bmjopen.bmj.com/site/about/guidelines.xhtml

32. Hirve S, Juvekar S, Sambhudas S, et al. Does self-rated health predict death in adults aged 50 years and above in India? Evidence from a rural population under health and demographic surveillance. International journal of epidemiology. 2012;41(6):1719-27.

33. Tennant R, Hiller L, Fishwick R, et al. The Warwick-Edinburgh mental well-being scale (WEMWBS): development and UK validation. Health and Quality of life Outcomes. 2007;5(1):1-13.

34. Herdman M, Gudex C, Lloyd A, et al. Development and preliminary testing of the new five-level version of EQ-5D (EQ-5D-5L). Quality of life research. 2011;20:1727-36.

35. Mead G, Lynch J, Greig C, et al. Evaluation of fatigue scales in stroke patients. Stroke. 2007;38(7):2090-5.

36. Michielsen HJ, De Vries J, Van Heck GL. Psychometric qualities of a brief self-rated fatigue measure: The Fatigue Assessment Scale. Journal of psychosomatic research. 2003;54(4):345-52.

37. Gardiner PA, Eakin EG, Healy GN, et al. Feasibility of reducing older adults' sedentary time. American journal of preventive medicine. 2011;41(2):174-7.

38. Chastin SF, Dontje ML, Skelton DA, et al. Systematic comparative validation of self-report measures of sedentary time against an objective measure of postural sitting (activPAL). International Journal of Behavioral Nutrition and Physical Activity. 2018;15(1):1-12.

39. PROGRESS Collaborative Group. Randomised trial of a perindopril-based blood-pressure-lowering regimen among 6105 individuals with previous stroke or transient ischaemic attack. The Lancet. 2001;358(9287):1033-41.

40. Forster A, Dickerson J, Young J, et al. A structured training programme for caregivers of inpatients after stroke (TRACS): a cluster randomised controlled trial and cost-effectiveness analysis. The Lancet. 2013;382(9910):2069-76.

41. Forster A, Ozer S, Crocker TF, et al. Longer-term health and social care strategies for stroke survivors and their carers: the LoTS2Care research programme including cluster feasibility RCT. Programme Grants Appl Res 2021;9(3).

42. Thornton M, Travis SS. Analysis of the reliability of the modified caregiver strain index. The Journals of Gerontology Series B: Psychological Sciences and Social Sciences. 2003;58(2):S127-S32.

For peer review only

For peer review only - http://bmjopen.bmj.com/site/about/guidelines.xhtml

43. Sentinel Stroke National Audit Programme (SSNAP). Annual Public Report National Results2019. Available from: https://www.strokeaudit.org/Documents/National/Clinical/Apr2017Mar2018/Apr2017Mar2018-AnnualReport.aspx (Accessed 13 March 2023).

44. Mental Capacity Act [Internet]. The Stationary Office. 2005. Available from: https://www.legislation.gov.uk/ukpga/2005/9/contents (accessed March 2023).

45. Altman DG. Practical statistics for medical research. Boca Raton, Florida: Chapman & Hall/CRC; 1999.

46. Hayes RJ, Moulton LH. Cluster randomised trials, 2nd Ed. Boca Raton: CRC Press; 2017.

For peer review only

For peer review only - http://bmjopen.bmj.com/site/about/guidelines.xhtml

Authors' contributions: AF is lead grant holder and Chief Investigator and will oversee the design and implementation of the trial. CE, CF and GM, are coinvestigators who have contributed to development of the protocol, and attend regular programme meetings, providing advice as needed. AFa, assisted by IH and BC, led the statistical and methodological design of the trial and is the statistical guarantor responsible for the main statistical analysis and reporting. AP leads the design of the health economic components of the trial and is responsible for the health economic analyses, assisted by NY. LM, assisted by JH, leads on data management and supports the day-to-day operation of the trial. SO, assisted by JA, is responsible for managing implementation of the intervention and delivery of the trial. LJB also assisted with managing delivery of the trial. JFJ leads the embedded process evaluation. JA leads on activPAL data implementation and data issues. JA and LJB drafted the manuscript. All authors read and approved the final manuscript. This paper is written on behalf of the RECREATE Programme Management Group who meet regularly to oversee the programme of research. All authors reviewed and approved the final manuscript.

Acknowledgements: The authors would like to acknowledge the support of the Clinical Research Network (CRN) and also express our appreciation for all of the hard work and support provided by the clinical teams who are helping to deliver this trial. The authors would also like to thank members of the Programme Steering Committee for their helpful contributions through the RECREATE programme of research.

Finally the authors would like to acknowledge the expert support of the RECREATE Programme Group who meet regularly to oversee the programme of research. The group is comprised of: Jennifer Airlie, Karen Birch, Gillian Carter, Bethan Copsey, Florence Day, Coralie English, Amanda Farrin, Alison Fergusson, Claire Fitzsimons, Anne Forster, Jessica Faye Johansson, Rebecca Lawton, Laura Marsden, Gillian Mead, Lauren Moreau, Seline Ozer, Anita Patel, Rosemary Shannon, Nahel Yaziji.

For peer review only

For peer review only - http://bmjopen.bmj.com/site/about/guidelines.xhtml

Funding statement: This work was supported by the National Institute for Health and Care Research (NIHR) under its Programme Grants for Applied Research Programme, grant number RP-PG-0615-20019. The views expressed are those of the author(s) and not necessarily those of the NIHR or the Department of Health and Social Care. The Funder has had no role in trial design, beyond setting the research question the trial addresses. Data collection, management, analysis and interpretation will remain independent of the Funder.

Sponsor: The trial sponsor is Bradford Teaching Hospitals NHS Foundation Trust, Research Management & Support Office, Bradford Institute for Health Research, Bradford, BD9 6RJ, United Kingdom. Tel: +44 (0)1274 38 2575; e-mail: jane.dennison@bthft.nhs.uk

The Sponsor will maintain oversight of trial processes, but is not involved in trial design or delivery processes. The Sponsor will not participate in data analysis or trial reporting processes.

Data sharing statement: De-identified individual participant data datasets generated and/or analysed during the current study will be available upon request from the Clinical Trials Research Unit, University of Leeds (contact CTRU-DataAccess@leeds.ac.uk in the first instance). Data will be made available at the end of the trial, i.e. usually when all primary and secondary endpoints have been met and all key analyses are complete. Data will remain available from then on for as long as CTRU retains the data.

CTRU makes data available by a 'controlled access' approach. Data will only be released for legitimate secondary research purposes, where the Chief Investigator, Sponsor and CTRU agree that the proposed use has scientific value and will be carried out to a high standard (in terms of scientific rigour and information governance and security) and that there are resources available to satisfy the request. Data will only be released in line with participants' consent, all applicable laws relating to data protection and confidentiality, and any contractual obligations to which the CTRU is subject. No individual participant data will be released before an appropriate agreement is in place

For peer review only - http://bmjopen.bmj.com/site/about/guidelines.xhtml

setting out the conditions of release. The agreement will govern data retention, usually stipulating that data recipients must delete their copy of the released data at the end of the planned project.

Competing interests statement:

AF, AFa, CE, CF, IH, GM and AP are coinvestigators on the grant funding this work therefore are partially supported by the National Institute for Health and Social Care Research (NIHR) (grant number RP-PG-0615-20019).

AF has received additional research grant support from NIHR through the following funding streams: Senior Investigator award, Health Technology Assessment (HTA) and Health and Social Care Delivery Research (HS&DR). AF has previously received support from the Stroke Association to attend the UK stroke forum and received payment from the National Institute for Health (USA) for panel membership (2021, 2022). AF is currently the chair / a member of programme steering committees for NIHR research programmes (Grant reference numbers: NIHR 202339, NIHR 202020) and has served on the following panels: NIHR Doctoral Fellowships, NIHR senior investigators committee (2019/20), NIHR HS&DR committee (2016-2018) and Stroke Association Funding.

CE has received grant funding from the Netherlands Organisation for Scientific Research (NOW) Taskforce for Applied Research (SIA RAAK) for work in a similar area (i.e. sitting less and moving more after stroke) and is a Non-executive Director representing interests of Research and Chair of Research Advisory Committee for the Stroke Foundation of Australia (unpaid).

CF is a coinvestigator / collaborator on other grants on the topic of sedentary behaviour / physical activity and is therefore partially supported by grant funding received from the University of Edinburgh and the Irish Health Board. CE has previously been supported to conduct work in a similar area by grant funding received from the Chief Scientist Office of the Scottish Government, Medical Research Council (MRC) Public Health Intervention Development (PHIND) award and the University of Edinburgh.

JA, LJB, BC, JH, JFJ, LM, SO and NY report no competing interests related to the manuscript.

For peer review only - http://bmjopen.bmj.com/site/about/guidelines.xhtml

Patient and public involvement: Patients and/or the public were involved in the design, conduct, reporting and dissemination plans of this research. Please refer to the methods section for further details.

Twitter: @RECREATE_stroke

Figure 1 legend: Study timeline for participants

For peer review only

For peer review only - http://bmjopen.bmj.com/site/about/guidelines.xhtml

Figure 1: Study timeline for participants

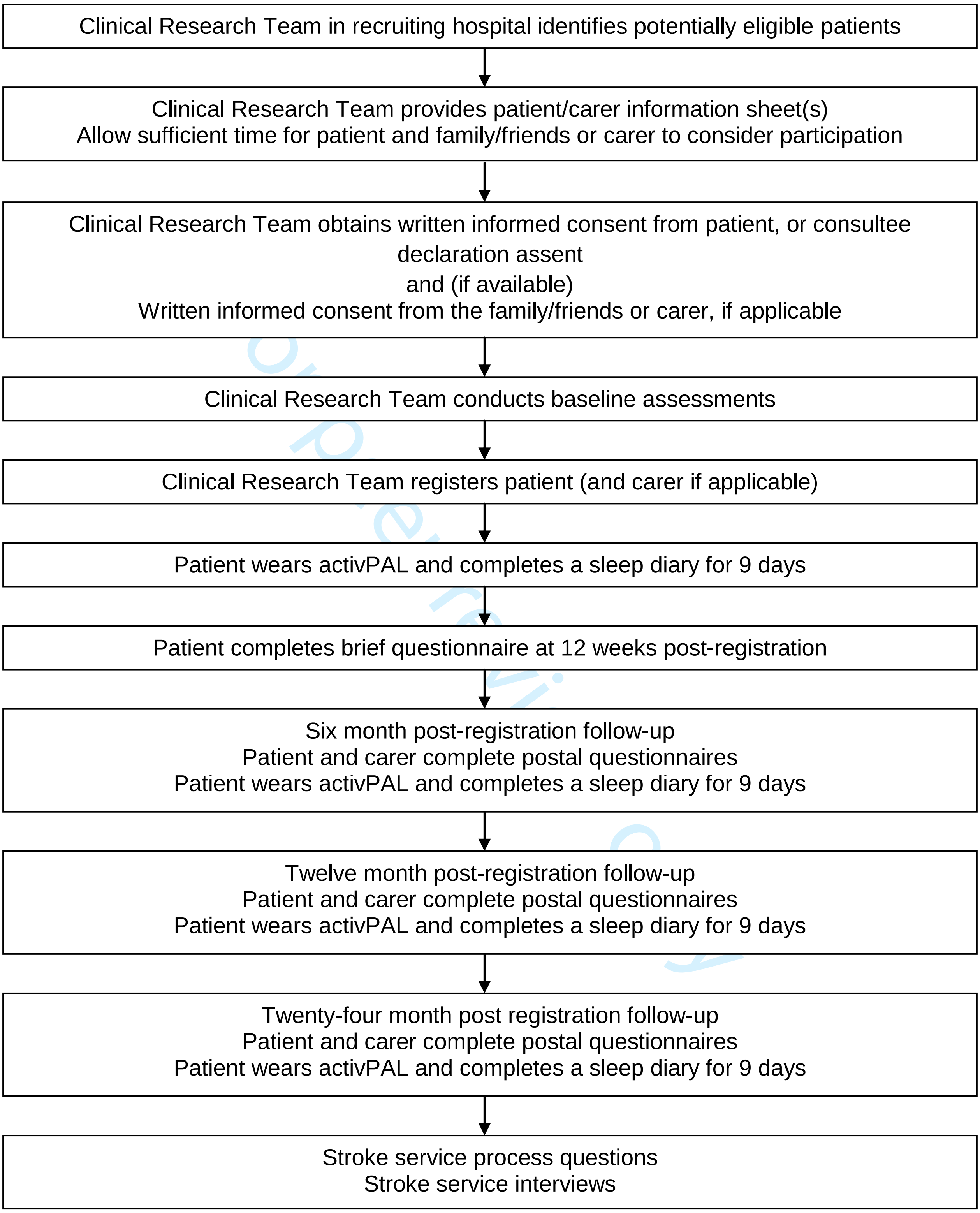

Appendix 1 – Participant outcome measures

Stroke survivors

- Nottingham Extended Activities of Daily Living scale (NEADL) (1): The NEADL assesses aspects of physical and social independence performance across 22 items (score range 0–66) grouped in four categories (mobility, kitchen, domestic and leisure activities), with higher scores indicating greater independence.
- World Health Organisation Disability Assessment Schedule 2.0 (12-item; WHODAS 2.0-short) (2-6). The WHODAS 2.0-short (2) was designed as a self-administered questionnaire which asks about difficulties due to health conditions. Items are scored on a 5-point Likert scale (from 0 (none) to 4 (extreme) and summed, then converted into a percentage, with higher scores indicating greater disability. A unit increment in WHODAS 2.0 z-score increased mortality hazard (6).
- Warwick-Edinburgh Mental Well-being Scale (WEMWBS) (7). The WEMWBS is designed for self-completion and comprises 14 items relating to an individuals' state of mental well-being in the previous two weeks. The scale is scored by summing responses to each item answered on a five-point Likert scale; total scores range from 14-70, with higher scores indicating higher levels of mental well-being.
- European Quality of Life 5-Dimension Health Questionnaire (5 levels; EQ-5D-5L) (8). The non-disease-specific EQ-5D-5L instrument will be used to measure health related quality of life across five dimensions: mobility, self-care, usual activities, pain/discomfort, and anxiety/depression. Each dimension is scored on a five-point scale, from 1 (no problems) to 5 (unable/ extreme problems); scores from the five dimensions are combined into a five-digit number to describe current health state. A visual analogue scale is also provided for participants to self-rate their health. The scale was developed to yield utility values which can be used to calculate QALY gains or losses, and thus will facilitate the health economic evaluation. Participants

For peer review only - http://bmjopen.bmj.com/site/about/guidelines.xhtml

are also asked to self-rate their health on the accompanying vertical Visual Analogue Scale, with the best and worst health they can imagine as endpoints.

- Fatigue Assessment Scale (FAS) (9, 10). The FAS is a 10-item questionnaire consisting of five questions relating to mental fatigue and five questions for physical fatigue. Participants are asked to rate how they usually feel on a five-point scale from 1 (never) to 5 (always), with total scores ranging from 10 to 50. Higher scores indicate increased fatigue.
- Measure of Older Adults' Sedentary Time (MOST) (11) and Sedentary Behaviour Visual Analogue Scale (adapted from (12)). The MOST asks participants to estimate the amount of time spent in seven sedentary activities during the previous week.
- Whether they have fallen and whether or not this led to injury (13). At baseline this will be related to in hospital falls and falls during the three months prior to admission
- Whether they use a walking aid
- Health and social care service use and informal care inputs

Carers

Modified Caregiver Strain Index (MCSI (14)). The MCSI is a 13-item measure of strain related to care provision covering five domains: financial; physical; psychological; social; and personal. Each item is scored 0 (no), 1 (sometimes) or 2 (yes), with total scores ranging from 0-26. Higher scores indicate higher levels of caregiver strain.

For peer review only - http://bmjopen.bmj.com/site/about/guidelines.xhtml

References

1. Nouri F, Lincoln N. An extended activities of daily living scale for stroke patients. Clinical rehabilitation. 1987;1(4):301-5.

2. Üstün TB, Chatterji S, Kostanjsek N, et al. Developing the World Health Organization disability assessment schedule 2.0. Bulletin of the World Health Organization. 2010;88:815-23.

3. Schlote A, Richter M, Wunderlich M, et al. WHODAS II with people after stroke and their relatives. Disability and rehabilitation. 2009;31(11):855-64.

4. Garin O, Ayuso-Mateos JL, Almansa J, et al. Validation of the" World Health Organization Disability Assessment Schedule, WHODAS-2" in patients with chronic diseases. Health and quality of life outcomes. 2010;8:1-15.

5. Küçükdeveci AA, Kutlay Ş, Yıldızlar D, et al. The reliability and validity of the World Health Organization Disability Assessment Schedule (WHODAS-II) in stroke. Disability and rehabilitation. 2013;35(3):214-20.

6. Hirve S, Juvekar S, Sambhudas S, et al. Does self-rated health predict death in adults aged 50 years and above in India? Evidence from a rural population under health and demographic surveillance. International journal of epidemiology. 2012;41(6):1719-27.

7. Tennant R, Hiller L, Fishwick R, et al. The Warwick-Edinburgh mental well-being scale (WEMWBS): development and UK validation. Health and Quality of life Outcomes. 2007;5(1):1-13.

8. Herdman M, Gudex C, Lloyd A, et al. Development and preliminary testing of the new five-level version of EQ-5D (EQ-5D-5L). Quality of life research. 2011;20:1727-36.

9. Mead G, Lynch J, Greig C, et al. Evaluation of fatigue scales in stroke patients. Stroke. 2007;38(7):2090-5.

10. Michielsen HJ, De Vries J, Van Heck GL. Psychometric qualities of a brief self-rated fatigue measure: The Fatigue Assessment Scale. Journal of psychosomatic research. 2003;54(4):345-52.

For peer review only - http://bmjopen.bmj.com/site/about/guidelines.xhtml

11. Gardiner PA, Eakin EG, Healy GN, et al. Feasibility of reducing older adults' sedentary time. American journal of preventive medicine. 2011;41(2):174-7.

12. Chastin SF, Dontje ML, Skelton DA, et al. Systematic comparative validation of self-report measures of sedentary time against an objective measure of postural sitting (activPAL). International Journal of Behavioral Nutrition and Physical Activity. 2018;15(1):1-12.

13. Perry L, Kendrick D, Morris R, et al. Completion and return of fall diaries varies with participants' level of education, first language, and baseline fall risk. Journals of Gerontology Series A: Biomedical Sciences and Medical Sciences. 2012;67(2):210-4.

14. Thornton M, Travis SS. Analysis of the reliability of the modified caregiver strain index. The Journals of Gerontology Series B: Psychological Sciences and Social Sciences. 2003;58(2):S127-S32.

For peer review only

For peer review only - http://bmjopen.bmj.com/site/about/guidelines.xhtml

Appendix 2 – Information sheet for stroke survivors

[INSERT LOGOS HERE]

RECREATE
Reducing sedentary behaviour after stroke

INFORMATION SHEET FOR STROKE SURVIVORS

Helping people sit less after stroke –

COMPLETE QUESTIONNAIRES & WEAR AN ACTIVITY MONITOR

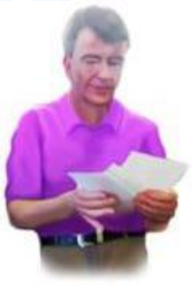

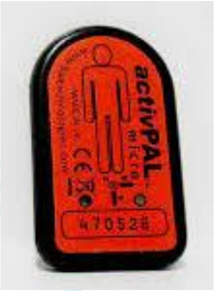

Invitation to take part in a research study

- We would like to invite you to take part in a research study.
- This leaflet tells you about the study and what your participation would involve. Please read this carefully and ask us if anything is not clear or if you would like more information.
- Please feel free to talk to other people about the study if you wish.

For peer review only

For peer review only - http://bmjopen.bmj.com/site/about/guidelines.xhtml

What is the purpose of the study?

Research has shown that too much time spent sitting (sedentary behaviour) is bad for our health. Changes in the amount of time spent sitting may improve recovery after a stroke. The RECREATE study is exploring a new approach to help people sit less, and performing an initial test to see whether the new approach is better than "usual care".

In this study, stroke services will be randomly allocated to either provide the new approach or continue with their "usual care". This will enable us to compare the new approach with standard practices.

Stroke survivors have been involved throughout the design of this study, to ensure it is well designed and with people such as yourself in mind.

Why have I been invited to take part?

You are being invited to take part because you have had a stroke and are being cared for by a stroke service taking part in our study.

Do I have to take part?

No, it is up to you. You do not have to give a reason if you decide not to take part. If you decide not to take part in this study, the standard of care that you receive will not be affected in any way.

What will be involved if I agree to take part?

- A researcher will ask you some questions about yourself (e.g., your age, height and weight, whether you live alone) and will measure your blood pressure.

- You will be asked to complete some questionnaires on up to 4 occasions over the next 24 months. The first questionnaires will be provided to you when you first agree to take part in the study. We will send you the second and third questionnaires around 6 and 12 months later. We may invite you to complete a fourth questionnaire around 24 months after you agreed to take part. On each occasion these may take approximately 60 minutes to complete.

For peer review only - http://bmjopen.bmj.com/site/about/guidelines.xhtml

The questions will be about how you feel, what you are able to do, and use of care services, such as GP or hospital visits. It will help us understand more about your experience and the impact of your stroke over 2 years.

Someone, a friend or relative, can help you fill in the questionnaires but you must think of your own responses. This way your answers will definitely be your own. You will be provided with a pre-paid envelope to return your questionnaire.

- As well as completing the questionnaire booklet, you will be asked to wear an activity monitor and complete a sleep diary for nine days on each of the occasions.

  The activity monitor is extremely small, slim, and very light. The monitor attaches to your thigh and uses information about the position of the thigh to determine body posture (i.e., sitting/lying, standing and stepping).

  A researcher will show you how to attach the monitor and help you to do this. You will be provided with a pre-paid envelope to return the monitor and sleep diary at the end of each monitoring period. (Further information is provided in the activPAL Information Leaflet.)

- You will be sent a letter around 12 weeks after you leave hospital and may be asked to answer some questions about your recent experiences.

- You will be sent newsletters to keep you updated with the study.

What else will happen if I decide to take part?

- A researcher will access relevant parts of your medical and care records so that we can gain information about the treatment and services being provided to you. We may also collect further health and treatment information from electronic databases (e.g. NHS Digital and the Sentinel Stroke National Audit Programme).

- A researcher may telephone you, send you text messages or email you about this study (for example if we do not receive the questionnaires or activity monitor back from you).

- A researcher may ask you if they can observe some of the care you are being provided with (for example, therapy treatment) and / or contact you in a few months'

For peer review only - http://bmjopen.bmj.com/site/about/guidelines.xhtml

time to ask if you would like to take part in an interview so we can gain your views on the treatment and services you have received. The interview would take place at a time and a location that is convenient for you (probably your own home or via telephone) and take about an hour.

The researcher would contact you separately about this. Not everyone will be observed or contacted for an interview. If you are, you can agree or refuse to take part in the observation or interview at that point.

What are the possible benefits of taking part?

Being involved is unlikely to benefit you directly. However, it may help improve future services and support for people who have had a stroke.

What are the possible disadvantages and risks of taking part?

You may find wearing the activity monitor inconvenient. There is a small chance that it might irritate your skin. You may find some of the topics covered in the questionnaires upsetting. You do not have to answer any questions you do not wish to.

Will my taking part in the study be kept confidential?

If you decide to participate, the information collected about you will be handled strictly in accordance with the consent that you have given and also the 2018 Data Protection Act.

Members of the RECREATE research team and other authorised personnel will use your identifiable information (name, date of birth, address, telephone number and email address) to contact you about the study; make sure that relevant information about the study is recorded for your care and to oversee the quality of the study.

Your GP will be informed about your involvement in this study. Further information about how the data we collect is used is provided in the supplementary information sheet. Please read this information carefully and discuss it with your relatives and friends if you wish. Our privacy notice is available at: https://www.bradfordhospitals.nhs.uk/privacy-statement/.

For peer review only - http://bmjopen.bmj.com/site/about/guidelines.xhtml

Ask a member of the research team if anything is unclear, or if you would like more information.

What will happen if I do not want to carry on with the study?

You are free to withdraw from the study at any time, and do not have to give a reason. If you decide to stop taking part it will not affect the standard of care you receive.

If you withdraw from the study, the data collected from you up to that point will be used in analysing the results of the study. Unless you explicitly ask us not to, we will continue to collect information from your electronic health records.

What will happen to the results of the research study?

The results of this study will be published in a report and in academic journals. If you wish to obtain a copy of the report, or a lay summary of our findings, you can request one from the Trial Manager (contact details below) or visit our website using the following address: https://www.bradfordresearch.nhs.uk/our-research-teams/academic-unit-for-ageing-and-stroke-research/our-research/stroke-research/

It is also possible that the results may be presented at conferences and included in updates to clinical guidelines. You will not be identified in any report/publication.

What if I need more information or there is a problem?

If you need further information or have any concerns about any aspects of the study, please contact Seline Ozer, RECREATE Trial Manager Tel: 01274 383908 / 07870 543073, Email: recreate@bthft.nhs.uk in the first instance.

If you remain concerned and / or would like to discuss the study with someone independent of the research team you can contact the Patient Advice and Liaison Service (PALS) on: local office, Tel: xxxx).

In the unlikely event that something goes wrong and you are harmed during the research and / or you wish to raise a complaint on how we have handled your personal data, the normal NHS complaints procedures are available to you. Details can be obtained from your hospital or the PALS Service. There are no special compensation arrangements in place for this study.

For peer review only - http://bmjopen.bmj.com/site/about/guidelines.xhtml

In addition, you can contact the Stroke Association [0303 3033 100] for confidential advice.

Who is organising, funding and reviewing the research?

The Chief Investigator of this research is Professor Anne Forster, who is employed by the University of Leeds and is carrying out the study from the Academic Unit for Ageing and Stroke Research at Bradford Teaching Hospitals NHS Foundation Trust.

The University of Leeds Clinical Trials Research Unit (CTRU) and King's College, London are supporting the co-ordination of the study.

The research is funded by the Department of Health (National Institute for Health Research). The sponsor for the research is Bradford Teaching Hospitals NHS Foundation Trust. This study has been reviewed by Yorkshire and The Humber – Bradford Leeds Research Ethics Committee.

FUNDED BY
NIHR | National Institute for Health and Care Research

For peer review only - http://bmjopen.bmj.com/site/about/guidelines.xhtml

Appendix 3 - Supplementary information sheet for stroke survivors

[Insert hospital logo]

SUPPLEMENTARY INFORMATION SHEET FOR STROKE SURVIVORS

COMPLETE QUESTIONNAIRES and WEAR AN ACTIVITY MONITOR / OBSERVATION

Who is responsible for my data?

Bradford Teaching Hospitals NHS Foundation Trust (BTHFT) is the sponsor for this study based in the United Kingdom. We will be using information from you and your medical records in order to undertake this study and will act as “data controller”. This means that we are responsible for looking after your information and using it properly. The University of Leeds Clinical Trials Research Unit (CTRU) will be supporting us in the co-ordination of this study from its premises at the University of Leeds.

How will my data be used?

This research is in the public interest, which means our results will be used to improve the healthcare of patients in the future. Because of this, if you agree to take part in the study, we will use information about you, including sensitive information about your health and ethnicity.

Members of the research team will use your personal information (name, date of birth, address, telephone number and email address) to contact you about the research study and make sure that relevant information about the study is recorded for your care. Other authorised personnel may contact you to oversee the quality of the study.

Your data may be used in future by the organisations involved in this research for evaluation, teaching and training purposes relating to the provision of NHS care and treatment, and for academic and non-commercial research purposes. If this happens, your personal information will be removed so that no-one will be able to identify you.

For peer review only - http://bmjopen.bmj.com/site/about/guidelines.xhtml

Individuals from BTHFT and regulatory organisations may look at the information we have gathered from you and look at your medical records to check the accuracy of the research study.

Your rights to access, change, or move your information are limited as we need to manage your information in specific ways in order for the research to be reliable and accurate. If you withdraw from the study, we will keep the information about you that we have already obtained. If you take part in study observations and decide to withdraw, we will destroy any observation data collected up to that point, should you request it. To safeguard your rights, we will use the minimum amount of personally identifiable information possible.

You can find out more about how we use your information by contacting the Trial Manager (details below). The research team would be happy to forward any further queries you may have on to the Data Protection Officer.

Will my information be kept confidential?

Yes. If you decide to take part, the information collected about you will be handled strictly in accordance with the consent that you have given and also the Data Protection Act 2018.

You will be allocated a unique study number, which will be used along with your date of birth and initials to identify you instead of your name on all paper and electronic records we create for you.

If you consent to completing study questionnaires, the CTRU will hold a copy of the consent form, which will have your name on it. Your name, address, phone number(s) and email address (if you have one) will also be given to the study team at the CTRU and to authorised members of the research team. This is so that they can contact you about the study when they need to (for example, to send the questionnaires). Information may also be shared with another NHS Provider, for the purposes of data collection and follow-up, including administration of the activPAL.

Your data will be entered onto a secure database held at the CTRU. All access to data and databases will be restricted just to the staff who require access to process and analyse the data.

For peer review only - http://bmjopen.bmj.com/site/about/guidelines.xhtml

For peer review only

If you consent to the study observations, a copy of your signed consent form will be held at BTHFT. Data from the observations will be entered onto secure databases held at BTHFT and only accessed by authorised members of the research team.

Will you share any of my information?

We will share identifiable information such as initials, date of birth, gender, NHS Number and post code when requesting information from electronic health records registries (e.g. NHS Digital and the Sentinel Stroke National Audit Programme) to ensure we collect the correct information. This information will include health information, which is regarded as a special category of information. We will use this information to check if you have had any major health events during the course of the study. Information will be securely shared, with these system providers also working in accordance with the Data Protection Act 2018.

It is also possible that the information collected about you may be shared with other research teams to answer new research questions in the future. If this happened, the information would be anonymised so that no-one would be able to identify you from it.

Although the information we collect about you is confidential, should we identify something of concern about your health, or you disclose anything to us, which we feel puts you, or someone else at risk of harm, we may feel it necessary to report this to the appropriate persons. In these circumstances, the researcher would report their concerns to the appropriate clinician in the stroke service or your GP and consider contacting the person responsible for Adult Safeguarding in the NHS Trust, where appropriate. In the event that you were no longer in contact with an NHS stroke service, the researcher would report their concern to the Adult Safeguarding Team in the Social Services for your local area.

For peer review only

How will my information be stored?

BTHFT and the CTRU will be responsible for ensuring that information collected about you is kept safe and secure. Your information will only be accessed by individuals who need to do so in order to do their job. All the data you give us, including identifiable information such as name and date of birth will be securely stored for at least 10 years after the end of the study before being confidentially destroyed.

For peer review only - http://bmjopen.bmj.com/site/about/guidelines.xhtml

Where can I find out more about how my information is used?

You can find out more about how we use your information

- at www.hra.nhs.uk/information-about-patients/
- by contacting the Trial Manager by using the contact details below
- Bradford Teaching Hospitals NHS Foundation Trust Privacy Notice is available to read at https://www.bradfordhospitals.nhs.uk/privacy-statement/

What should I do if I need to update my details?

If you have a change in circumstances (i.e. move house), please contact us using the details below and we will update our records.

For peer review only

For more information please contact:

Dr Seline Ozer – RECREATE Trial Manager

Academic Unit for Ageing and Stroke Research

Bradford Institute for Health Research

Bradford Royal Infirmary

Duckworth Lane

Bradford

BD9 6RJ

Tel: 01274 383908 / 07870 543073

E-mail: recreate@bthft.nhs.uk

Thank you for taking the time to read this information.

FUNDED BY

NIHR | National Institute for Health and Care Research

For peer review only - http://bmjopen.bmj.com/site/about/guidelines.xhtml

Appendix 4 – Consent form for stroke survivors

Delete this line and add Hospital header

RECREATE

Reducing sedentary behaviour after stroke

A large-print version of this sheet is available on request

CONSENT FORM FOR STROKE SURVIVORS

COMPLETING QUESTIONNAIRES & WEARING ACTIVITY MONITOR

		Please initial the boxes
1.	I confirm that I have read and understood the Information Sheet Version 4.0 dated 05/08/2022 and the Supplementary Information Sheet, Version 6.0 dated 05/08/2022 for the above study. I have had the opportunity to ask questions and have had these answered satisfactorily.	
2.	I understand that my participation is voluntary and that I am free to withdraw at any time without giving a reason and without my medical care or legal rights being affected.	
3.	I agree for my medical and care records (primary care and hospital records), including my electronic health records to be reviewed by authorised individuals from the study team to obtain data on me.	
4.	I understand that relevant sections of my medical and care records, and data collected during the study, may be looked at by authorised members of the study team, regulatory bodies or sponsor, in order to check that the study is being carried out correctly.	
5.	I agree for my personal details (which may include my initials, date of birth, NHS number, postcode and sex) to be shared with central databases (such as, NHS Digital, Sentinel Stroke National Audit Programme) and the provider of my GP's clinical systems to obtain the electronic data held by my hospital and my GP.	
6.	I understand that my GP will be notified of my participation in this study. I give permission for a copy of this consent form to be sent to my GP.	
7.	I understand that the information collected about me will be used to support other research in the future, and may be shared anonymously with other researchers. I understand that Data Protection regulations will be observed.	
8.	I understand that confidentiality will be maintained unless there are concerns that I, or someone else, is at risk of harm.	

RECREATE ICF – SS Questionnaires Version 4.0 (05/08/2022)
ISRCTN No: 82280581 / REC reference: 19/YH/0403 / IRAS ID: 271111

For peer review only

9.	I agree to take part in the above study and to wear activity monitor(s) as discussed with members of the research team.	
10.	I agree to answer questions about my health and wellbeing.	
11.	I understand that even if I withdraw from the study, the data collected from me up to that point will be used in analysing the results of the study.	
12.	I agree for my details and a copy of this consent form (which will include my name and date of birth) to be stored by the Academic Unit for Ageing & Stroke Research / Clinical Trials Research Unit, University of Leeds for the purposes of this study.	

The following point is OPTIONAL

Even if you agree to take part in this study, you do not have to agree to this statement.

Please tick (✓)

		Yes	No
13.	I am happy to be contacted over the study period by a researcher from Bradford Teaching Hospitals NHS Foundation Trust to discuss taking part in an interview to discuss my experiences of the treatment I have received	☐	☐

PARTICIPANT:

NAME (CAPITALS)	DATE	SIGNATURE

WITNESS (if required):

NAME (CAPITALS)	DATE	SIGNATURE

RESEARCHER:

NAME (CAPITALS)	DATE	SIGNATURE

(1 copy for the participant; 1 copy for the AUASR / CTRU; 1 copy held in patient notes, original stored in Investigator Site File)

For researcher use only

Participant ID (Site no / Trial no)	
Participant Date of Birth	
Participant Initials	

FUNDED BY NIHR | National Institute for Health and Care Research

For peer review only - http://bmjopen.bmj.com/site/about/guidelines.xhtml

SPIRIT
STANDARD PROTOCOL ITEMS: RECOMMENDATIONS FOR INTERVENTIONAL TRIALS

SPIRIT 2013 Checklist: Recommended items to address in a clinical trial protocol and related documents*

Section/item	Item No	Description	Addressed on page number
**Administrative information**			
Title	1	Descriptive title identifying the study design, population, interventions, and, if applicable, trial acronym	Page 1
Trial registration	2a	Trial identifier and registry name. If not yet registered, name of intended registry	Page 2
	2b	All items from the World Health Organization Trial Registration Data Set	Throughout
Protocol version	3	Date and version identifier	Page 26
Funding	4	Sources and types of financial, material, and other support	Page 33
Roles and responsibilities	5a	Names, affiliations, and roles of protocol contributors	Page 1
	5b	Name and contact information for the trial sponsor	Page 33
	5c	Role of study sponsor and funders, if any, in study design; collection, management, analysis, and interpretation of data; writing of the report; and the decision to submit the report for publication, including whether they will have ultimate authority over any of these activities	Page 33
	5d	Composition, roles, and responsibilities of the coordinating centre, steering committee, endpoint adjudication committee, data management team, and other individuals or groups overseeing the trial, if applicable (see Item 21a for data monitoring committee)	Page 24 - 25
**Introduction**			
Background and rationale	6a	Description of research question and justification for undertaking the trial, including summary of relevant studies (published and unpublished) examining benefits and harms for each intervention	Page 3 - 5
	6b	Explanation for choice of comparators	Page 9
Objectives	7	Specific objectives or hypotheses	Page 5 - 6

For peer review only

For peer review only - http://bmjopen.bmj.com/site/about/guidelines.xhtml

Trial design	8	Description of trial design including type of trial (eg, parallel group, crossover, factorial, single group), allocation ratio, and framework (eg, superiority, equivalence, noninferiority, exploratory)	Page 6
**Methods: Participants, interventions, and outcomes**			
Study setting	9	Description of study settings (eg, community clinic, academic hospital) and list of countries where data will be collected. Reference to where list of study sites can be obtained	Page 7
Eligibility criteria	10	Inclusion and exclusion criteria for participants. If applicable, eligibility criteria for study centres and individuals who will perform the interventions (eg, surgeons, psychotherapists)	Page 7 - 8
Interventions	11a	Interventions for each group with sufficient detail to allow replication, including how and when they will be administered	Page 9 -10
	11b	Criteria for discontinuing or modifying allocated interventions for a given trial participant (eg, drug dose change in response to harms, participant request, or improving/worsening disease)	Page 10
	11c	Strategies to improve adherence to intervention protocols, and any procedures for monitoring adherence (eg, drug tablet return, laboratory tests)	Page 10 - 11
	11d	Relevant concomitant care and interventions that are permitted or prohibited during the trial	Page 9
Outcomes	12	Primary, secondary, and other outcomes, including the specific measurement variable (eg, systolic blood pressure), analysis metric (eg, change from baseline, final value, time to event), method of aggregation (eg, median, proportion), and time point for each outcome. Explanation of the clinical relevance of chosen efficacy and harm outcomes is strongly recommended	Page 11 - 15
Participant timeline	13	Time schedule of enrolment, interventions (including any run-ins and washouts), assessments, and visits for participants. A schematic diagram is highly recommended (see Figure)	Page 17 - 19
Sample size	14	Estimated number of participants needed to achieve study objectives and how it was determined, including clinical and statistical assumptions supporting any sample size calculations	Page 20
Recruitment	15	Strategies for achieving adequate participant enrolment to reach target sample size	Page 17 – 19
**Methods: Assignment of interventions (for controlled trials)**			
Allocation:			
Sequence generation	16a	Method of generating the allocation sequence (eg, computer-generated random numbers), and list of any factors for stratification. To reduce predictability of a random sequence, details of any planned restriction (eg, blocking) should be provided in a separate document that is unavailable to those who enrol participants or assign interventions	Page 15
Allocation concealment mechanism	16b	Mechanism of implementing the allocation sequence (eg, central telephone; sequentially numbered, opaque, sealed envelopes), describing any steps to conceal the sequence until interventions are assigned	Page 16

For peer review only - http://bmjopen.bmj.com/site/about/guidelines.xhtml

For peer review only

Implementation	16c	Who will generate the allocation sequence, who will enrol participants, and who will assign participants to interventions	Page 15
Blinding (masking)	17a	Who will be blinded after assignment to interventions (eg, trial participants, care providers, outcome assessors, data analysts), and how	Page 16
	17b	If blinded, circumstances under which unblinding is permissible, and procedure for revealing a participant's allocated intervention during the trial	n/a
**Methods: Data collection, management, and analysis**			
Data collection methods	18a	Plans for assessment and collection of outcome, baseline, and other trial data, including any related processes to promote data quality (eg, duplicate measurements, training of assessors) and a description of study instruments (eg, questionnaires, laboratory tests) along with their reliability and validity, if known. Reference to where data collection forms can be found, if not in the protocol	Page 11 – 15, 18 - 20 , Appendix 1
	18b	Plans to promote participant retention and complete follow-up, including list of any outcome data to be collected for participants who discontinue or deviate from intervention protocols	Page 19 - 20
Data management	19	Plans for data entry, coding, security, and storage, including any related processes to promote data quality (eg, double data entry; range checks for data values). Reference to where details of data management procedures can be found, if not in the protocol	Page 20
Statistical methods	20a	Statistical methods for analysing primary and secondary outcomes. Reference to where other details of the statistical analysis plan can be found, if not in the protocol	Page 20 - 23
	20b	Methods for any additional analyses (eg, subgroup and adjusted analyses)	Page 22
	20c	Definition of analysis population relating to protocol non-adherence (eg, as randomised analysis), and any statistical methods to handle missing data (eg, multiple imputation)	Page 20, 23
**Methods: Monitoring**			
Data monitoring	21a	Composition of data monitoring committee (DMC); summary of its role and reporting structure; statement of whether it is independent from the sponsor and competing interests; and reference to where further details about its charter can be found, if not in the protocol. Alternatively, an explanation of why a DMC is not needed	Page 24- 25
	21b	Description of any interim analyses and stopping guidelines, including who will have access to these interim results and make the final decision to terminate the trial	n/a - No formal interim or sub-group analyses are planned, and final analysis will take place when

For peer review only - http://bmjopen.bmj.com/site/about/guidelines.xhtml

For peer review only

			all available data have been received.
Harms	22	Plans for collecting, assessing, reporting, and managing solicited and spontaneously reported adverse events and other unintended effects of trial interventions or trial conduct	Page 25
Auditing	23	Frequency and procedures for auditing trial conduct, if any, and whether the process will be independent from investigators and the sponsor	Page 20, 24-25, 33
**Ethics and dissemination**			
Research ethics approval	24	Plans for seeking research ethics committee/institutional review board (REC/IRB) approval	Page 26
Protocol amendments	25	Plans for communicating important protocol modifications (eg, changes to eligibility criteria, outcomes, analyses) to relevant parties (eg, investigators, REC/IRBs, trial participants, trial registries, journals, regulators)	Page 26
Consent or assent	26a	Who will obtain informed consent or assent from potential trial participants or authorised surrogates, and how (see Item 32)	Page 17 -18
	26b	Additional consent provisions for collection and use of participant data and biological specimens in ancillary studies, if applicable	n/a
Confidentiality	27	How personal information about potential and enrolled participants will be collected, shared, and maintained in order to protect confidentiality before, during, and after the trial	Page 20
Declaration of interests	28	Financial and other competing interests for principal investigators for the overall trial and each study site	Page 34
Access to data	29	Statement of who will have access to the final trial dataset, and disclosure of contractual agreements that limit such access for investigators	Page 33 - 34
Ancillary and post-trial care	30	Provisions, if any, for ancillary and post-trial care, and for compensation to those who suffer harm from trial participation	n/a
Dissemination policy	31a	Plans for investigators and sponsor to communicate trial results to participants, healthcare professionals, the public, and other relevant groups (eg, via publication, reporting in results databases, or other data sharing arrangements), including any publication restrictions	Page 26
	31b	Authorship eligibility guidelines and any intended use of professional writers	Page 26
	31c	Plans, if any, for granting public access to the full protocol, participant-level dataset, and statistical code	Page 33 - 34
**Appendices**			
Informed consent	32	Model consent form and other related documentation given to participants and authorised surrogates	Appendix 2, 3 and

For peer review only - http://bmjopen.bmj.com/site/about/guidelines.xhtml

For peer review only

materials			4
Biological specimens	33	Plans for collection, laboratory evaluation, and storage of biological specimens for genetic or molecular analysis in the current trial and for future use in ancillary studies, if applicable	n/a

*It is strongly recommended that this checklist be read in conjunction with the SPIRIT 2013 Explanation & Elaboration for important clarification on the items. Amendments to the protocol should be tracked and dated. The SPIRIT checklist is copyrighted by the SPIRIT Group under the Creative Commons "Attribution-NonCommercial-NoDerivs 3.0 Unported" license.

For peer review only
